# Rewiring the pneumococcal capsule pathway for investigating glycosyltransferase specificity and genetic glycoengineering

**DOI:** 10.1126/sciadv.adi8157

**Published:** 2023-09-06

**Authors:** Tong Su, Wan-Zhen Chua, Yao Liu, Jingsong Fan, Si-Yin Tan, Dai-wen Yang, Lok-To Sham

**Affiliations:** ^1^ Infectious Diseases Translational Research Programme and Department of Microbiology and Immunology, Yong Loo Lin School of Medicine, National University of Singapore, Singapore 117545, Singapore.; ^2^ Department of Biological Sciences, Faculty of Science, National University of Singapore, Singapore 117545, Singapore.; ^3^ Infectious Diseases Translational Research Programme and Department of Microbiology and Immunology, Yong Loo Lin School of Medicine, National University of Singapore, Singapore 117545, Singapore.

## Abstract

Virtually all living cells are covered with glycans. Their structures are primarily controlled by the specificities of glycosyltransferases (GTs). GTs typically adopt one of the three folds, namely, GT-A, GT-B, and GT-C. However, what defines their specificities remain poorly understood. Here, we developed a genetic glycoengineering platform by reprogramming the capsular polysaccharide pathways in *Streptococcus pneumoniae* to interrogate GT specificity and manipulate glycan structures. Our findings suggest that the central cleft of GT-B enzymes is important for determining acceptor specificity. The constraint of the glycoengineering platform was partially alleviated when the specificity of the precursor transporter was reduced, indicating that the transporter contributes to the overall fidelity of glycan synthesis. We also modified the pneumococcal capsule to produce several medically important mammalian glycans, as well as demonstrated the importance of regiochemistry in a glycosidic linkage on binding lung epithelial cells. Our work provided mechanistic insights into GT specificity and an approach for investigating glycan functions.

## Teaser

A genetic glycoengineering platform is reported for studying glycosyltransferase and producing glycans.

## INTRODUCTION

Unlike nucleic acid and protein synthesis, glycan synthesis is template-free and lacks any proofreading mechanism. In general, it is initiated in the cytoplasm or the lumen of the Golgi apparatus. The precursors are then transported across the membrane before the synthesis is completed. Thus, the fidelity of glycan production is mainly determined by two factors. Specificities of glycosyltransferases (GTs) ensure that the final glycan product is faithfully synthesized (*1*–*3*). In addition, the precursor transporter (or the “flippase”) presumably serves as a molecular checkpoint by retaining the unfinished products in another cellular compartment (*4*–*6*). In prokaryotes, glycans such as capsular polysaccharides (CPSs) are instrumental in evading the host immune system (*7*, *8*). Frequently attacked by the host, pathogens are capable of varying the CPS structure to modify their surface antigenicity (*9*). For example, the human respiratory pathogen *Streptococcus pneumoniae* (pneumococcus) as a species can produce at least 104 types of CPSs (*7*, *10*). As *S. pneumoniae* is naturally competent, strains can recombine or exchange their capsule genes, contributing to the extreme diversity and complexity of CPSs. This poses a challenge for developing vaccines against the capsule (*11*), as it is difficult to include numerous types of CPS in a single formulation.

To produce clinically important glycans for immunization (*12*) and therapeutic uses (*13*–*15*), chemoenzymatic glycoengineering approaches have been developed, which harness purified GTs to reproduce the regio- and stereochemistry of glycosidic linkages. A major bottleneck in chemoenzymatic synthesis of glycans is the availability of GTs with well-defined specificities. GT specificity was determined mostly by biochemical reconstitution, which is technically challenging and labor-intensive (*16*). High-throughput techniques have been developed to expedite assignments of GT activities, such as combining cell-free protein synthesis with mass spectrometry on a functionalized gold monolayer (GlycoScores) (*17*) and using isotopomer assembly (*18*). However, these approaches require purified enzymes and radioactive/functionalized substrates, which are often difficult to obtain. Inferring GT specificity with machine learning is still in its infancy. In addition, it requires large empirical datasets for training algorithms (*19*, *20*). To this end, the emerging field of synthetic glycobiology offers an exciting alternative for elucidating GT specificity (*21*–*23*). For example, many members of the human glycome were introduced and displayed on cell lines, enabling targeted genetic glycoengineering and functional glycomics (*24*). Nevertheless, many elongation, branching, and capping GTs in eukaryotes have relaxed specificities (*25*). Consequently, the engineered cell lines produce a complex mixture of glycoforms, complicating downstream analyses and their utility in producing therapeutic glycans (*24*). In contrast, despite the enormous diversity of the prokaryotic glycomes (*2*), bacterial surface glycans are relatively homogeneous at the single-cell level. For example, a single glycoform is often detected when purified pneumococcal CPSs are examined by nuclear magnetic resonance (NMR) spectrometry experiments (*7*, *8*). Thus, we wondered whether bacterial glycan synthesis pathways can be developed into a glycoengineering platform, as they can use more than 140 types of monosaccharide building blocks and are usually more genetically tractable (*2*).

**Fig. 1.**
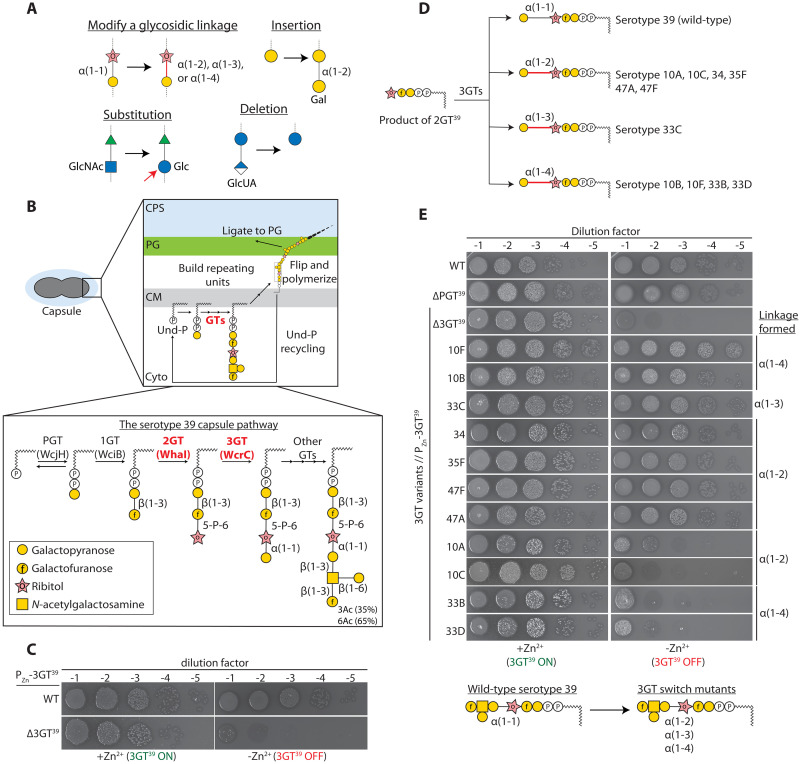
Genetic glycoengineering in bacteria. (**A**) Reprogramming the pneumococcal capsule pathway to produce structurally related glycans. Glycosidic linkages were modified (top left), and sugar residues were substituted (bottom left), inserted (top right), and deleted (bottom left). (**B**) Capsule synthesis pathway of serotype 39. The repeating units are assembled on a lipid carrier Und-P by the activities of GTs in the cytoplasm. The lipid-linked precursor is then flipped, polymerized, and ligated to the cell wall peptidoglycan (PG). Und-P released is recycled for another round of synthesis. As Und-P is shared with the PG synthesis pathway, interruptions of the pathway result in the sequestration of Und-P and killing of the cell. For simplicity, GTs are annotated based on the steps they catalyze. The second (2GT; WhaI) and the third (3GT; WcrC) GTs are highlighted in red as they are the focus of this study. (**C**) 3GT in serotype 39 (3GT^39^) is essential. Strains NUS1223 [P_Zn_-3GT^39^] and NUS1487 [∆3GT^39^ // P_Zn_-3GT^39^] were grown in BHI broth supplemented with Zn^2+^ overnight. Cells were collected by centrifugation, washed, and normalized. Cultures were then serially diluted and spotted on blood agar plates with or without Zn^2+^ supplements, followed by incubation overnight before imaging. (**D**) The product of 2GT^39^ can be modified by 3GTs of the indicated serotypes to introduce a change in the regiochemistry of the third glycosidic bond. (**E**) Strains NUS0253 [WT; isogenic serotype 39 capsule-switched mutant], NUS1365 [∆PGT], and derivatives of strain NUS1303 [∆3GT^39^::P-SweetJanus P_Zn_-3GT^39^] that harbor the noncognate 3GT indicated were grown, serially diluted, and spotted on blood agar plates as described in (C) and imaged after overnight incubation at 37°C in 5% CO_2_. (C and E) Representative images from three biological replicates. See also figs. S1 to S5.

Here, we show that the pneumococcal CPS pathway can be rewired for interrogating GT specificity and manipulating glycan structures for mechanistic studies (Fig. 1A). By exploiting the conditional essentiality of the pneumococcal CPS pathway, we demonstrate that the outcome of glycoengineering could be inferred from cell viability and encapsulation before conducting downstream biochemical validations. As a proof of concept, we systematically modified two regioisomeric linkages. Besides, we inserted, deleted, and substituted carbohydrate residues, generating 30 unique but structurally similar glycan analogs. We also identified residues that likely govern acceptor selection in a major class of GTs (i.e., GT-B). Additionally, we show that constraints of genetic glycoengineering could be partially overcome by relaxing the specificity of precursor transporters, thus providing experimental evidence that the flippase controls the quality of the glycan synthesized. Contrary to the paradigm, we demonstrate that the negative charge of the CPS plays a relatively minor role in attachment to the lung epithelial cells. On the other hand, α1-3 and α1-4 linkages at the second position seem to promote adhesion in an otherwise identical CPS. We also produced the α-Gal epitope, the H antigen, and the Lewis X antigen using the serotype 14 CPS as the starting material. We envision that this glycoengineering platform can be adopted in multiple species for producing carbohydrates and studying their biological functions.

## RESULTS

### Rationale

We selected *S. pneumoniae* as our model because it is genetically tractable with modular CPS synthesis pathways (*7*). Many CPSs in pneumococcus are produced by the Wzx/Wzy-dependent mechanism. Synthesis is initiated on a polyprenol lipid carrier called undecaprenyl phosphate (Und-P) (Fig. 1B), on which components (sugars, pyruvate, glycerol, etc.) are added sequentially to form the repeating unit. The completed lipid-linked precursor is then flipped across the cell membrane, polymerized, and attached to peptidoglycan (*7*, *26*, *27*). Since Und-P is shared between multiple glycan synthesis pathways (e.g., capsule, teichoic acids, and peptidoglycan), premature termination of capsule synthesis causes cell lysis, likely because it halts the recycling of Und-P (Fig. 1C) (*28*, *29*). Consequently, the loss of GT function usually renders the cell nonviable rather than modifying the glycan structure (*29*). While this constraint may select for the fidelity of glycan synthesis, several naturally occurring albeit uncommon examples suggest that GTs can be modified or replaced to change glycan structure and function (*30*–*33*). For instance, a single amino acid change in WciP not only converts the α1-4 linkage in the pneumococcal serotype 6A CPS to α1-3 (*30*, *32*) but also significantly affects its virulence (*34*). Thus, we reasoned that the conditional essentiality of the CPS pathway, combined with the plethora of GTs in bacteria, may provide a facile way to synthesize structurally similar glycans.

### Investigating the effects of replacing 3GT^39^ on the regiochemistry of the pneumococcal capsule

The reactions catalyzed by the 388 GTs in the pneumococcal CPS pathway have been deduced based on their CAZy classification, sequence homology, and the 92 known CPS structures (*7*). From this analysis, we selected WcrC in serotype 39 as our prototype (Fig. 1B). WcrC belongs to the GT-4 family (*35*) and adapts a widely conserved GT-B fold (*1*). It installs an α1-1–linked galactose at the third ribitol residue to form a lipid-linked tetrasaccharide intermediate [Galp-(α1-1)-Rib-(5-P-6)-Galf-(β1-3)-Galp-P-Und]. Since it is the third GT in the pathway of serotype 39, we hereinafter referred to WcrC as 3GT^39^ (table S1). There are 11 GTs in other CPS pathways that install a different glycosidic linkage between the ribitol and galactose residues (Fig. 1D and fig. S1). Thus, we hypothesized that replacing 3GT^39^ with a noncognate GT might change the regiochemistry of the capsule. To test this, we constructed an isogenic capsule-switch mutant by replacing the serotype 2 *cps* locus in the prototypical strain D39W with the serotype 39 *cps* locus. Next, a merodiploid strain was constructed in which 3GT^39^ was expressed ectopically under the control of an inducible P_Zn_ promoter (fig. S2). 3GT^39^ at the native locus could be inactivated provided that cell viability was maintained by supplementing Zn^2+^ to the medium (fig. S2A). Depletion of 3GT^39^ caused cell shape defects and lysis (fig. S2, B to D), indicating that 3GT^39^ is essential (Fig. 1C). Next, we introduced the noncognate 3GTs at the native locus and tested whether they could complement 3GT^39^ [3GT variants // P_Zn_-3GT^39^] (Fig. 1D). If so, cells would survive and remain encapsulated even if Zn^2+^ was not added to the medium (figs. S2, C and D, and S3). Notably, 3GTs that installed an α1-2, α1-3, or α1-4 linkage could replace 3GT^39^ (Fig. 1E), implying that the serotype 39 CPS pathway is versatile. Consistently, serotype 39 is thought to recombine genetically with serotype 6C to form a new serotype 10D (*36*, *37*). To eliminate the possibility that the ectopic copy of 3GT^39^ could somehow be constitutively expressed, we deleted the P_Zn_-3GT^39^ cassette so that the noncognate 3GT became the sole copy of 3GT in the cell (fig. S3A and table S2). The resulting strains remained viable and encapsulated (fig. S3, B and C), suggesting that they did not accumulate secondary mutations that disrupt the early steps of capsule synthesis. However, we could not distinguish serologically the 3GT-switch mutants from the wild type using anti–serotype 39 antisera and the more discerning factor sera that are designed to separate individual serotypes (fig. S4). Thus, the changes in the glycosidic linkage were confirmed by two-dimensional (2D) NMR experiments (fig. S5 and table S3). The data suggest that the downstream GTs in the serotype 39 CPS pathway can accommodate a different glycosidic linkage at the third position. In addition, some 3GTs like 3GT^34^ can tolerate a slightly altered acceptor (Fig. 1E) since their native precursor differs slightly from that of serotype 39.

### Substrate recognition of GTs in the CPS pathway

**Fig. 2.**
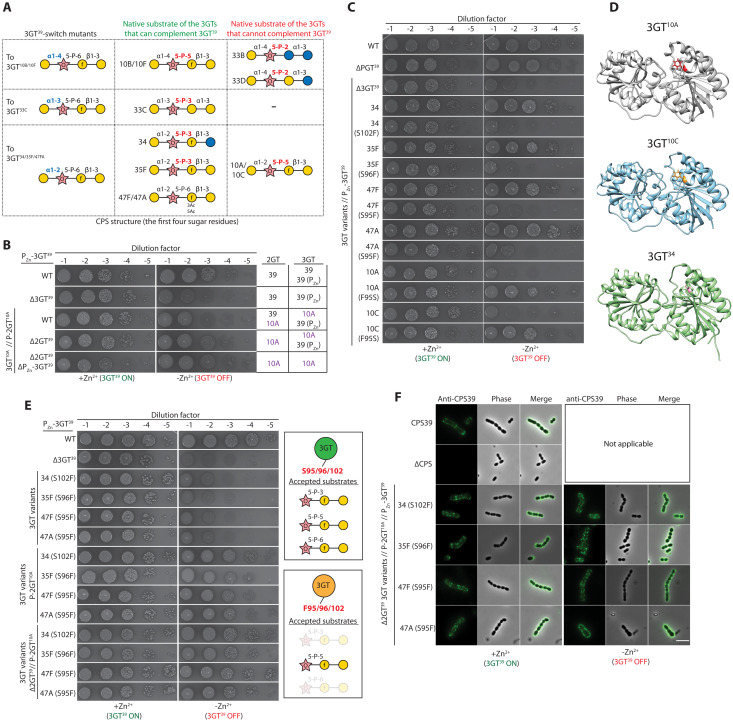
Substrate recognition of GTs in the CPS pathway. (**A**) Acceptors of 3GTs that could (in green) or could not (in red) complement 3GT^39^ and the expected CPS structures of the 3GT-switch mutants. (**B**) Modifying the second glycosidic linkage allowed 3GT^10A^ to complement 3GT^39^. Strains NUS1223 [P_Zn_-3GT^39^], NUS1487 [∆3GT^39^ // P_Zn_-3GT^39^], NUS1554 [3GT^10A^ // P_Zn_-3GT39 // P-2GT^10A^], NUS1664 [∆2GT^39^ 3GT^10A^ // P_Zn_-3GT39 // P-2GT^10A^], and NUS1776 [∆2GT^39^ 3GT^10A^ // P-2GT^10A^] were grown, serially diluted, spotted on plates, and imaged. (**C**) A residue at the cleft of the two Rossman-fold domains is crucial for substrate recognition. Strains NUS0253 [WT], NUS1365 [∆PGT^39^], and derivatives of NUS1303 [∆3GT^39^ // P_Zn_-3GT^39^] harboring the indicated 3GT variants were grown, serially diluted, spotted on plates, and imaged. (**D**) Structural models of 3GT^10A^, 3GT^10C^, and 3GT^34^ generated by AlphaFold. The phenylalanine residue in 3GT^10A^ and 3GT^10C^ and the corresponding serine residue in 3GT^34^ are highlighted with sticks. (**E**) Change of the serine residue in 3GT^34^, 3GT^35F^, 3GT^47F^, and 3GT^47A^ to phenylalanine increased their acceptor specificity. Strains NUS1223 [WT], NUS1303 [∆3GT^39^], NUS3528, NUS3529, NUS3530 and NUS3531 [P-2GT^10A^], and NUS3582, NUS3583, NUS3584, and NUS3585 [∆2GT^39^ // P-2GT^10A^] harboring P_Zn_-3GT^39^ and the indicated 3GT variants were grown, serially diluted, and spotted on plates, which were incubated overnight and imaged. (B, C, and E) Representative images from three biological replicates. (**F**) The double GT-switch mutants described in (E) remained encapsulated. The indicated strains were grown, centrifuged, and cultured in medium with or without the Zn^2+^ inducer. Cells were collected and stained with anti–serotype 39 CPS antisera. The bound immunoglobulins were labeled by Alexa Fluor 488–conjugated secondary antibodies before imaging. Representative images from two biological replicates are shown. Scale bar, 4 μm. See also figs. S6 to S14.

Not all 3GTs we tested could replace 3GT^39^, although they are expected to install a permissive linkage (Figs. 1E and 2A). For 3GT^33B^ and 3GT^33D^, this could be attributed to the substantial differences in the precursors (Fig. 2A). However, the acceptors of 3GT^10A^ and 3GT^10C^ are different from 3GT^39^ only by a single linkage (5-P-6 to 5-P-5) (Fig. 2A). We therefore hypothesized that changing this linkage should allow them to substitute 3GT^39^ by reconstructing the same acceptor. Similar to 3GT^39^, 2GT^39^ is essential (fig. S6A) and could be substituted by other 2GT variants that install a 5-P-3 and a 5-P-5 linkage, respectively (fig. S6B). We then constructed a 2GT-switch mutant by replacing 2GT^39^ with 2GT^10A^. To do this, we ectopically expressed 2GT^10A^ [P-*kan*-2GT^10A^] before deleting the native copy of 2GT^39^. Inactivation of the native 2GT^39^ is necessary for double GT switching, likely because it competes with the noncognate 2GT^10A^ for the same substrate (Fig. 2B). The double GT-switch mutant remained viable and encapsulated, indicating that 3GT^10A^ recognizes not only the ribitol residue but also the adjacent glycosidic linkage. As expected, unlike 2GT^10A^ and 3GT^10A^, introducing 2GT^33B^ and 2GT^33D^ was insufficient to allow cross-complementation of 3GT^33B^ and 3GT^33D^. These double GT-switch mutants readily accumulated suppressor mutations, resulting in the loss of CPS (fig. S7). Although the single 2GT- and 3GT-switch mutants are serologically identical to serotype 39, the double 2GT^10A^ 3GT^10A^ switch mutant cross-reacted with the anti–serogroup 10 antisera (figs. S6 to S8). It implies that this strain contains epitopes of both serogroups 10 and 39. The predicted alterations of the glycosidic linkages were confirmed by 2D NMR experiments (fig. S9 and table S4). In summary, unlike most 3GTs we tested, 3GT^10A^ has an additional layer of substrate authentication as it recognizes the glycosidic linkage adjacent to the acceptor sugar.

To investigate the mechanism of the more stringent substrate recognition by 3GT^10A^, we aligned the acceptor-binding domains of the 3GTs and identified two residues (F95 and A122 of 3GT^10A^) that may determine its specificity (fig. S10). Site-directed mutagenesis indicated that F95, but not A122, is the causative mutation because the F95S mutants of 3GT^10A^ and 3GT^10C^ gained the ability to substitute 3GT^39^ (Fig. 2C and figs. S11 and S12). The inability to complement 3GT^39^ by 3GT^10A^ and 3GT^10C^ could not be explained by the differences in their expression levels, and the F95S variation did not appreciably change the protein amount of 3GT^10A^ and 3GT^10C^ (fig. S11B). Consistently, changing the corresponding serine residues in 3GT^34^, 3GT^35F^, 3GT^47F^, and 3GT^47A^ to phenylalanine abolished cross-complementation (Fig. 2C), confirming the importance of F95 of 3GT^10A^ in substrate recognition. On the basis of the structural model of 3GT^10A^ generated by AlphaFold (*38*), F95 is located at the cleft between the two Rossmann-like domains, which is thought to be the binding site for the acceptor (Fig. 2D) (*1*). Replacing F95 in 3GT^10A^ with serine likely reduces the hydrophobicity of this substrate binding pocket, or it prevents steric hindrance of the noncognate acceptor similar to the ligand recognition mechanism of the quorum sensing receptor CqsS (*39*). Changing the analogous serine residues (S95 or S102) to phenylalanine results in 3GTs that could only accept substrates with a 5-P-5 bond at the second position (Fig. 2, E and F, and fig. S13). Thus, F95 is a critical residue for substrate recognition of 3GTs and perhaps other enzymes with a GT-B fold.

Next, we investigated whether GT-switching would result in the replacement of sugar residues. Here, 2GT^18A^ was selected as a model, which adds *N*-acetylglucosamine (GlcNAc) to the rhamnose (Rha) residue (fig. S14A). As expected, depletion of 2GT^18A^ is lethal (fig. S14B). When 2GT^18A^ was replaced by a similar enzyme 2GT^16F^ that installs a glucose (Glc) residue instead of GlcNAc, the mutant remained viable and capsule producing (fig. S14, B and C). Staining of the 2GT-switch mutant with serotype-specific factor sera revealed the expected seroconversion (fig. S14D), consistent with the substitution of the sugar residue.

### Relaxing substrate specificity of the precursor transporter allows deletions of otherwise essential GTs

**Fig. 3.**
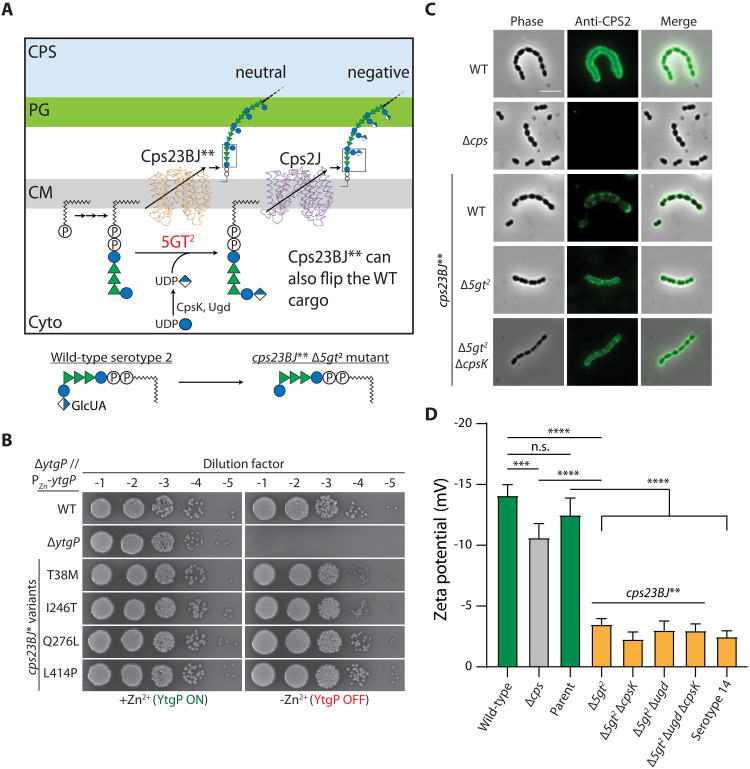
Relaxing substrate specificity of the precursor transporter allows deletions of otherwise essential GTs. (**A**) Serotype 2 CPS pathway. 5GT^2^ installs the last GlcUA residue to the lipid-linked intermediate using UDP-GlcUA generated by CpsK and Ugd as the precursor. CpsK is essential for growth likely because Ugd alone is insufficient to support capsule production. Cps23BJ** were isolated by selecting flippase variants that could transport lipid II. Because of the absence of GlcUA, the resulting capsule will lose its negative charge. (**B**) The Cps23BJ** variants support lipid II transport. Strains NUS2247 [P_Zn_-*ytgP*; WT], NUS1992 [∆*ytgP* // P_Zn_-*ytgP*], and the derivatives of NUS1992 carrying Cps23BJ** variants were grown, serially diluted, and spotted on blood agar. Plates were incubated overnight before imaging. Representative images from three biological replicates are shown. (**C**) *S. pneumoniae* harboring cps23BJ** remained encapsulated after deletions of 5GT^2^ and CpsK. Cells of strain IU1781 [WT], NUS0114 [∆*cps*], NUS2866 [cps23BJ**], NUS2987 [cps23BJ** ∆5GT^2^], and NUS3000 [cps23BJ** ∆5GT^2^ ∆*cpsK*] were labeled with anti–serotype 2 antisera and detected with Alexa Fluor 488–conjugated secondary antibodies. Representative images from two biological replicates are shown. Scale bar, 4 μm. Inactivation of 5GT^2^ is sufficient to neutralize the negatively charged cell surface. Cells of strains IU1781 [WT], NUS0114 [∆*cps*], NUS2866 [cps23BJ**; Parent], NUS2987 [cps23BJ** ∆5GT^2^], NUS3000 [cps23BJ** ∆5GT^2^ ∆*cpsK*], NUS3318 [cps23BJ** ∆5GT^2^ ∆*ugd*], NUS3319 [cps23BJ** ∆5GT^2^ ∆*ugd* ∆*cpsK*], and NUS0403 [the serotype 14 isogenic capsule-switch mutant] were grown to the mid-log phase, heat-inactivated, and washed. (**D**) The zeta potentials of the cells were then measured. The averages and standard derivations from three technical repeats are plotted. *P* values were calculated with Student’s *t* tests. n.s., not significant; ***P < 0.01; ****P < 0.001. See also figs. S15 to S18.

We hypothesized that deletions of GTs that install the terminal glycan residues on branch chains are more likely to be tolerated by the cell. As these termini are added in the later stage of the pathway, their inactivation will unlikely affect the polymerase and the GTs downstream (fig. S15). When we systematically deleted 38 GTs of this group, seven of them are dispensable for growth (table S5). For example, the ∆6GT^19C^ mutant in serotype 19C is viable, producing a capsule that is serologically indistinguishable from serotype 19B (fig. S16, A and B). However, many of the branch chain–producing GTs remained essential. We speculated that this is because some of these branch chains are recognized by the flippase and are therefore required for the efficient translocation of the precursor (Fig. 3A). CpsJ is proposed to trap the incomplete repeating units inside the cell (*4*, *5*). Breaking its specificity may release the truncated lipid-linked intermediates to the outer leaflet of the cytoplasmic membrane, thereby restoring cell viability. To test this, we first introduced flippases known to have relaxed specificity, such as Wzk (*40*) and variants of WzxC (WzxC*) (*5*), into *S. pneumoniae*. However, none of them were functional in strain D39W (table S6). Thus, we used gain-of-function CpsJ variants called Cps23BJ* (i.e., Cps23BJ^P254S^), which is shown to transport the noncognate serotype 2 cargo (*5*). Nevertheless, Cps23BJ* could not transport serotype 14 cargo and the peptidoglycan precursor lipid II, suggesting that the ability of this transporter to flip noncognate cargo is limited (table S7). To further expand the specificity of Cps23BJ*, we mutagenized cps23BJ* and isolated alleles that could flip lipid II (Fig. 3B). These Cps23BJ* variants, hereafter referred to as Cps23BJ** (e.g., Cps23BJ^P254S,I246T^), gained the ability to transport lipid II and the precursors of serotype 2, 14, and 23B CPS (Fig. 3B and fig. S17A). Strikingly, the presence of the cps23BJ** allele rendered an additional 13 GTs dispensable (table S5).

Among them were 4GT and 5GT from serotype 15C. Using the deletion mutants, we demonstrated that the 2-P-3–linked glycerol, rather than the α1-2–linked galactose, is likely the epitope that distinguishes serotype 14 and serogroup 15 (fig. S16, C and D). Another example was 5GT^2^, which installs the last glucuronic acid (GlcUA) residue to the serotype 2 CPS (Fig. 3A) (*27*). If 5GT^2^ became dispensable, cps23BJ** should also bypass the essentiality of *cps2K* because the precursor uridine-5'-diphosphoglucuronic acid (UDP)–GlcUA is no longer needed by the cell. ∆*cps2K*, with or without another UDP-glucose dehydrogenase (Ugd), was no longer lethal in the cps23BJ** background. The strain remained encapsulated, suggesting that there is no suppressor mutation in the early steps of the pathway (Fig. 3C; fig. S17, B and C; and tables S8 and S9). In addition, we showed that the cps23BJ** allele did not suppress the essentiality of *cps2H* (encoding the polymerase) because the polymerase is downstream of the flippase in the pathway (table S10). As GlcUA is the only negatively charged residue of the serotype 2 CPS, ∆*5gt2* will likely change the surface charge of the cell. Zeta potential measurements confirmed that unlike the wild-type and the unencapsulated mutant (Fig. 3D), the surface charge of the ∆*5gt2* mutant is comparable to cells coated with a neutral serotype 14 CPS (Fig. 3D and fig. S17D). As expected, deletions of *cps2K* and *ugd* did not increase the zeta potential further (fig. S17D), suggesting that no other GT in the cell could substitute 5GT^2^. The loss of GlcUA was also validated by glycan composition analysis (fig. S17, E and F) and polymerase chain reaction (PCR) (fig. S18). Our results indicated that reducing the specificity of the flippase can bypass the essentiality of late GTs.

### Installing an additional sugar residue on the CPS

**Fig. 4.**
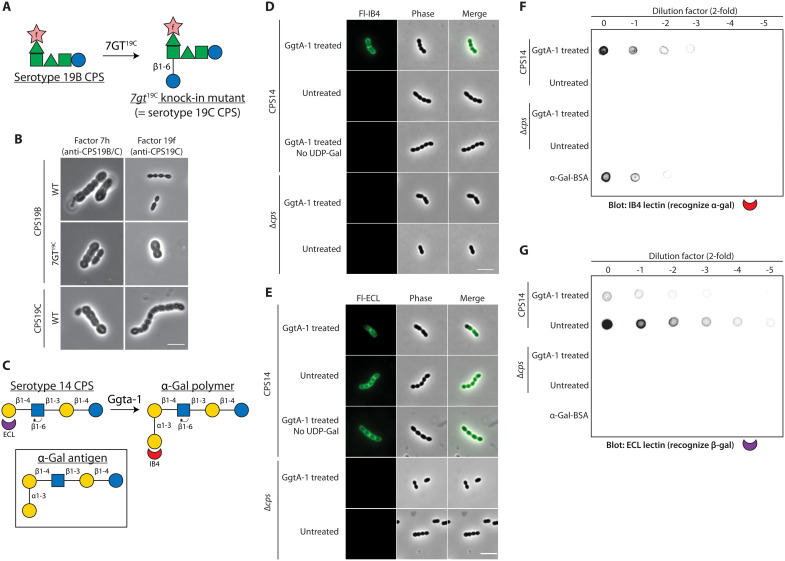
Installing an additional sugar residue on the CPS. (**A**) Serotype 19B and 19C capsules are similar except that the latter has an additional Glc residue installed by 7GT^19C^. (**B**) Introducing 7GT^19C^ into the isogenic capsule-switch mutant of serotype 19B is sufficient for seroconversion by installing an additional Glc residue. Strains NUS0317 [the serotype 19B isogenic capsule-switch mutant; WT], NUS2064 [P-7GT^19C^], and NUS0316 [the serotype 19C isogenic capsule-switch mutant; WT] were grown and stained with factor sera 7h (recognizing serotype 19B and 19C capsules) and 19f (recognizing serotype 19C capsule only). Cells were visualized by microscopy, and the capsule can be visualized as halos. Representative images from three biological replicates are shown. (**C**) Modifying the serotype 14 capsule to produce the α-Gal antigen. The serotype 14 CPS structurally resembles paragloboside (nLC4). Glycoengineering of this capsule with Ggta-1 thus produced the α-Gal antigen as a polymer, which can be detected by staining with the IB4 lectin. In contrast, the serotype 14 capsule can be detected by the ECL lectin. (**D**) Strains NUS0403 [serotype 14 isogenic capsule-switch mutant; CPS14] and NUS0114 [∆*cps*] were grown. Cells were harvested, heat-killed, and treated with Ggta-1 and UDP-Gal where indicated. The modified capsule was detected by the fluorescein-labeled IB4 lectin and imaged. Representative images from two biological replicates are shown. Scale bar, 4 μm. (**E**) The strains listed in (D) were stained with fluorescein-labeled ECL. Scale bar, 4 μm. IB4 binds to the Ggta-1–modified serotype 14 capsule. Heat-killed cells of strains NUS0403 and NUS0114 were treated with Ggta-1 where indicated, serially diluted, and spotted on a nitrocellulose membrane. The membrane was blotted with IB4 (**F**) or ECL (**G**) and imaged. α-Gal conjugated to bovine serum albumin (α-Gal-BSA) was included as a positive control. Representative images from three biological replicates are shown. See also figs. S19 and S20.

**Fig. 5.**
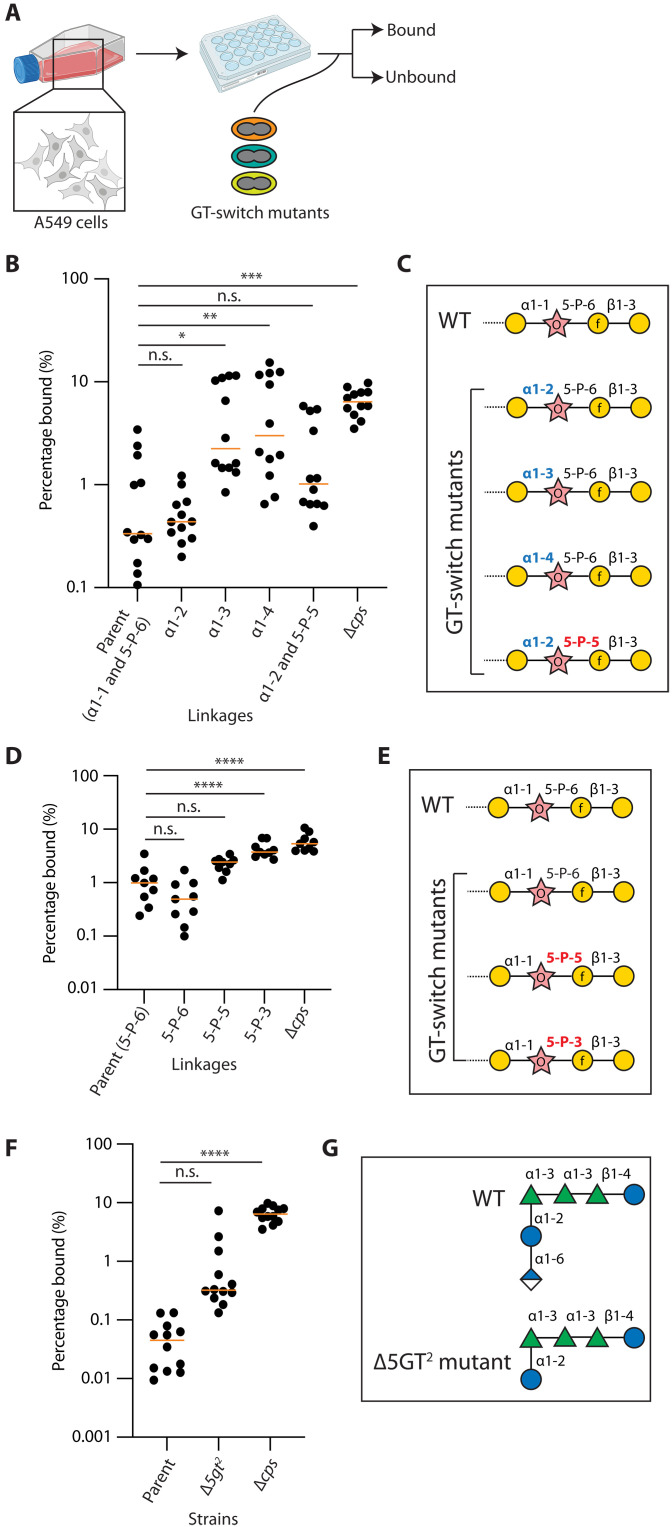
α1-3 and α1-4 linkages between ribitol and galactose facilitate A549 binding. (**A**) Epithelial cell binding assay. A549 cells were grown and seeded on plates. After overnight incubation, pneumococci were added and the coculturing continued. Unbound pneumococci were removed by decanting the medium, whereas the bound pneumococci were recovered by detaching the A549 cells. The two fractions were spread on plates and counted. (**B**) Strains NUS0253 [the serotype 39 capsule-switch mutant; parent; α1-1 and 5-P-6], NUS1429 [α1-2], NUS1409 [α1-3], NUS1415 [α1-4], NUS1776 [α1-2 and 5-P-5], and NUS0483 [∆*cps*] were grown and inoculated to A549 cells at a multiplicity of infection (MOI) of 10. (**C**) Structures of the CPS displayed on the strains used in the binding assay described in (B). For simplicity, only the four residues at the reducing end are shown. (**D**) Strains NUS0253 [parent; 5-P-6], NUS1466 [2GT^47F^; 5-P-6], NUS2155 [2GT^10B^; 5-P-5], NUS2153 [2GT^34^; 5-P-3], and NUS0483 [∆*cps*] were grown to the exponential phase and inoculated to A549 to measure binding as described in (B). (**E**) Structures of the CPS displayed on the strains used in the binding assay described in (D). (**F**) Strains IU1781 [*rpsL1*; the serotype 2 parent strain], NUS3000 [∆*5gt2*], and NUS0463 were grown to the exponential phase and inoculated to A549 to measure binding as described in (B). (**G**) Structures of the CPS displayed on the strains used in the binding assay described in (F). The mean percentages of bacteria bound (orange line) from four biological replicates are shown in (B), (D), and (F), and each replicate has three technical repeats. *P* values were calculated with Student’s *t* tests. *P < 0.05; **P < 0.01; ***P < 0.005, ****P < 0.001. All glycan structures were drawn in the Symbol Nomenclature for Glycans (SNFG) format. See also fig. S21.

Next, we tested whether introducing an additional GT would add a sugar residue to the pneumococcal CPS. To do this, we ectopically expressed 7GT^19C^ in a serotype 19B capsule-switch mutant, leading to the expected seroconversion to serotype 19C (Fig. 4, A and B). In the course of our study, we noticed that the serotype 14 CPS structurally resembles paragloboside (nLC4). nLC4 is the precursor of the Galili antigen (α-Gal) (Fig. 5C), the primary cause of hyperacute rejection during xenotransplantation (*41*). To test whether the serotype 14 CPS can be converted to an α-Gal–containing polysaccharide, we introduced bovine α-1,3 galactosyltransferase Ggta-1 (*42*) to a capsule-switch mutant of serotype 14. However, we did not observe any change in the capsule, possibly because Ggta-1 was not expressed or was nonfunctional in *S. pneumoniae*. Thus, we treated heat-killed pneumococci (Fig. 4, D to G), purified cell wall, or purified CPS (fig. S19, A to C) with purified Ggta-1. The presence of an additional α-1,3–linked galactose and the concomitant loss of the β-1,4 galactose termini were confirmed by staining with appropriate lectins (Fig. 4, D to G, and fig. S19, D and E). Notably, the specific activity of Ggta-1 was much higher than the previously reported value (381 versus 10 units per milligram of protein) (fig. S19C) (*42*), likely because the serotype 14 CPS structurally resembles its native substrate.

To illustrate that this approach is likely generalizable, we produced the Lewis X antigen and the blood group H antigen using the serotype 14 CPS as the starting material (fig. S20A). Heat-killed cells were treated with recombinant human fucosyltransferase 2 (Fut2) to install an α1-2–linked fucose at the galactose residue of the side chain, thereby producing a polymer of the blood group H antigen. Consistently, the treated cells reacted with the anti–type II blood group H antibodies (fig. S20B). In addition, Fut3 from human or *Helicobacter pylori* fucosyltransferase (HpFT) was used to add an additional α1-3–linked fucose to the GlcNAc residue. Dot blotting with lectins confirmed the presence of the fucose residues, suggesting that the Lewis X antigen was generated (fig. S20, C to F). These experiments indicate that the CPS can be exploited as intermediates to generate useful glycans. Ongoing work is to introduce these GTs into pneumococcus and perform structural analyses of these biochemically modified glycans.

### α1-3 and α1-4 linkages between ribitol and galactose facilitate A549 binding

We next investigated how the attachment of pneumococci on lung epithelial cells (A549) would be affected by varying the CPS structure (Fig. 5A). We showed that coculturing A549 cells with pneumococci for 5 hours did not affect cell viability (fig. S21) and thus enabled the binding assay. Compared to the serotype 39 capsule-switch parent, strains displaying α1-3 and α1-4 CPS derivatives, but not α1-2, bound stronger to the A549 cells (Fig. 5, B and C). Modifying the second linkage to 5-P-5 did not further affect adhesion to the A549 cells in the α1-2 mutant. This result highlights the importance of the third glycosidic linkage in serotype 39 for epithelial cell attachment. The stronger adhesion observed in the α1-3 and α1-4 CPS mutants cannot be explained by a reduction in capsule production, as the amount of CPS in these mutants is comparable to the parent strain (fig. S3). We then asked whether removing the negative charge of the CPS would also impair adherence by coculturing A549 cells and the ∆*5gt2* mutant. Consistent with the lack of correlation between adhesion and the negative charge of the CPS (*43*), the ∆*5gt2* mutant bound A549 cells similarly to the parent strain (Fig. 5, D and E). These data suggest that the regiochemistry of the CPS affects attachment, whereas the negative charge likely plays a minor role in colonization.

## DISCUSSION

The lack of a facile approach to genetically engineer glycans is a major impediment to functional glycomics. Unlike proteins and RNAs, manipulations of GTs in a glycan synthesis pathway do not necessarily lead to the predicted change in glycans (*44*), as GTs can be functionally redundant or have a relaxed specificity (*25*). Here, we report a versatile genetic glycoengineering platform based on the pneumococcal capsule pathway, which allows us to edit glycan targets systematically. We demonstrate that the conditional essentiality of late CPS enzymes can be harnessed to isolate enzyme variants with altered specificity. Additionally, we systematically switched GT variants to alter the structure of CPS by adding or removing residues, or by installing a different glycosidic linkage. These otherwise identical glycans can be directly compared with the wild-type CPS in functional assays to shed light on the structure-function relationship of carbohydrates. A deeper understanding of glycans may provide new avenues for vaccines and therapeutics (*45*). While several constraints limit the utility of this platform (discussed below), this work is an important first step to illustrate the possibility of exploiting genetic logic for generating custom glycans.

Historically, genetic glycoengineering in bacteria is mostly done in *Escherichia coli* (*23*, *42*, *46*, *47*) because of its genetic tractability and ease to propagate cells. Yet, the oligosaccharide products in many cases remain dissolved in the cytoplasm. Recovering them from the many biomolecules in the cell lysate can be challenging, especially on a large scale. We argue that *S. pneumoniae* is a feasible alternative as the CPS is physically linked to peptidoglycan. Methods to purify the pneumococcal CPS are well established, making the recovery of the recombinant glycans relatively straightforward. Other than the genetic approach, we also exploited the CPS as the starting material to enzymatically synthesize useful glycans, given that the CPS is abundant, which can constitute up to 50% of the cell volume (*7*, *8*). To this end, we produced a polymer of the α-Gal antigen, the Lewis X antigen, and the blood group H antigen from the serotype 14 CPS (*46*, *48*). Ongoing work is to express these GTs in the serotype 14 background with enzymes for synthesizing precursors (e.g., GDP-fucose) such that the glycan product can be produced directly by the cell.

We identified several constraints of the genetic glycoengineering platform. One of which is the flippase. If it is unable to translocate the modified lipid-linked precursors, the cells become nonviable as interruption of CPS synthesis likely sequesters Und-P (*28*, *29*). We show that in several cases, the CPS flippase is capable of flipping an incomplete precursor because deleting the GT that installs the terminal residue of a branch chain is tolerated. If this is not the case, introducing another flippase with a more relaxed specificity (i.e., Cps23BJ**) sometimes renders the deletion mutant viable, suggesting that the flippase is a quality control factor of the pathway. However, several GTs remain strictly essential for viability despite the presence of Cps23BJ**. Thus, Cps23BJ** may not be able to flip these incomplete precursors. Alternatively, other factors such as the polymerase may limit the changes that can be made to the pneumococcal CPS. Future work will focus on generating polymerase variants with broadened specificity, as well as combining several Cps23BJ** flippase variants, to see if it further reduces the constraints of genetic glycoengineering. In addition, GTs from other bacteria can be exploited to expand the repertoire of carbohydrate residues and linkages that can be synthesized.

Besides glycoengineering, we show that the central cleft between the Rossman-like domains of GT-B enzymes likely plays a crucial role in recognizing the adjacent glycosidic linkage of the acceptor. However, some of the GTs we tested could not recognize this linkage. Why this is so remains unclear. The additional authentication process presumably ensures that the sugar residue is installed on the correct acceptor, which could be important for survival, although we have yet to understand the functional importance of the 5-P-5 linkage being recognized by 3GT^10A^ and 3GT^10C^. Ongoing work is to test whether other GT-B enzymes also use similar mechanisms to determine specificity. The highly specific GTs, together with the essentiality of the late GTs and the selectivity of the flippases, interwoven into a relatively error-free CPS synthesis pathway, which may attribute to the lack of many glycan isoforms commonly found in human glycans.

## MATERIALS AND METHODS

### Bacterial strains and growth conditions

The strains in this study are listed in table S11. Unless otherwise specified, *S. pneumoniae* cells were grown in the brain heart infusion broth (BHI) or blood agar plates (Biomed Diagnostics, 221261) at 37°C in 5% CO_2_. When indicated, antibiotics were added at final concentrations of 0.3 μg/ml for erythromycin, 250 μg/ml for kanamycin, 300 μg/ml for streptomycin, and 150 μg/ml for spectinomycin. To induce expression of the P_Zn_ promoter cassettes, cultures were supplemented with 400 μM ZnCl_2_. To counteract the cell division defects caused by Zn^2+^ toxicity (*49*), 40 μM MnCl_2_ was added to the medium.

### Strain constructions, growth measurements, and microscopy

Strains were transformed with assembled PCR amplicons or genomic DNA after inducing natural competence (*5*, *50*). Briefly, PCR products were synthesized using Phusion DNA polymerase with oligonucleotides and DNA templates listed in table S12. After purifying PCR products using the QIAGEN PCR purification kit, amplicons were assembled using Gibson’s assembly. Natural competence was induced by adding competence stimulating peptide (CSP-1), and the cells were transformed with the assembled genetic constructs. The transformants were incubated at 37°C in 5% CO_2_ for 1.5 hours and poured on blood agar plates supplemented with the appropriate antibiotics. To validate the genetic constructs, strains were diagnosed by PCR using GoTaq DNA polymerase and Sanger sequencing. Similarly, allelic exchange was done with the Janus cassette (*kan*-*rpsL*^+^) (*51*), its spectinomycin-resistant derivative (*spec*-*rpsL*^+^), or the Sweet Janus cassette (*sacB*-*kan*-*rpsL*^+^) (*52*). For growth curves, overnight cultures were grown until the optical densities at 600 nm (OD_600_) were between 0.2 to 0.4. Cultures were normalized to an OD_600_ of 0.2. Cells were collected by centrifugation at 20,000*g* for 2 min at room temperature and washed twice with 1 ml of BHI to remove ZnCl_2_ and MnCl_2_ in the medium. The cultures were diluted in BHI to a starting OD_600_ of 0.01, with and without supplementation of ZnCl_2_ and MnCl_2_. The diluted culture with a final volume of 200 μl was distributed into a 96-well plate. Growth was monitored using a Tecan microplate reader at 37°C for 20 hours. OD_600_ readings were taken every 10 min after a short pulse of shaking for 30 s. For microscopy, overnight cultures were diluted to an OD_600_ of 0.01 and grew to the mid-log phase (OD_600_ of ≈0.2). Just before the onset of cell lysis, 1 ml of culture was harvested by centrifugation at 16,100*g* for 1 min at room temperature. The supernatant fraction was removed, and the pellet was resuspended in 100 μl of BHI. Samples were mounted onto a glass slide and imaged with an IX81 phase contrast microscope (Olympus). Cell morphology and dimensions were analyzed using MicrobeJ (*53*).

### Enzyme-linked immunosorbent assay

Cross-absorbed anti-CPS antisera were prepared as described by (*28*). Cells of the unencapsulated strain NUS1365 (*rpsL1* CPS39 ∆*wcjH*) were grown in BHI until the OD_600_ was between 0.6 to 0.9. Cells were washed once with 1× phosphate-buffer saline [1× phosphate-buffered saline (PBS)] and inactivated by incubation at 56°C for 45 min. Heat-killed cells were resuspended in 1× PBS at a final OD_600_ of 0.9. For cross-adsorption, 1 μl of anti-CPS antisera (SSI Diagnostica) was mixed with 300 μl of heat-killed cells and 299 μl of 1× PBS. The mixture was incubated overnight at 4°C, and the heat-killed cells were removed by centrifugation at 20,000*g* for 2 min at room temperature and filtered through a 0.22-μm filter (Costar CLS8160). Cross-absorbed antisera were kept at 4°C until use.

Strains were grown to an OD_600_ of 0.2 to 0.4 and normalized to an OD_600_ of 0.2. Cells were harvested by centrifugation at 16,100*g* for 1 min at room temperature. Pellets were resuspended in an equal volume of 1× PBS. Next, 100 μl of the suspension was mixed with 2.5 μl of proteinase K (>600 mAU/ml; Qiagen, 19133) and incubated at 56°C for 1 hour. The supernatant was removed by centrifugation, and the pellets were resuspended in 1 ml of 50 mM bicarbonate buffer (Sigma, C3041). The samples were distributed into enzyme-linked immunosorbent assay (ELISA) plates (Thermo Fisher Scientific, 442404) and incubated overnight at 4°C to coat the plates. Alternatively, serially diluted purified CPS (SSI Diagnostica) dissolved in carbonate buffer was used as internal standards. ELISA plates were washed four times with PBST [1× PBS with 0.05% (v/v) Tween 20], blocked by adding ELISA blocking buffer [1× PBS with 1% (w/v) bovine serum albumin (BSA)], and incubated for an hour at room temperature. Plates were washed four times with PBST and incubated with anti-CPS antisera of the indicated types in blocking buffer (SSI Diagnostica) at a 1:1000 dilution. After incubation for 1 hour at room temperature, plates were washed four times with PBST, and goat anti-rabbit horseradish peroxidase (HRP) antibodies (Thermo Fisher Scientific, A16110) were added at a 1:1000 dilution in ELISA blocking buffer. Plates were washed again four times with PBST, and antibodies were detected using a colorimetric substrate *o*-phenylenediamine dihydrochloride (OPD) (Sigma, P9187-50SET) according to the manufacturer’s protocol. The CPS was quantified by extrapolating the OD_450_ values recorded with a Tecan plate reader.

### Immunoblotting

The amount of CPS was quantified by immunoblotting as described in (*28*). Briefly, cells grown to the mid-log phase were harvested by centrifugation at 16,100*g* for 1 min at room temperature and washed once with 1× PBS. Pellets were resuspended in 200 μl of protoplast buffer [0.5 M sucrose, 20 mM MgCl_2_, 20 mM Tris-HCl (pH 7)], and the suspensions were normalized based on protein concentrations. To the washed pellets, 4 μl of mutanolysin (10 U/μl; Sigma-Aldrich, catalog number M9901-10KU) and 15 μl of lysozyme (10 mg/ml; Sigma-Aldrich, catalog number 62970-1G-F) were added, and the mixture was incubated at room temperature overnight. Removal of the cell wall was monitored by the loss of the ellipsoid cell shape under phase-contrast microscopy. Samples were mixed with an equal volume of 2× Laemmli buffer with 5% 2-mercaptoethanol, separated on an SDS–polyacrylamide gel electrophoresis gel, and transferred to a polyvinylidene difluoride membrane. The membrane was blocked by incubating with 5% skim milk dissolved in PBST for an hour. FLAG-tagged proteins were detected by blotting with anti-FLAG antibodies (Sigma; 1:3000 dilution) and anti-rabbit HRP-conjugated antibodies (Thermo Fisher Scientific; 1:5000 dilution). Blots were developed by adding a chemiluminescent substrate (Thermo Fisher Scientific, 34580) and imaged.

### Immunofluorescent microscopy

Cultures were grown to an OD_600_ of 0.2 to 0.4, normalized to an OD_600_ of 0.2, and heat-inactivated at 56°C for 45 min. Cells were harvested by centrifugation at 20,000*g* for 2 min at room temperature and washed once in 1× PBS. Cells were resuspended in 100 μl of cross-absorbed anti-CPS antisera of the indicated serotype (SSI Diagnostica) at a dilution of 1:300, and the mixture was incubated on ice for 5 min. Cells were collected by centrifugation, washed twice with 1× PBS, and resuspended in 100 μl of 1× PBS. CPS was detected by incubating with anti-rabbit Alexa Fluor 488 antibodies (Thermo Fisher Scientific, A11034) at a dilution of 1:100 on ice for 5 min. Labeled cells were washed once with 1× PBS, resuspended in SlowFade mounting medium (Invitrogen, S36936), and visualized using an IX81 microscope (Olympus).

### Purification of CPS

Cultures were grown in 400 ml of BHI overnight at 37°C in 5% CO_2_. Cells were harvested by centrifugation at 3500*g* for 30 min at 4°C. Pellets were resuspended in 15 ml of 1× PBS and mixed with 10 ml of boiling 10% (w/v) SDS. The mixture was boiled for 30 min. The crude cell wall was collected by centrifugation at 10,000*g* for 10 min at room temperature, and the pellets were washed four times with water and 1× PBS to remove residual SDS. We noticed that the pellets, especially for the encapsulated strains, were very loose in water, whereas in 1× PBS the pellets aggregated and stuck on the side of the pipette tips. Thus, we first suspended the pellets in 12.5 ml of water and then added 12.4 ml of 2× PBS before centrifugation. Washed pellets were resuspended in 3 ml of 1× PBS, transferred to two microcentrifuge tubes, and centrifuged at 20,000*g* for 10 min at room temperature before resuspending in 1 ml of 50 mM NaPO_4_ (pH 7.4). The suspension was stored at 4°C overnight or used immediately for the purification of CPS. The crude cell wall was collected by centrifugation at 20,000*g* for 10 min at room temperature. The supernatant was discarded, and the pellets were resuspended in 251.2 μl of water. To the suspension, 100 μl of 0.1 M NaPO_4_ (pH 6.0), 20 μl of 10 mM MgCl_2_, 12.8 μl of mutanolysin (10 U/ml; Sigma), 8 μl of LytA (2 mg/ml) (*29*), and 8 μl of lysozyme (10 mg/ml) were added. The mixture was incubated overnight at 37°C. Proteinase K was then added to a final concentration of 1.2 mAU (Qiagen, 19133) and incubated at 50°C for an hour. The mixture was inactivated at 95°C for 10 min, and the insoluble material was removed by centrifugation at 20,000*g* for 5 min at room temperature. Supernatant fractions collected were extracted twice with an equal volume of 25:24:1 (v/v/v) phenol:chloroform:isoamyl alcohol. The mixtures were vortexed for 10 s and centrifuged at 20,000*g* for 1 min at room temperature. The top aqueous phase containing the CPS was carefully removed and transferred to a new tube. An equal volume of chloroform was added to extract phenol from the sample. The mixture was vortexed for 10 s and centrifuged again, and the top phase containing the CPS was carefully removed. The solution was dialyzed against water. The lyophilized, purified CPS was then dissolved in 6 ml of heavy water (D_2_O) before NMR and GC-MS analyses.

### Isolation of cps23BJ** variants with expanded substrate specificity

The P-*kan-cps23BJ*^P254S^ allele (*5*) was PCR mutagenized using the oligonucleotides listed in table S12 as described before (*5*). Briefly, the amplicon was synthesized with 30 cycles of amplification using error-prone GoTaq DNA polymerase to control the mutation rate. The same amplicon was produced using proofreading Phusion DNA polymerase as a negative control. PCR products were purified using the Qiagen PCR purification kit. Strain NUS1992 (*rpsL1* ∆*ytgP*::P-*erm* // ∆CEP::P_Zn_-*ytgP*) was transformed with the PCR amplicons and selected for kanamycin resistance in the presence of ZnCl_2_/MnCl_2_. Transformants were pooled, serially diluted, and selected for cps23BJ** variants that could transport the cell wall peptidoglycan precursor by omitting ZnCl_2_/MnCl_2_ supplement to the blood agar plates. Under this condition, survivors would likely have *cps23BJ* variants that could complement *ytgP*. To eliminate suppressor mutants that are outside of *cps23BJ*, transformants survived were pooled with their genomic DNA extracted, and used to transform the parent strain NUS1992. A significant increase (~100-fold) in plating efficiency was observed compared to the cells transformed with the *cps23BJ(P254S)^+^* amplicon. The *cps23BJ* alleles were then sequenced. The cps23BJ** variants were then validated by PCR, and the alleles were introduced into strain NUS1992 again to test their phenotypes.

### NMR experiments

Structures of the CPSs were determined using NMR spectroscopy as described in (*33*). Spectra were recorded on an 800-MHz Bruker Avance Neo spectrometer equipped with a Bruker TXI cryoprobe. All spectra were recorded at 310 K and referenced relative to acetone (d1H = 2.225 ppm and d13C = 30.89 ppm). The 2D NMR experiments included 1H-13C heteronuclear single quantum coherence spectroscopy (2048 × 512 complex points sampling 131- and 10.6-ms acquisition times, respectively) and 1H-13C heteronuclear multiple bond correlation (4096 × 512 complex points sampling 196- and 5.8-ms acquisition times, respectively). The 1D 1H and 13C spectra were recorded by sampling 32,768 complex points during acquisition times of 1.44 s and 688 ms, respectively. The 1D 1H and 13C NMR spectra were processed with the Topspin 4.1.4 (Bruker) software. All 2D NMR spectra were processed and analyzed with NMRPipe (*54*) and NMRFAM-SPARKY (*55*).

### Glycosyl composition analysis

Gas chromatography and mass spectrometry (GC-MS) were performed using the *O*-trimethylsilyl (TMS) methyl glycoside derivatives produced by acidic methanolysis (*56*). GC-MS was done on an Agilent 7890A GC system interfaced to a 5975B mass selective detector (MSD), using an EC-1 fused silica capillary column (30 m × 0.25 mm internal diameter). The run began for 2 min at 80°C and hold for 2 min, then for 7 min by ramping up to 140°C at 20°C/min and holding for 2 min, 37 min at 200°C at 2°C/min and holding for 37 min, and finally 43.7 min at 250°C at 30°C/min and holding for 43.7 min.

### Measurement of zeta potentials

The measurement of zeta potentials was performed as described in (*57*) with slight modifications. Briefly, cells were grown to an OD_600_ of ≈0.2 and pelleted by centrifugation at 3000*g* for 5 min at room temperature. Bacteria were washed twice, resuspended in 1× PBS, heat-killed at 65°C for 45 min, and stored at −80°C. Frozen stocks were thawed and normalized using 1× PBS to an OD_600_ of 0.1 [~10^7^ colony-forming units (CFUs)/ml]. Zeta potentials of the samples were measured at 25°C with an automated Zetasizer (Zetasizer Ultra; Malvern Panalytical) following parameters according to the manufacturer’s guide. Measurements were repeated thrice and averaged.

### Production of the α-Gal antigen from CPS

The *ggta1* gene (NC_037338.1), which encodes the α-1,3 glycosyltransferase of *Bos taurus*, was codon-optimized and synthesized (Integrated DNA Technologies). To improve the solubility and stability of Ggta1, the first 74–amino acid residues were removed by PCR (*58*) using primers O866 and O867 (table S12). The pTD68 backbone was amplified using primers O925 and O926, and the PCR products were purified. The fragments were ligated by Gibson assembly and used to transform strain NEB5-α (NEB C2987) to produce pCS190 (P_T7_-his_6_-*ggta1*∆74). Transformants were selected for ampicillin resistance and screened by PCR, and the insert was validated by sequencing. pCS190 was purified and used to transform LEMO21(λDE3) for protein expression. Strain LEMO21(λlDE3)/pCS190 was grown in 1 liter of LB supplemented with ampicillin (100 μg/ml) at 37°C with shaking. When the OD_600_ reached 0.5, cells were induced by adding isopropyl-β-d-thiogalactopyranoside to a final concentration of 0.5 mM. The induction was performed at 30°C for 2 hours. Cells were harvested by centrifugation at 5000*g* for 5 min at room temperature and resuspended in buffer A [20 mM tris-HCl (pH 8), 0.3 M NaCl, 30 mM imidazole]. Cellular proteins were released by sonication, and the debris was removed by centrifugation at 10,000*g* for 5 min at 4°C. The lysate was transferred to a new tube and clarified by another centrifugation at 16,000*g* for 5 min at 4°C. His-tagged Ggta1∆74 was bound to 2 ml of TALON agarose beads (Takara) by incubating at 4°C overnight. Beads were washed with 10 ml of buffer A, and protein was eluted with 6 ml of buffer B [20 mM tris-HCl (pH 8), 0.3 M NaCl, 500 mM imidazole]. Fractions containing His-tagged Ggta1 (~36 kDa) were polled and dialyzed against the storage buffer [50 mM tris-HCl (pH 8), 300 mM NaCl, 10% (v/v) glycerol]. Protein was frozen in liquid N_2_ and stored at −80°C until use.

UDP release from the Ggta1 reaction was measured by the UDP-Glo glycosyltransferase assay kit (Promega, V7051). Briefly, strains NUS0403 [isogenic serotype 14 capsule-switch mutant] and NUS0114 [∆*cps*] were grown in the BHI medium to OD_600_ ~ 0.7. Cells were collected by centrifugation at 10,000*g* for 5 min at room temperature and lysed by boiling in 4% (w/v) SDS dissolved in 1× PBS for 30 min. Sacculi was pelleted by centrifugation at 10,000*g* for 5 min at room temperature and washed three times with 0.5× PBS and once with water. Ggta1 was serially diluted in the storage buffer. GT reaction was performed by mixing 5 μl of 10× reaction buffer [100 mM tris-HCl (pH 7.5), 100 mM MnCl_2_, 1% (w/v) BSA], 2.5 μl of 10 mM UDP-galactose, 2.5 μl of serially diluted Ggta1 (starting from 3.25 μg), and 10 μl of sacculi. The reaction proceeded for 1 hour at 37°C before the sacculi were removed by centrifugation at 20,000*g* for 5 min at room temperature. To detect UDP, 25 μl of the supernatant fraction was mixed with 25 μl of UDP detection reagent (Promega) and incubated for 1 hour at 37°C. Luminescence was measured with a plate reader and compared with a standard curve generated by a known standard of UDP solution.

To detect the modified capsule, Ggta1-treated sacculi were washed with 1 ml of 1× PBS and resuspended in 150 μl of 1× PBS. The suspension was split into two 50-μl fractions. Fluorescein-labeled Griffonia Simplicifolia Lectin I isolectin B4 (IB4; Vector Laboratories, FL-1201-0.5) and Erythrina Cristagalli Lectin (ECL; Vector Laboratories, FL-1141-5) were added at a final concentration of 10 μg/ml and 50 μg/ml, respectively. The mixture was incubated for 10 min at room temperature, washed with 1 ml of 1× PBS, and visualized. Alternatively, sacculi modified by Ggta-1 were washed with 1× PBS and resuspended in 100 μl of water. The suspension was serially diluted with 1× PBS and spotted onto a nitrocellulose membrane. After air drying, the membrane was blocked with 1× Carbo-Free blocking solution (Vector Laboratories, SP-5040-125) for 30 min. Next, 250 μl of heat-killed unencapsulated cells (see the “Enzyme-linked immunosorbent assay” section) was added and incubated for 2 min with shaking. Lectins (IB4 or ECL) were added at a 1:5000 dilution, and the blot was incubated for 1 hour with shaking. The lectin solution was removed. The blot was washed three times with 1× PBS (each for 5 min), and the membrane was imaged.

### Production of the Lewis antigen and the blood group H antigen from CPS

Strains NUS0403 [isogenic serotype 14 capsule-switch mutant] and NUS0114 [∆*cps*] were grown in the BHI medium to an OD_600_ of ~0.4. Cells were collected by centrifugation at 20,000*g* for 1 min at room temperature, washed once with 1 ml of 1× PBS, and heat-inactivated at 65°C for 45 min. Purified human α1,2 fucosyltransferase (Fut2; 7770-GT-020), human α1,3 fucosyltransferase (Fut3; 4950-GT-020), and *H. pylori* α1,3 fucosyltransferase (HpFT; 11072-GT-020) were obtained from R&D Systems. GT reaction was performed by mixing 2.5 μl of 10× reaction buffer [25 mM tris-HCl (pH 7.5), 10 mM MnCl_2_], 1.25 μl of 10 mM GDP-fucose, 2.5 μl of GT (Fut2, Fut3, or HpFT; 1.25 μg), and 5 μl of heat-killed cells. The reaction was incubated for 2.5 hours at 37°C. To detect the α1,2-linked fucose residue, Fut2-treated samples were serially diluted with 1× PBS, and 2 μl of each dilution was spotted onto a nitrocellulose membrane. After air drying, the membrane was blocked with 1× Carbo-Free blocking solution (Vector Laboratories, SP-5040-125) for 30 min. Blood group H type 2 antibodies (Thermo Fisher Scientific, MA1-35386) were added at a 1:1000 dilution, and the blot was incubated overnight at 4°C with shaking. The blot was washed twice with PBST and incubated for an hour at room temperature after adding Alexa Fluor 647–conjugated donkey anti-mouse immunoglobulin G (IgG) antibody (Thermo Fisher Scientific, A-31571) at a 1:10,000 dilution. Blots were washed again three times with PBST and imaged. To detect the α-1,3–linked fucose residue, samples treated with Fut3 and HpFT were serially diluted with 1× PBS, and 2 μl of each dilution was spotted onto a nitrocellulose membrane. After air drying, the membrane was blocked with 1× Carbo-Free blocking solution (Vector Laboratories, SP-5040-125) for 30 min. Lectins Lotus Teragonolobus lectin (LTL; Vector Laboratories, FL-1321) and ECL were added at a 1:2500 and 1:5000 dilution, respectively. The blots were incubated for 1 hour at room temperature and imaged.

### Epithelial cell adhesion assays

A549 cells (American Type Culture Collection, CCL-185) were cultured in Dulbecco’s modified Eagle’s medium (DMEM; Biowest, L0104-500) supplemented with 10% (v/v) fetal bovine serum (FBS) (Biowest; S1810-500) at 37°C in 5% CO_2_. Cells were detached from the bottom of the culture bottle by washing them with 10 ml of 1× PBS (Vivantis, PB0344-1L) and incubated with 3 ml of 1× trypsin-EDTA solution diluted in 1× PBS (Biowest, X0930-100) for 5 min at 37°C in 5% CO_2_. Trypsin was removed after adding 10 ml of DMEM and centrifugation at 400*g* for 3 min at room temperature. Cells were resuspended in 16 ml of DMEM, and the cell density and viability were measured by a trypan blue exclusion assay (*59*) using an automated cell counter (Bio-Rad; TC-20). A549 cells were seeded on 24-well plates by adding a suspension containing 2.5 × 10^5^ cells. The plates were centrifuged at 300*g* for 3 min at room temperature to facilitate adhesion, and cells were incubated at 37°C in 5% CO_2_ overnight. *S. pneumoniae* cultures were grown in BHI broth to an OD_600_ of 0.1 to 0.3. Bacterial cells were collected by centrifugation and resuspended to an OD_600_ of 0.1 in DMEM. Bacterial infection was done at a multiplicity of infection (MOI) of 10 (bacteria:A549 cells). The plates were centrifuged at 250*g* for 5 min at room temperature. The coculture was incubated at 37°C in 5% CO_2_ for 3 hours. After incubation, the supernatant with the unbound bacteria was collected. The A549 cells were then washed thrice with 500 μl of 1× PBS. They were detached from the wells by adding 300 μl of 1× trypsin-EDTA solution and incubated at 37°C in 5% CO_2_ for 3 min along with the bound bacteria. Trypsinization was quenched by adding 300 μl of DMEM with 10% FBS. Both the bound and unbound bacteria were diluted with 1× PBS and plated on blood agar. Plates were incubated at 37°C in 5% CO_2_ overnight before enumerating the CFUs.

## Supplementary Materials

### This PDF file includes:

Figs. S1 to S21 Tables S1 to S12 References
