# Supplementary material for: Rewiring the pneumococcal capsule pathway for investigating glycosyltransferase specificity and genetic glycoengineering
Source: Sci Adv. 2023 Sep 6;9(36):eadi8157. doi: 10.1126/sciadv.adi8157 (PMC10482335; doi:10.1126/sciadv.adi8157)
Supplement: Supplementary file 1 — Figs. S1 to S21 Tables S1 to S12 References [file sciadv.adi8157_sm.pdf]

Science Advances
AAAS

Supplementary Materials for

Rewiring the pneumococcal capsule pathway for investigating glycosyltransferase specificity and genetic glycoengineering

Tong Su *et al.*

Corresponding author: Lok-To Sham, lsham@nus.edu.sg

Sci. Adv. **9**, eadi8157 (2023)
DOI: 10.1126/sciadv.adi8157

This PDF file includes:

Figs. S1 to S21
Tables S1 to S12
References

SUPPLEMENTAL FIGURES

Note: For simplicity, only the first four steps are shown and acetylations are omitted

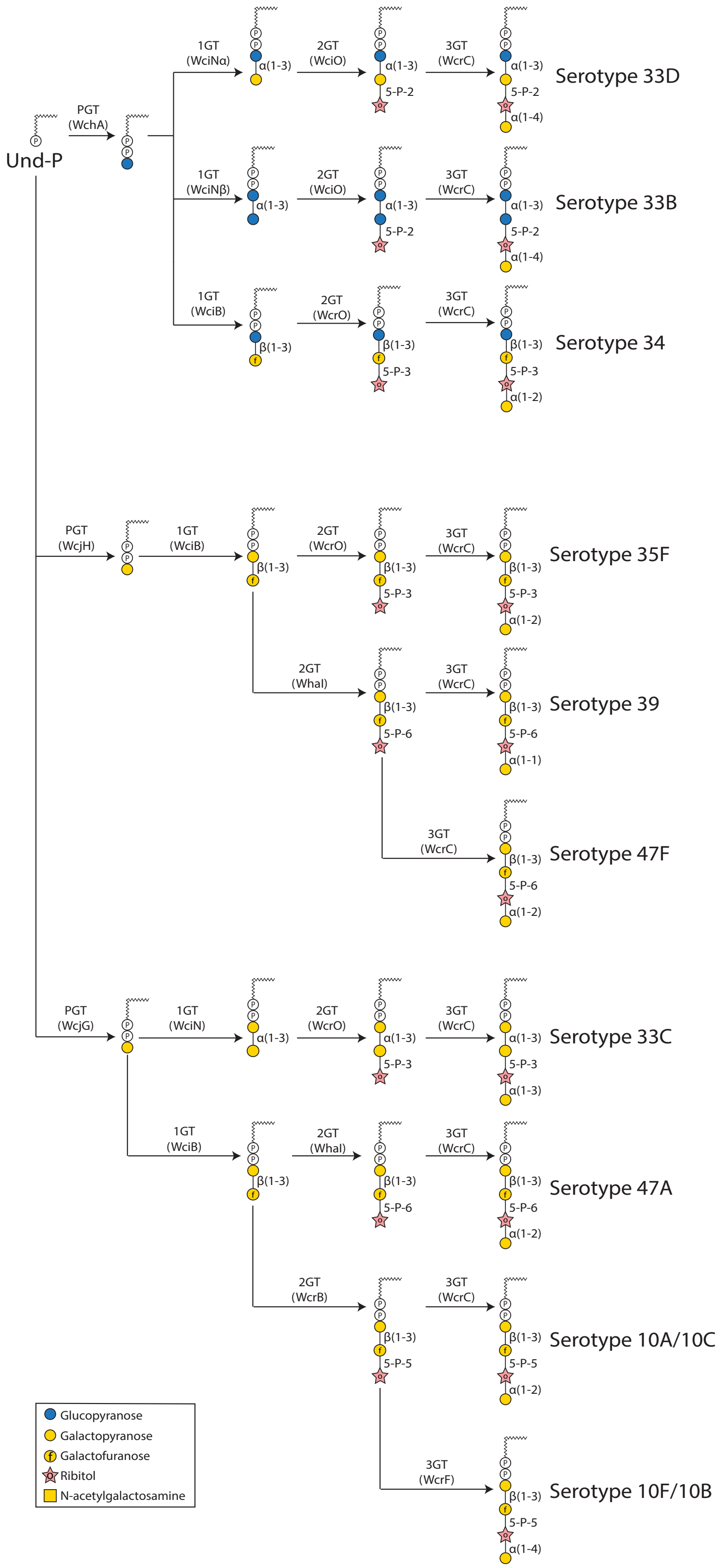

Fig. S1. Synthesis of the serotype 39 and related capsules. Depicted are the first four steps of the indicated CPS synthesis pathways. For simplicity, modifications of the glycan chain such as acetylation are not shown. Note that the enzymes that add ribitol phosphate to the second glycan residue can have different names (WcrB, WhaI, and WcrO) under the current nomenclature system (*60*) (**Table S1**). As they catalyze the formation of the second linkage, we referred to them as 2GTs. Here, we assume the branch chains are added after the main glycan chain is formed. When there are multiple branch chains, we start from the reducing ends to the non-reducing ends (see **Fig. S14**). If the two branches are on the same residue, we arbitrarily assign the GTs in the branches on carbon 1 first and move forward.

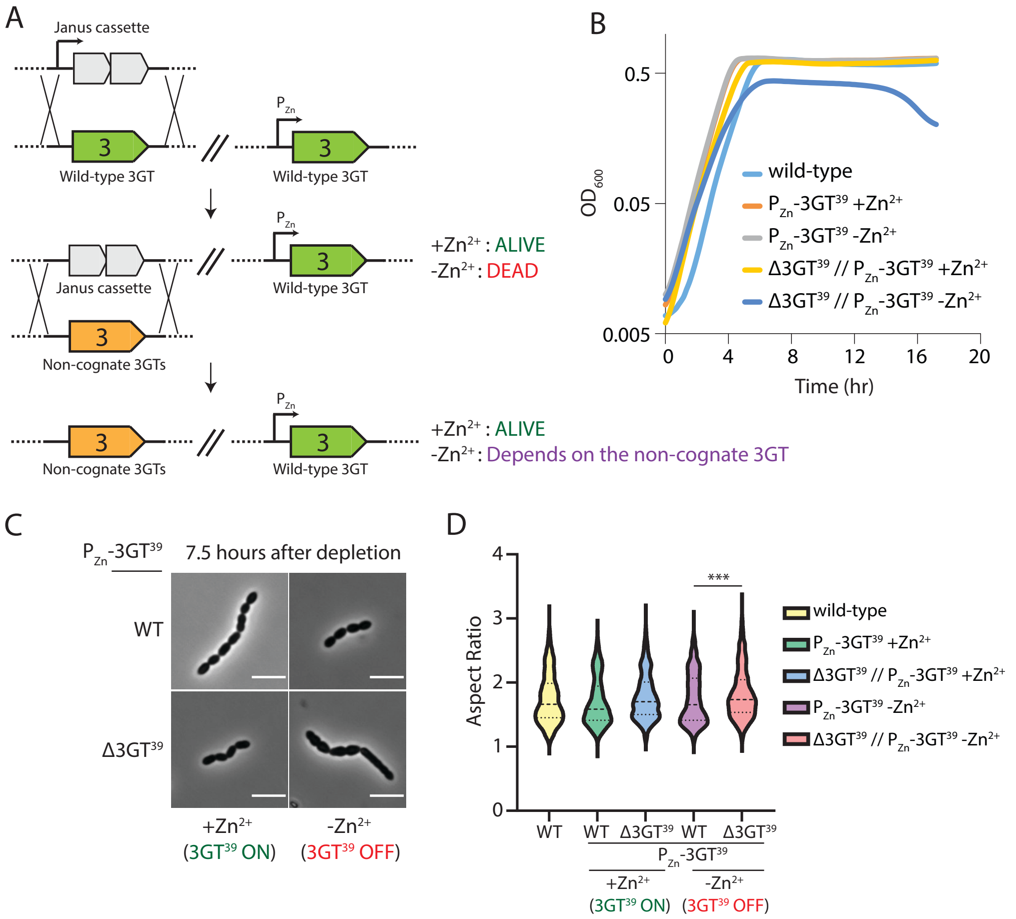

Fig. S2. 3GT^{39} is essential. (**A**) To produce 3GT-switch mutants, the native copy of 3GT^{39} was replaced with the Janus cassette (P-*kan-rpsL*$^{+}$) in a merodiploid background (i.e., strain NUS1223; P$_{Zn}$-3GT^{39}). Allele exchange was performed by substituting the Janus cassette with the non-cognate 3GTs. The resulting strain might be dependent on the Zn^{2+} inducer for growth, depending on the functionality of the non-cognate 3GTs at the native locus. (**B**) Strains NUS0253 [WT], NUS1223 [P$_{Zn}$-3GT^{39}], and NUS1487 [Δ3GT^{39} // P$_{Zn}$-3GT^{39}] were grown in BHI broth with Zn^{2+} supplement overnight at 37°C in 5% CO_2. Cells were harvested by centrifugation, washed once with BHI broth, and diluted in BHI broth with or without Zn^{2+} supplement as indicated. Growth was monitored by measuring optical densities over time. (**C**) Cells of the strains listed in **B** were collected 7.5 hours after 3GT^{39} depletion and visualized under phase contrast microscopy. Shown are representative images from three biological replicates. Cell dimensions were quantitated by MicrobeJ and plotted in (**D**). *P*-values were calculated using the Mann-Whitney U test. ***; $p<0.005$.

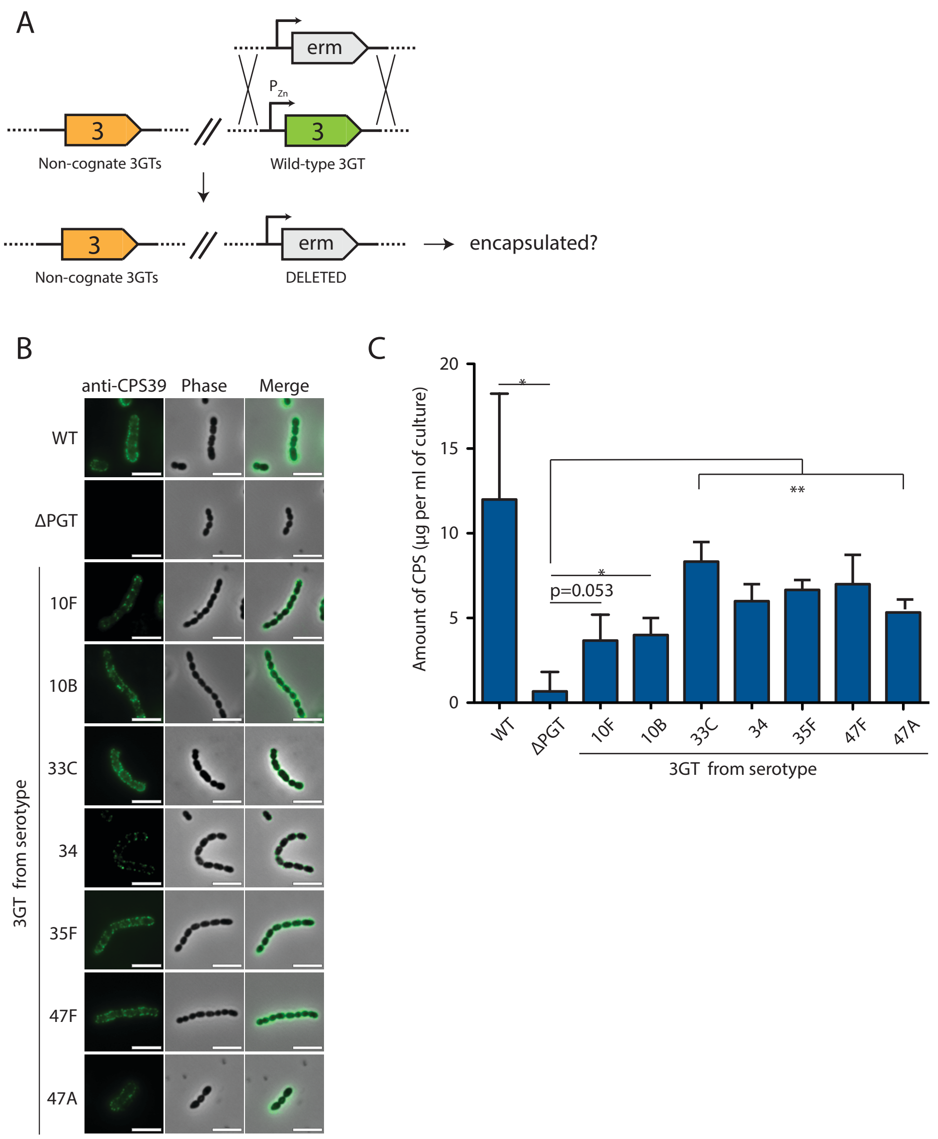

Fig. S3. The 3GT-switch mutants maintain capsule production. (**A**) To prevent leaky expression of the ectopic copy of $3GT^{39}$ (P_{Zn}-$3GT^{39}$), it was replaced by a P-*erm* cassette. The resulting strains had the non-cognate enzyme as the sole copy of 3GT. (**B**) Strains NUS0253 [WT], NUS1365 [ΔPGT], NUS1415 [$3GT^{10F}$], NUS1416 [$3GT^{10B}$], NUS1409 [$3GT^{33C}$], NUS1429 [$3GT^{34}$], NUS1465 [$3GT^{35F}$], NUS1466 [$3GT^{47F}$], and NUS1410 [$3GT^{47A}$] were grown in BHI broth to the mid-log phase. Cells were collected by centrifugation, washed, and stained with the anti-serotype 39 antisera as described in Materials and Methods. Shown are representative images from two biological replicates. (**C**) Quantification of CPS by ELISA. Shown are the means and standard derivations from three biological replicates. *P*-values were computed using two-tailed Student's t-tests. *, $p<0.05$; **, $p<0.01$.

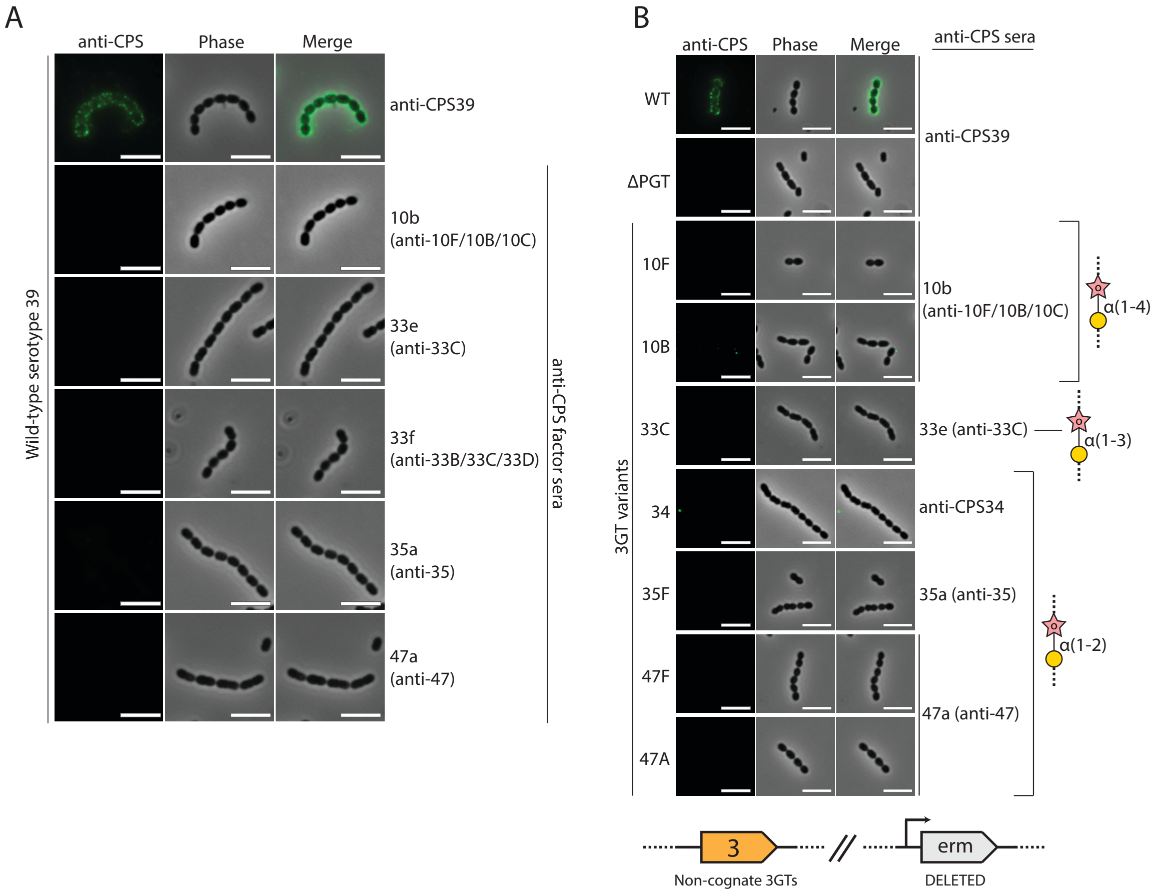

Fig. S4. Changing the third glycosidic linkage in the serotype 39 CPS does not change its serological property. (**A**) The serotype 39 CPS did not react with factor sera 10b, 33e, 33f, 35a, and 47a. Cells of strain NUS0253 [wild type; isogenic serotype 39 capsule-switch mutant] were grown and labeled with the anti-serotype 39 CPS antisera and the indicated factor sera, followed by detection by Alexa Fluor 488 conjugated secondary antibodies. Shown are representative images from two biological replicates. Bar, 4μm. (**B**) Similarly, strains NUS0253, NUS1365 [ΔPGT], NUS1415 [$3GT^{10F}$], NUS1416 [$3GT^{10B}$], NUS1409 [$3GT^{33C}$], NUS1429 [$3GT^{34}$], NUS1465 [$3GT^{35F}$], NUS1466 [$3GT^{47F}$], and NUS1410 [$3GT^{47A}$] were immunostained with the indicated anti-sera that label the CPS types where the 3GTs are originated. Shown are representative images from two biological replicates. Bar, 4μm.

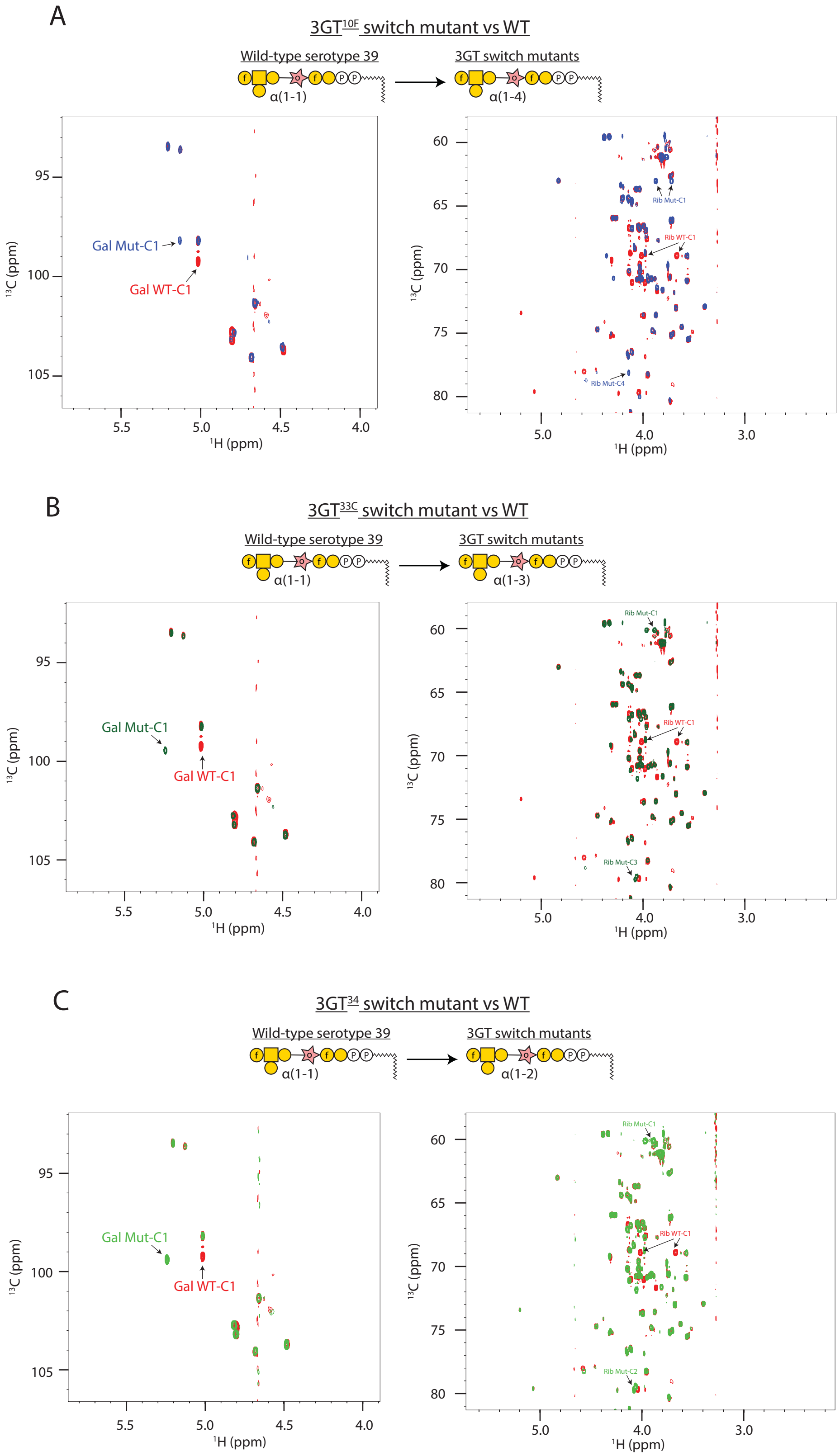

Fig. S5. NMR experiments confirmed the changes in the glycosidic linkages. Heteronuclear Single Quantum Coherence (HSQC) spectra of (**A**) NUS1415 [3GT^{10F} switch mutant], (**B**) NUS1409 [3GT^{33C} switch mutant], and (**C**) NUS1429 [3GT^{34} switch mutant]. Shown are the

relevant regions with the corresponding downfield and upfield. Position 1 of the corresponding sugar residue (Gal, galactose; Rib, ribitol) is denoted as C1, Position 2 as C2, etc. The HSQC spectrum of the isogenic serotype 39 strain (in red; NUS0214; WT) is overlain for comparison.

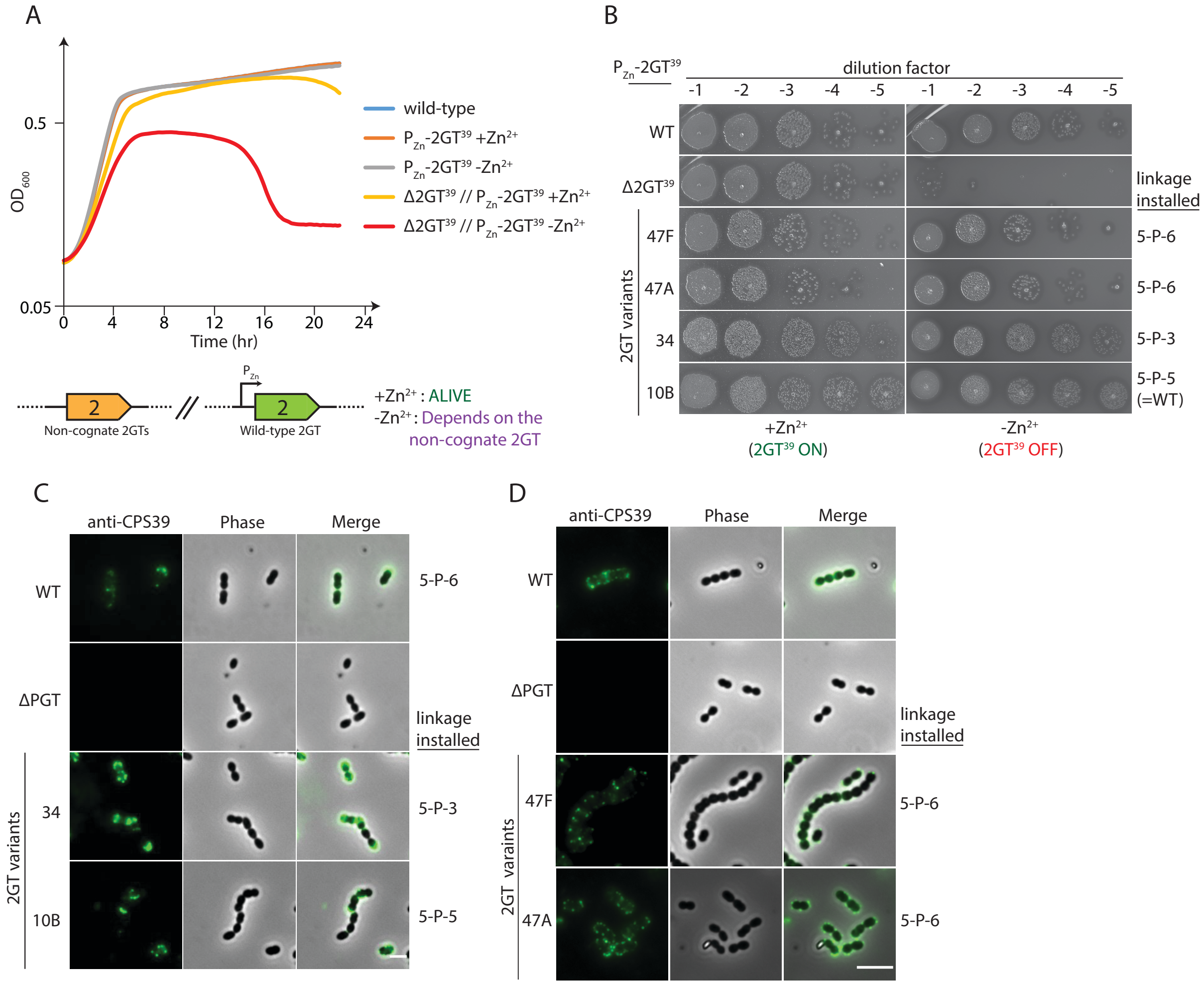

Fig. S6. 2GT^{39} is essential. (**A**) Strains NUS0253 [WT], NUS1430 [P_{Zn}-2GT^{39}], and NUS1463 [Δ2GT^{39} // P_{Zn}-2GT^{39}] were grown in BHI medium with or without Zn^{2+} supplement with their growth monitored. (**B**) The second glycosidic linkage of serotype 39 CPS could be modified. Cultures of strains NUS1430, NUS1463, and the derivatives of NUS1463 carrying the indicated 2GT variants were serially diluted and spotted on blood agar plates with or without Zn^{2+} supplementation. Plates were incubated at 37°C in 5% CO_2 overnight before being photographed. Shown are representative images from three biological replicates. Immunofluorescent microscopy confirmed capsule production in strains harboring 2GT^{34} and 2GT^{10B} (**C**), and 2GT^{47F} and 2GT^{47A} (**D**). Strains NUS0253 [WT], NUS1365 [ΔPGT], NUS2153 [2GT^{34}], NUS2155 [2GT^{10B}], NUS1945 [2GT^{47F}], and NUS1946 [2GT^{47A}] were grown in BHI medium with or without Zn^{2+} supplement until the cultures reached the mid-log phase. Before imaging, cells were heat-killed and labeled with anti-serotype 39 CPS antisera and Alexa Fluor 488 conjugated secondary antibodies.

Shown are representative images from two biological replicates. Bar, 2µm (for **C**) and 4µm (for **D**).

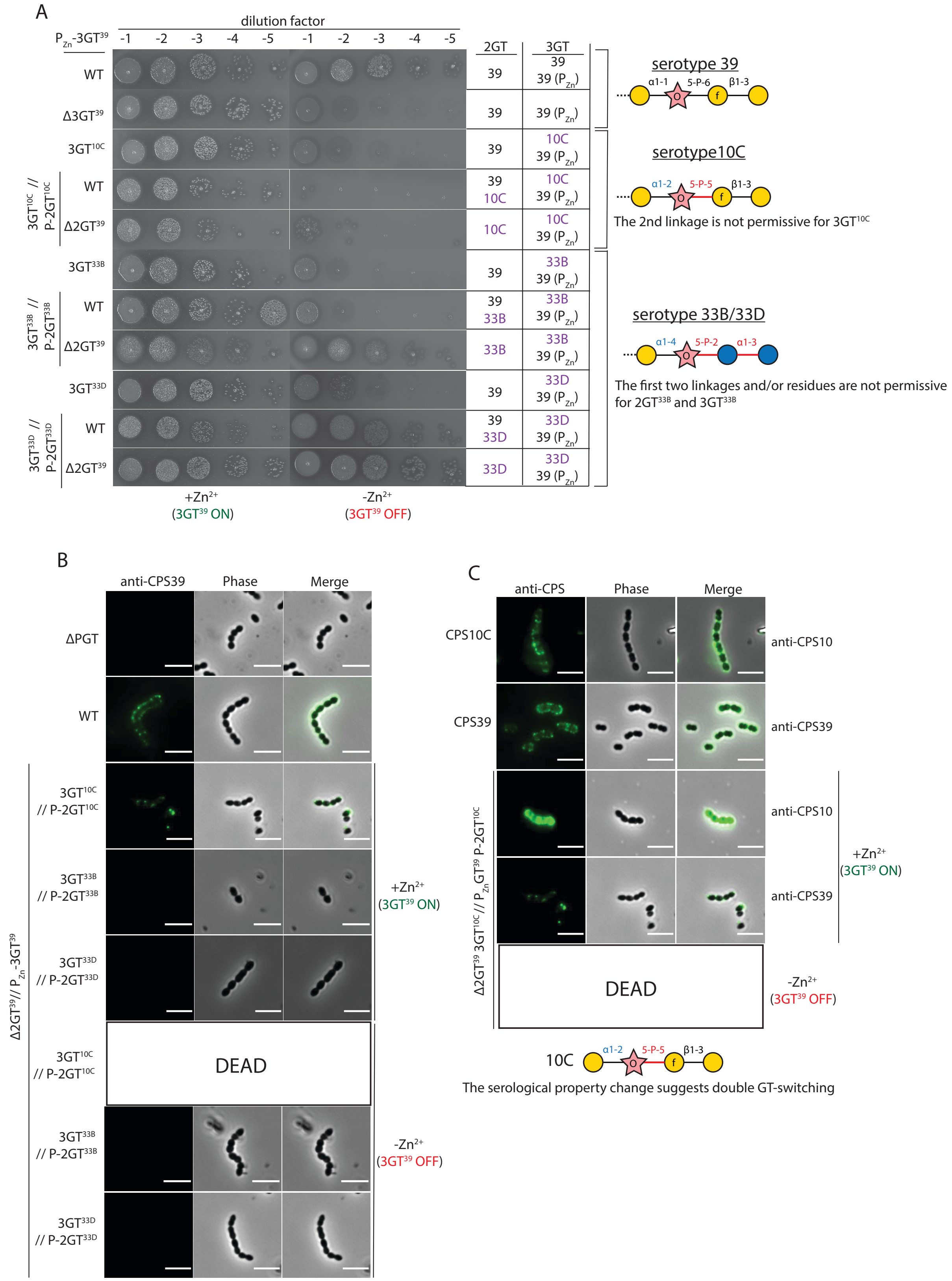

Fig. S7. Changing the second linkage in the serotype 39 CPS to 5-P-5 enables 3GT^{10C} to partially substitute 3GT39. (**A**) Strains NUS1223 [P_{Zn}-3GT^{39}], NUS1303 [Δ3GT^{39} // P_{Zn}-3GT^{39}], NUS1480 [3GT^{10C} // P_{Zn}-3GT^{39}], NUS2015 [3GT^{10C} // P_{Zn}-3GT^{39} // P-2GT^{10C}], NUS2061 [Δ2GT^{39}

$3GT^{10C}$ // P_{Zn}-$3GT^{39}$ // P-$2GT^{10C}$], NUS1355 [$3GT^{33B}$ // P_{Zn}-$3GT^{39}$], NUS2017 [$3GT^{33B}$ // P_{Zn}-$3GT^{39}$ // P-$2GT^{33B}$], NUS2060 [Δ$2GT^{39}$ $3GT^{33B}$ // P_{Zn}-$3GT^{39}$ // P-$2GT^{33B}$], NUS1357 [$3GT^{33D}$ // P_{Zn}-$3GT^{39}$], NUS1555 [$3GT^{33C}$ // P_{Zn}-$3GT^{39}$ // P-$2GT^{33C}$], NUS1645 [Δ$2GT^{39}$ $3GT^{33C}$ // P_{Zn}-$3GT^{39}$ // P-$2GT^{33C}$] were grown in BHI medium with Zn^{2+} supplement to the mid-log phase. Cells were washed with BHI without Zn^{2+}, serially diluted, and spotted on blood agar plates with or without the Zn^{2+} supplement. Plates were incubated overnight at 37°C in 5% CO_2 before imaging. Shown are representative images from three biological replicates. The 2GTs and the 3GTs in the corresponding strains are shown on the right, with the expected glycan structures illustrated after the modification. (**B**) Unlike $2GT^{10C}$, double GT-switch mutants harboring $2GT^{33B}$ and $2GT^{33D}$ lost the capsule and thus likely accumulated suppressor mutations. Strains NUS1365 [ΔPGT], NUS0214 [WT], NUS2061 [Δ$2GT^{39}$ $3GT^{10C}$ // P_{Zn}-$3GT^{39}$ // P-$2GT^{10C}$], NUS2060 [Δ$2GT^{39}$ $3GT^{33B}$ // P_{Zn}-$3GT^{39}$ // P-$2GT^{33B}$], and NUS1645 [Δ$2GT^{39}$ $3GT^{33D}$ // P_{Zn}-$3GT^{39}$ // P-$2GT^{33D}$] were grown in BHI medium with or without Zn^{2+} supplement, heat-killed, and the capsule was immunolabeled. Shown are representative images from two biological replicates. Bar, 4µm. (**C**) Although $3GT^{10C}$ is insufficient to support growth, the change in the capsule serological property suggests it is partially functional in serotype 39. Strains NUS0215 [isogenic serotype 10C mutant; CPS10C], NUS0214 [isogenic serotype 39 mutant; CPS39], and NUS2061 [Δ$2GT^{39}$ $3GT^{10C}$ // P_{Zn}-$3GT^{39}$ // P-$2GT^{10C}$] were grown in BHI medium with or without Zn^{2+} supplement, heat-killed, and the capsule was immunolabeled. Shown are representative images from two biological replicates. Bar, 4µm.

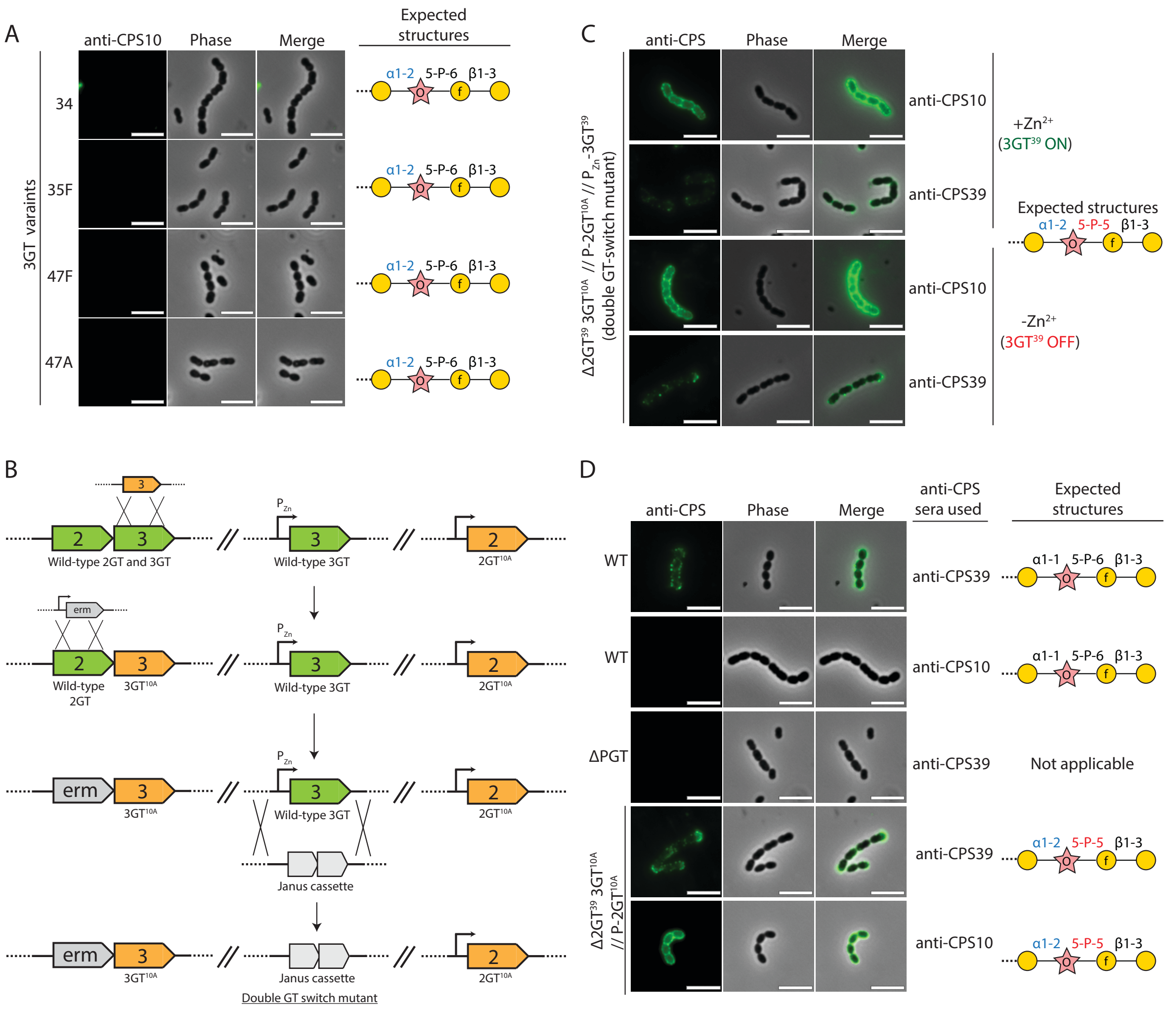

Fig. S8. Double GT-switching results in a CPS that reacts with both anti-serotype 10 and anti-serotype 39 antisera. (**A**) Changing the third glycosidic linkage to α1-2 did not lead to cross-reactivity with anti-serotype 10 antisera. Strains NUS1429 [3GT^{34}], NUS1465 [3GT^{35F}], NUS1466 [3GT^{47F}], and NUS1410 [3GT^{47A}] were grown to the mid-log phase. Cells were stained with anti-serotype 10 antisera and Alexa Fluor 488 conjugated secondary antibodies before imaging. Shown are representative images from two biological replicates. Bar, 4µm. (**B**) Construction of the double GT-switch mutant. 3GT^{39} at the native locus was first replaced by 3GT^{10A}. To support growth, 2GT^{39} was then deleted and 3GT^{39} was supplied by the P$_{Zn}$-3GT^{39} cassette at the ectopic locus. Finally, the P$_{Zn}$-3GT^{39} cassette was deleted, leaving 2GT^{10A} and 3GT^{10A} as the only GTs catalyzing the second and third reactions of the CPS pathway. (**C**) Changing the second and third glycosidic linkages to 5-P-5 and α1-2 led to cross-reactivity with the anti-serogroup 10 antisera. Strain

NUS1644 [$\Delta 2GT^{39}$ $3GT^{10A}$ // P_{Zn}-$3GT^{39}$ // P-$2GT^{10A}$] was labeled with the anti-serogroup 10 or anti-serotype 39 antisera, followed by detection using Alexa Fluor 488 conjugated secondary antibodies. Shown are representative images from two biological replicates. Shown are representative images from two biological replicates. Bar, 4µm. (**D**) Inactivating the ectopic P_{Zn}-$3GT^{39}$ cassette did not affect capsule production in strain NUS1644. Strain NUS1776 [$2GT^{10A}$ $3GT^{10A}$] was grown and labeled with the anti-serogroup 10 or anti-serotype 39 antisera as described in (**C**). Shown on the right are the expected CPS structures of that strain. The modified linkages are highlighted in blue (for 3GTs) or red (for 2GTs), respectively. Bar, 4µm.

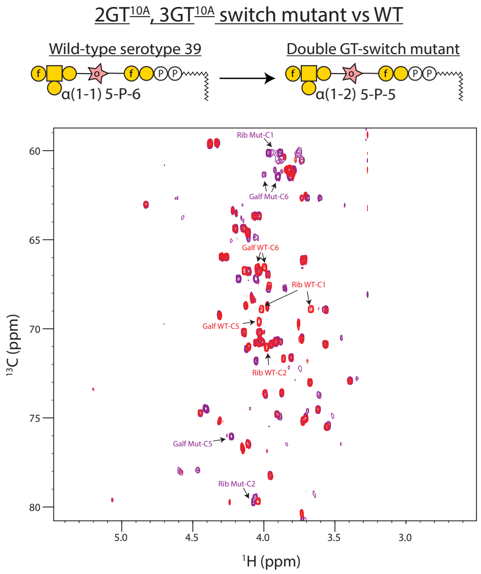

Fig. S9. Validation of the changes in the glycosidic linkages by 2D NMR. Heteronuclear Single Quantum Coherence **(**HSQC) spectra of the CPS purified from strain NUS1776 [2GT^{10A} 3GT^{10A} double GT-switch mutant]. Shown are the relevant regions with the corresponding downfield and upfield. Position 1 of the sugar residue (Gal, galactose; Rib, ribitol) is denoted as C1, and position 2 as C2, etc. The HSQC spectrum of the isogenic serotype 39 strain (in red; NUS0214; WT) is overlain for comparison.

Alignment of the acceptor domains of the 3GTs that installs an α(1-2) glycosidic linkage on ribitol

```
3GT39       -------MKKIALVKWIIDGTDGGLKVSTDLANELSKVYEVHLISVIS-TEEIFFPLNNA    52
3GT10A      -------MKKIALVKWILDDSGGGERVAVSLANELTKKYEVHLIGITTKQSDLFFGINSQ    53
3GT10C      -------MKKIALVKWILDDSGGGERVAVSLANELTKKYEVHLIGITTKQSDLFFGINSQ    53
3GT47A      -------MKKIALVKWILNDSGGGERVAISLANELTKKYEVHLIGITTKQSDLFFKINSQ    53
3GT47F      -------MKKIALVKWILNDSGGGERVAISLANELTKKYEVHLIGITTKQSDLFFKINSQ    53
3GT34       VKGRIEIGKKICIVKWILDDSGGGERVAVSLANELTKKYEVHLIGITTKQSDLFFKINSQ    60
3GT35F      ------LGKKICIVKWILDDSGGGERVAVSLANELTKKYEVHLIGITTKQSDLFFKINSQ    54
                    ***.:****::.:.** :*: .*****:* ******.: :  .::** :*.

3GT39       VRYKNLSSQKISMSKNFVKAVKLLRTYIKKNNIEVLFGIGMTMNIVGITSTIGLKTKFIS    112
3GT10A      VKYSNFFDHRVRLSKNILKISKMLKKYFLDNEIEVAFGIGIFSNIFLSLSGIGISTKVVL    113
3GT10C      VKYSNFFDHRVRLSKNILKISKMLKKYFLDNEIEVAFGIGIFANVFLSLSGIGISTKVVL    113
3GT47A      VKYSNFFDHRVRLSKNLLKISKMLKNYFVDNEIDVAFGIGISANIFLSLAGIGISTKIVL    113
3GT47F      VKYSNFFDHRVRLSKNLLKISKMLKNYFVDNEIDVAFGIGISANVFLSLAGIGTQTKVVL    113
3GT34       VRYSNFFDHRVRLSKNILKISKMLRKYFIDNEIEVGFGIGISANVFLSLAGIGTQTKVVL    120
3GT35F      VRYSNFFDHRVRLSKNILKISKMLRKYFIDNEIEVGFGIGISANVFLSLAGIGTQTKVVL    114
            *:*.*: .::: :***::*  *:*:.*: .*:*:* ****:  *:.   : ** .**.:

3GT39       CDHTNSIVDIDTTMKKLQRYVGAKLSDKIITLTTADRNNYIKKYKINPKRVDYIYNWMNS    172
3GT10A      CDHTNSITANRELSKKVQRYVGTKLADKIITLTQEDRKNYIRKYGISENRIAYIYNWKEN    173
3GT10C      CDHTNSITANRELSQKVQRYVGTKLADKIITLTQEDRKNYIRKYGISENRIAYIYNWKEN    173
3GT47A      CDHTNSITDNREFSQKIQRYIGTKLADKIITLTQEDCQNYIKKYGIAEGIIDYIYNWKEN    173
3GT47F      CDHTNSITDNREFYQKFQRYVGTKLADKIITLTQEDRENYIKKYGVPEERICYIYNWKEA    173
3GT34       CDHTNSITDNREFSQKFQRYVGTKLADKIITLTQEDRENYIKKYGVPEERICYIY-----    175
3GT35F      CDHTNSITDNREFSQKFQRYVGTKLADKIITLTQEDRENYIKKYGVPEERICYIYNWKEA    174
            *******.      :*.***:*:**:*******  * :***:** :    : ***

3GT39       IDA 175
3GT10A      RL- 175
3GT10C      RL- 175
3GT47A      IP- 175
3GT47F      AL- 175
3GT34       --- 175
3GT35F      A-- 175
```

Unable to complement

Able to complement

Fig. S10. Alignment of acceptor domains of the 3GTs that are related to 3GT[39]. Multiple sequence alignment was performed using Clustal Omega and the amino acid residues of the 3GT variants that are different from 3GT[39] are highlighted by red boxes. The 3GT variants that could not complement 3GT[39] are shaded in pink, whereas the rest are shaded in blue.

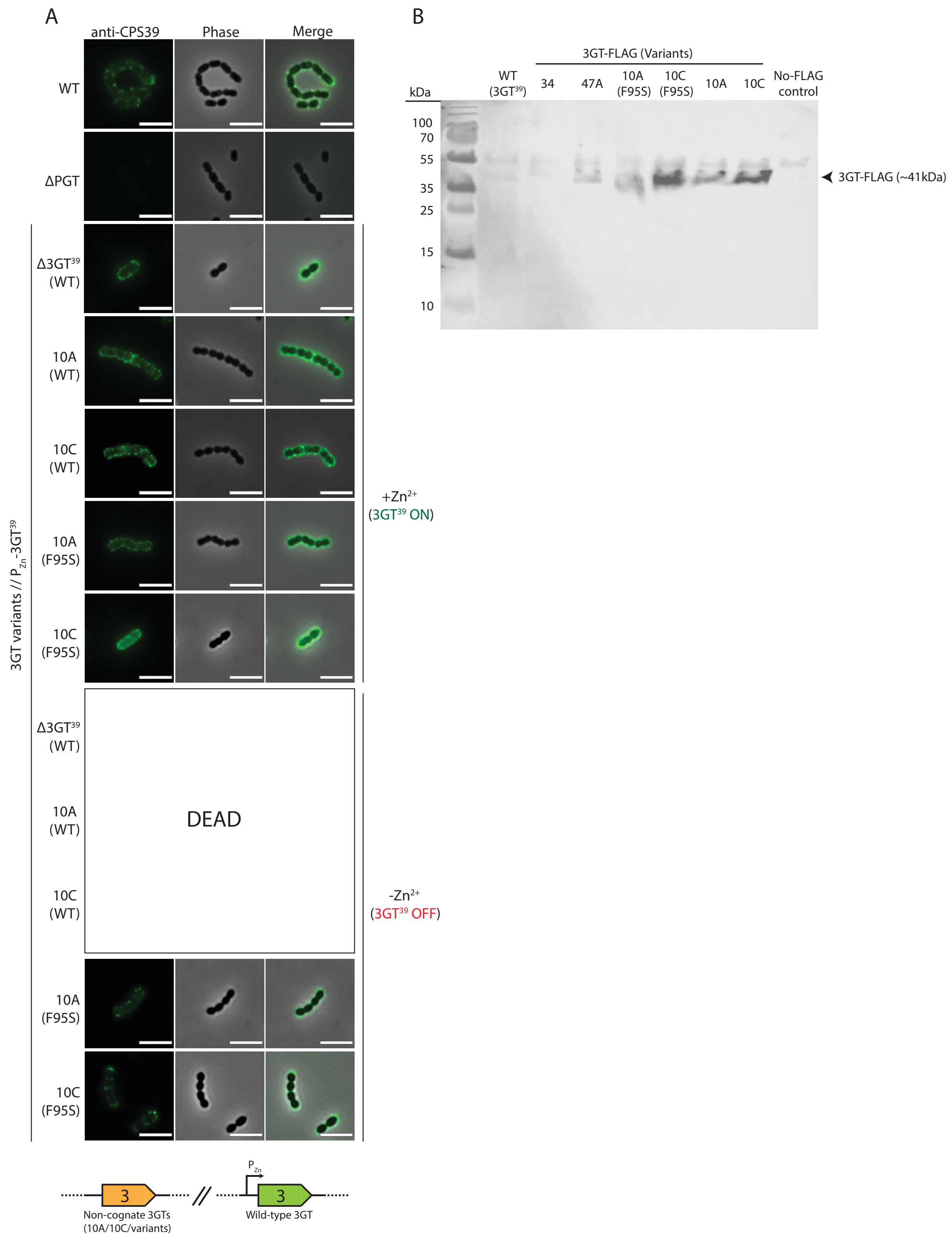

Fig. S11. 3GT^{10A}(F95S) and 3GT^{10C}(F95S) supported capsule production in serotype 39. (A) Strains NUS0214 [WT], NUS1365 [ΔPGT], NUS1303 [Δ3GT^{39} // P$_{Zn}$-3GT^{39}], NUS1479 [3GT^{10A} // P$_{Zn}$-3GT^{39}], NUS1480 [3GT^{10C} // P$_{Zn}$-3GT^{39}], NUS1643 [3GT^{10A}(F95S) // P$_{Zn}$-3GT^{39}], and NUS1662 [3GT^{10C}(F95S) // P$_{Zn}$-3GT^{39}] were grown in BHI broth to the mid-log phase with or

without Zn^{2+} addition. Cells were harvested and immunostained with the anti-serotype 39 antisera and Alexa Fluor 488 conjugated secondary antibodies before imaging. Shown are representative images from two biological replicates. Bar, 4µm. (B) Strains NUS2329 [$3GT^{39}$-FLAG // P_{Zn}-$3GT^{39}$], NUS2325 [$3GT^{34}$-FLAG // P_{Zn}-$3GT^{39}$], NUS2327 [$3GT^{47A}$-FLAG // P_{Zn}-$3GT^{39}$], NUS0747 [$3GT^{10A(F95S)}$-FLAG // P_{Zn}-$3GT^{39}$], NUS0748 [$3GT^{10C(F95S)}$-FLAG // P_{Zn}-$3GT^{39}$], NUS3027 [$3GT^{10A}$-FLAG // P_{Zn}-$3GT^{39}$], NUS3028 [$3GT^{10C}$-FLAG // P_{Zn}-$3GT^{39}$], NUS0253 [WT] were grown in BHI broth with or without added Zn^{2+}. When the cultures reached the mid-log phase, cells were collected by centrifugation. Proteins were extracted and the FLAG-tagged proteins were detected by immunoblotting using anti-FLAG antibodies. The expected size of the 3GT-FLAG variants is 41kDa. The non-specific bands above the 3GT-FLAG bands serve as an additional loading control. Shown are the representative blot from two biological replicates.

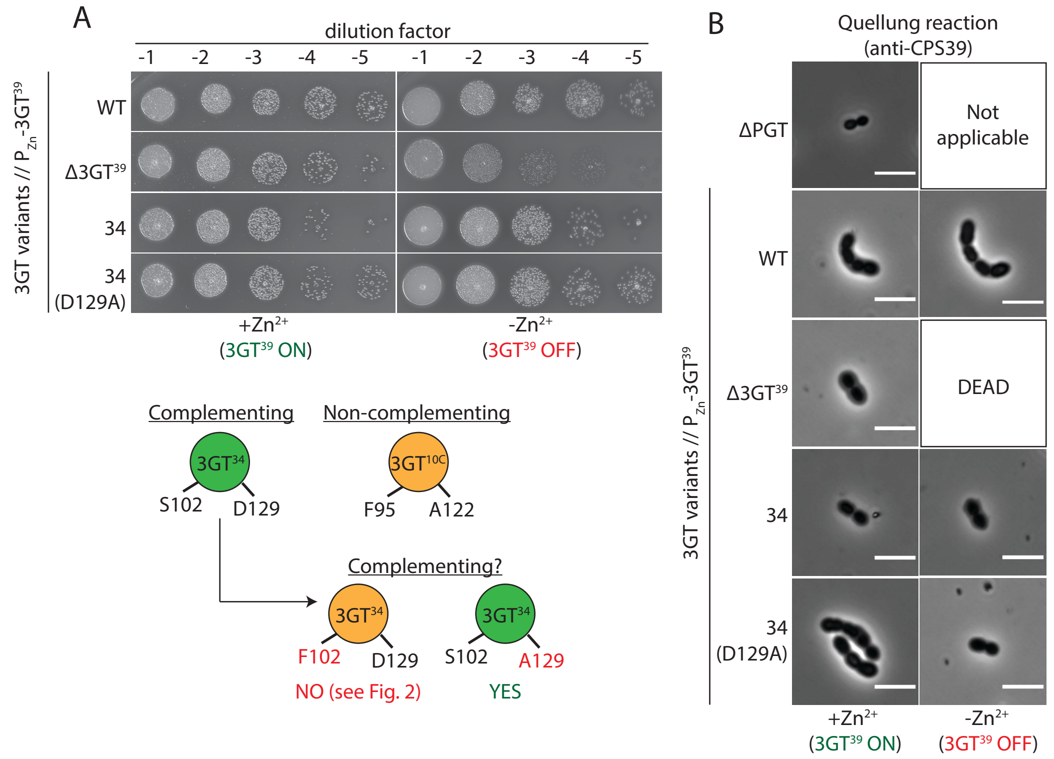

Fig. S12. Residue D129 of 3GT^{34} is not the determinant of regiospecificity for the second glycosidic linkage. (**A**) Strains NUS1223 [P$_{Zn}$-3GT^{39}; WT], NUS1303 [Δ3GT^{39} // P$_{Zn}$-3GT^{39}], NUS1426 [3GT^{34} // P$_{Zn}$-3GT^{39}], and NUS1603 [3GT^{34}(D129A) // P$_{Zn}$-3GT^{39}] were grown in BHI medium with Zn^{2+} supplementation, washed, serially diluted, and spotted on blood agar plates with or without added Zn^{2+}. Plates were incubated overnight at 37°C in 5% CO_2 before imaging. Shown are representative images from three biological replicates. (**B**) Cells of strain NUS1365 [ΔPGT], NUS1223, NUS1303, NUS1426, and NUS1603 were grown in BHI medium to the mid-log phase with or without Zn^{2+} supplementation and stained with anti-serotype 39 antisera. The phase-dense halos surrounding the phase-dark cells are precipitations of the anti-capsule antibodies (i.e., Quellung reactions). Shown are representative images from two biological replicates. Bar, 4µm.

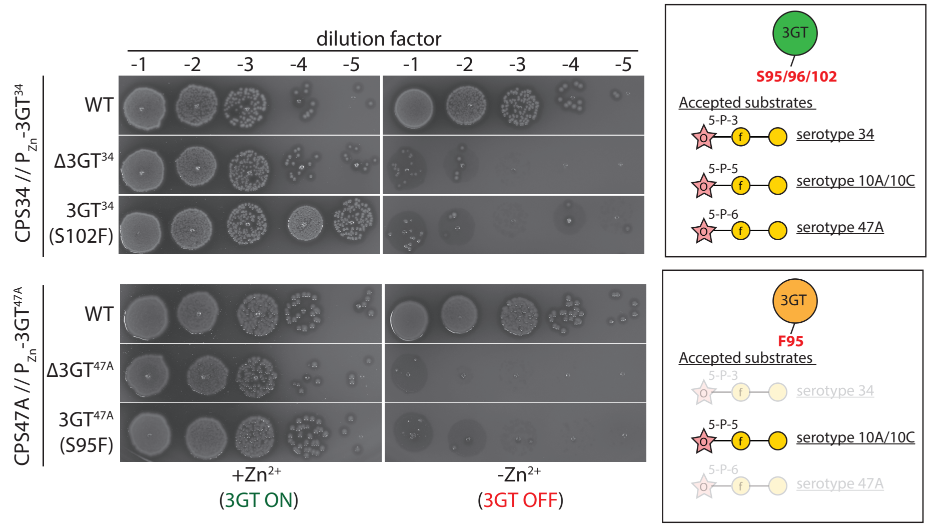

Fig. S13. $3GT^{34}$(S102F) and $3GT^{47A}$(S95F) are unable to use their cognate acceptors because they have a 5-P-3 or 5-P-6 linkage between the ribitol and the galactose residue, respectively. Strains NUS1939 [P_{Zn}-$3GT^{34}$; WT], NUS1969 [$\Delta 3GT^{34}$ // P_{Zn}-$3GT^{34}$], NUS2188 [$3GT^{34}$ (S102F) // P_{Zn}-$3GT^{39}$], NUS1921 [P_{Zn}-$3GT^{47A}$; WT], NUS1970 [$\Delta 3GT^{47A}$ // P_{Zn}-$3GT^{47A}$], NUS2189 [$3GT^{47A}$ (S95F) // P_{Zn}-$3GT^{47A}$] were grown in BHI medium supplemented with Zn^{2+}, serially diluted, and spotted on blood agar plates with or without Zn^{2+} supplementation. Plates were incubated overnight at 37°C in 5% CO_2 before imaging. Shown are representative images from two biological replicates.

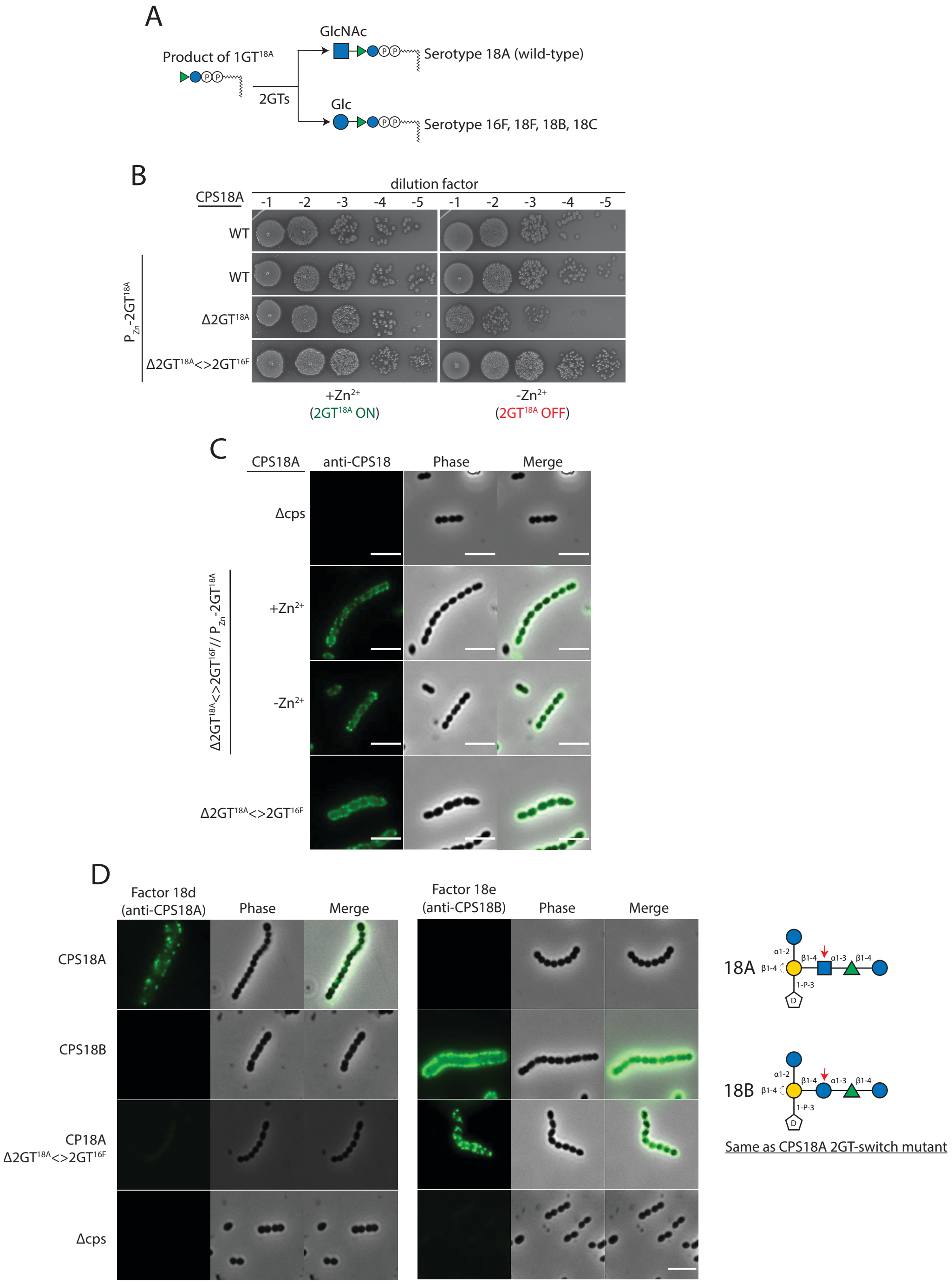

Fig. S14. GT-switching led to the substitution of a sugar residue. (**A**) $2GT^{18A}$ installs a GlcNAc residue to the glycosylated lipid precursor, whereas $2GT^{16F}$ and other 2GTs in serogroup 18 add a Glc residue. (B) (**B**) $2GT^{18A}$ could be substituted by $2GT^{16A}$. Strains NUS0391 [WT], NUS1289 [P_{Zn}-$2GT^{18A}$], NUS1968 [$\Delta 2GT^{18A}$ // P_{Zn}-$2GT^{18A}$], and NUS2342 [$2GT^{18A}$<>$2GT^{16A}$ // P_{Zn}-$2GT^{18A}$] were grown in BHI broth supplement with Zn^{2+}, washed, and resuspended BHI without Zn^{2+}, and spotted on blood agar plates with or without added Zn^{2+} supplement. Plates were incubated

overnight at 37°C in 5% CO_2 before imaging. Shown are representative images from two biological replicates. (**C**) The 2GT-switch mutant remained encapsulated. Strains NUS0114 [Δ*cps*], NUS2342, and NUS2367 [$2GT^{18A}$<>$2GT^{16A}$] were grown in BHI with or without Zn^{2+}supplement and stained with anti-serotype 18 CPS antiserum, followed by detection with fluorescent secondary antibodies. Shown are representative images from two biological replicates. (**D**) The 2GT-switch mutant of the serotype 18A mutant is serologically indistinguishable from serotype 18B. Strains NUS0391 [CPS18A], NUS0330 [CPS18B], NUS2367, and NUS114 were grown in BHI broth to the mid-log phase and immunostained with factor 18d (anti-CPS18A) and factor 18e (anti-CPS18B). Shown are representative images from two biological replicates. Bar, 4μm.

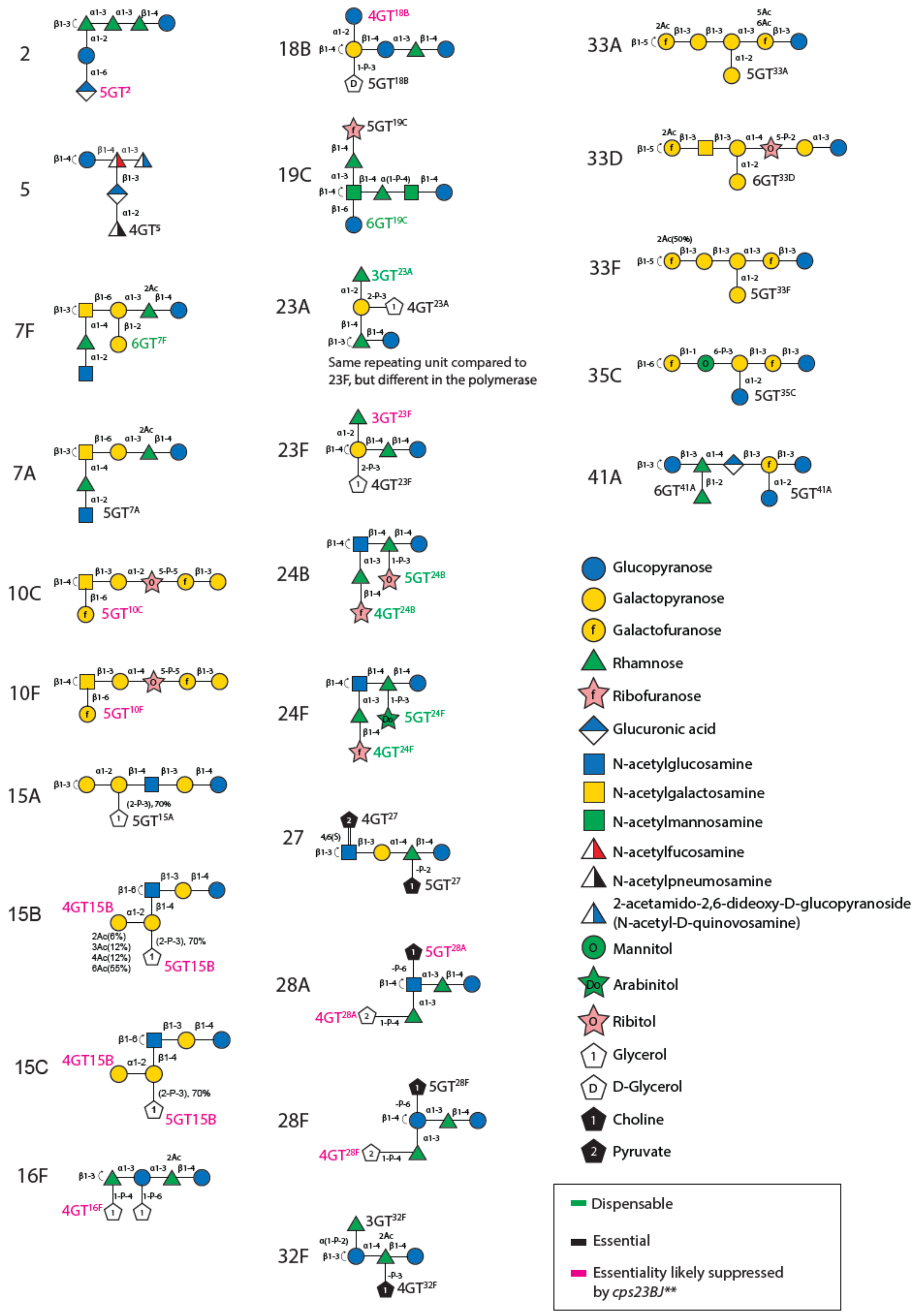
2
5GT2
5
4GT5
7F
6GT7F
7A
5GT7A
10C
5GT10C
10F
5GT10F
15A
5GT15A
15B
4GT15B
5GT15B
15C
4GT15B
5GT15B
16F
4GT16F
18B
4GT18B
5GT18B
19C
5GT19C
6GT19C
23A
3GT23A
4GT23A
Same repeating unit compared to
23F, but different in the polymerase
23F
3GT23F
4GT23F
24B
5GT24B
4GT24B
24F
5GT24F
4GT24F
27
4GT27
5GT27
28A
5GT28A
4GT28A
28F
5GT28F
4GT28F
32F
3GT32F
4GT32F
33A
5GT33A
33D
6GT33D
33F
5GT33F
35C
5GT35C
41A
6GT41A
5GT41A
Glucopyranose
Galactopyranose
Galactofuranose
Rhamnose
Ribofuranose
Glucuronic acid
N-acetylglucosamine
N-acetylgalactosamine
N-acetylmannosamine
N-acetylfucosamine
N-acetylpneumosamine
2-acetamido-2,6-dideoxy-D-glucopyranoside
(N-acetyl-D-quinovosamine)
Mannitol
Arabinitol
Ribitol
Glycerol
D-Glycerol
Choline
Pyruvate
Dispensable
Essential
Essentiality likely suppressed
by cps23BJ**

Fig. S15. Cps23BJ allows deletions of GTs that are essential for growth**. Shown are the CPS structures of the GTs examined in this study. GTs that are dispensable for growth are labeled in green, essential GTs are in black, and those that can be suppressed by Cps23BJ** are in pink.

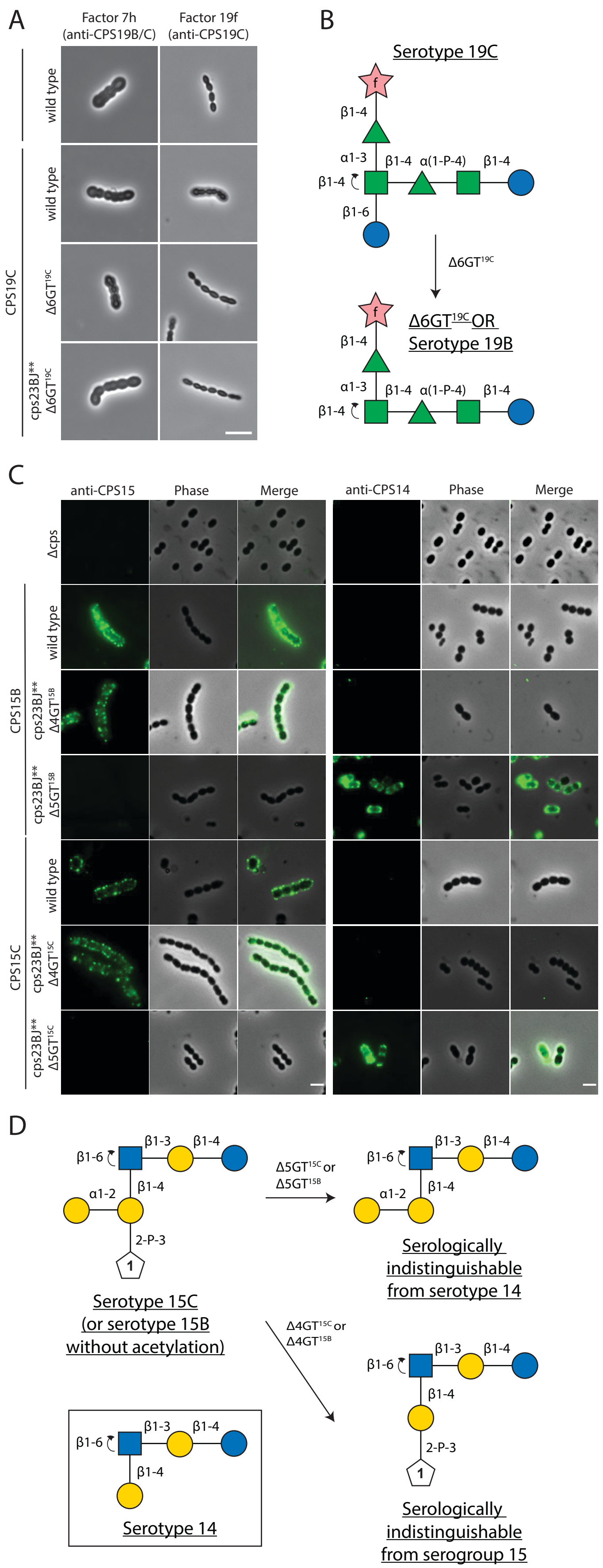
A
Factor 7h (anti-CPS19B/C)
Factor 19f (anti-CPS19C)
wild type
CPS19C
wild type
Δ6GT19C
cps23BJ** Δ6GT19C
B
Serotype 19C
f
β1-4
α1-3
β1-4
α(1-P-4)
β1-4
β1-4
β1-6
Δ6GT19C
Δ6GT19C OR Serotype 19B
C
anti-CPS15
Phase
Merge
anti-CPS14
Phase
Merge
Δcps
CPS15B
wild type
cps23BJ** Δ4GT15B
cps23BJ** Δ5GT15B
CPS15C
wild type
cps23BJ** Δ4GT15C
cps23BJ** Δ5GT15C
D
β1-6
β1-3
β1-4
α1-2
β1-4
2-P-3
1
Serotype 15C (or serotype 15B without acetylation)
Δ5GT15C or Δ5GT15B
Serologically indistinguishable from serotype 14
Δ4GT15C or Δ4GT15B
Serologically indistinguishable from serogroup 15
Serotype 14

Fig. S16. Seroconversion caused by deletions of GTs. (**A**) Strains NUS0317 [isogenic serotype 19B mutant; CPS19B], NUS0316 [isogenic serotype 19C mutant; CPS19C], NUS3253 [CPS19C $\Delta 6GT^{19C}$], and NUS3134 [CPS19C *cps23BJ*** $\Delta 6GT^{19C}$] were grown in BHI medium to the mid-log phase. Cells were harvested and immunostained with factor sera 7h (detect serotype 19B and 19C) and 19f (detect serotype 19C only) (i.e., Quellung reaction). Shown are representative images from two biological replicates. Bar, 5µm. (**B**) Structure of the serotype 19C CPS and its derivative (the $\Delta 6GT^{19C}$ mutant). (**C**) Strain NUS0483 [Δ*cps*], NUS0340 [isogenic serotype 15B mutant; CPS15B], NUS3888 [CPS15B *cps23BJ*** $\Delta 4GT^{15B}$], NUS3889 [CPS15B *cps23BJ*** $\Delta 5GT^{15B}$], NUS0289 [isogenic serotype 15C mutant; CPS15C], NUS3890 [CPS15C *cps23BJ*** $\Delta 4GT^{15C}$], and NUS3917 [CPS15C *cps23BJ*** $\Delta 5GT^{15C}$] were grown in BHI broth to the mid-log phase. Cells were collected and immunostained with anti-serogroup 15 antisera (left) or anti-serotype 14 antisera (right). Shown are representative images from two biological replicates. Bar, 2µm. (**D**) Structure of the serotype 15C CPS and the modifications introduced by deletions of 4GT or 5GT.

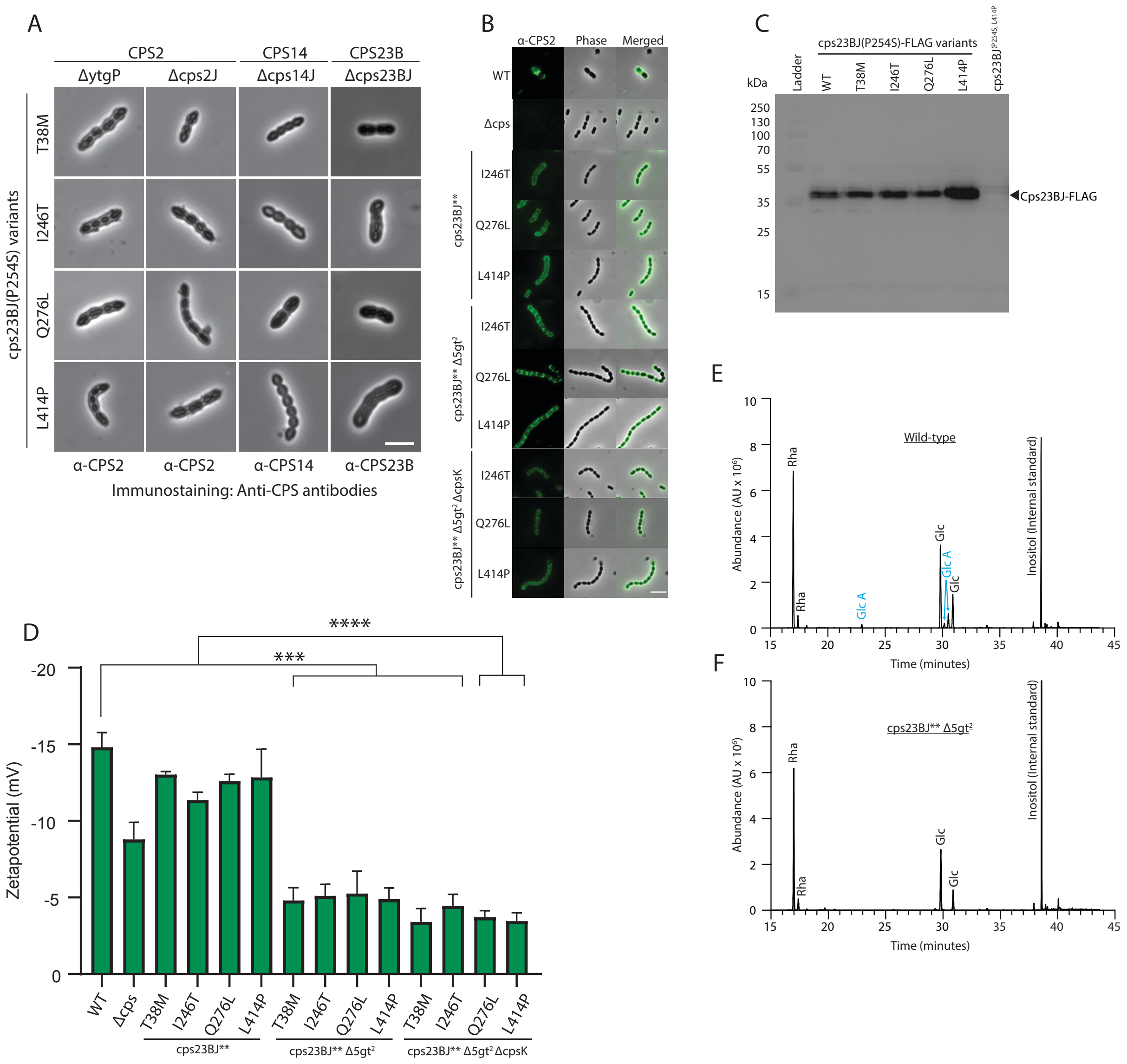

Fig. S17. Characterization of Cps23BJ and 5GT² deletion mutants that lack the terminal GlcUA residue.** (**A**) Cps23BJ** can transport lipid II (the substrate of YtgP) and the lipid-linked precursors of serotypes 2, 14, and 23B. Cps23BJ* (Cps23BJ^{P254S}) was PCR mutagenized to select alleles that could complement YtgP (the lipid II flippase). Strains harboring the indicated *cps23BJ*** alleles and deletions (**Table S11**) were grown in the BHI medium until the mid-log phase. Cells were harvested and stained with the anti-serotype 2, serotype 14, and serogroup 23 antisera. Shown are representative images from three biological replicates. Bar, 4µm. (**B**) Other *cps23BJ*** alleles could also bypass the essentiality of 5GT² and *cpsK*. Strains harboring the indicated *cps23BJ*** alleles and deletions (**Table S11**) were grown in the BHI medium until the mid-log phase. Cells were harvested and immunostained with the anti-serotype 2 antisera and Alexa

Fluor 488 conjugated secondary antibodies. Shown are representative images from two biological replicates. Bar, 4µm. (**C**) Except for L414P, Cps23BJ** variants were not overproduced. Strains NUS3018 [WT], NUS3019 [T38M], NUS3020 [I246T], NUS3021 [Q276L], NUS3022 [L414P], and NUS2866 were grown in the BHI medium until the mid-log phase. Cells were collected by centrifugation and lysed. Proteins were resolved on an SDS-PAGE gel and transferred to a membrane, followed by detection using anti-FLAG antibodies. Shown are representative images from three biological replicates. (**D**) Inactivating *cpsK* in the $5GT^2$ deletion mutant did not further alter the cell surface charge. Strains with the indicated *cps23BJ*** alleles and their derivatives that lack $5GT^2$ and *cpsK* were grown in the BHI medium until the mid-log phase. Cells were harvested by centrifugation, washed, and analyzed using a zetasizer. Shown are the means and standard derivations from three technical replicates. *P*-values were computed with two-tailed Student's t-tests. ***, $p<0.005$; ****, $p<0.001$. (**E**) Carbohydrate composition analysis confirmed the lack of GlcUA in the $5GT^2$ mutant. Strains IU1781 [wild-type] and NUS3000 [*cps23BJ*** $\Delta 5GT^2$] were grown in the BHI medium until the mid-log phase. Cells were harvested, boiled in SDS, and washed in water. The crude sacculi recovered were treated with mutanolysin, lysozyme, and LytA overnight at 37˚C. Enzymes were degraded by proteinase K treatment and heat-inactivated. Insoluble materials were removed by centrifugation and the supernatant fraction containing the released CPS was extracted with phenol-chloroform. The CPS in the aqueous phase was dialyzed and lyophilized, before acid hydrolysis and GC-MS analysis. The mass spectrometry spectrum of the wild-type strain (**E**) confirmed the presence of GlcUA in the serotype 2 CPS (23.0, 30.2, and 30.5 minutes), which was not detected in strain NUS3000 (**F**).

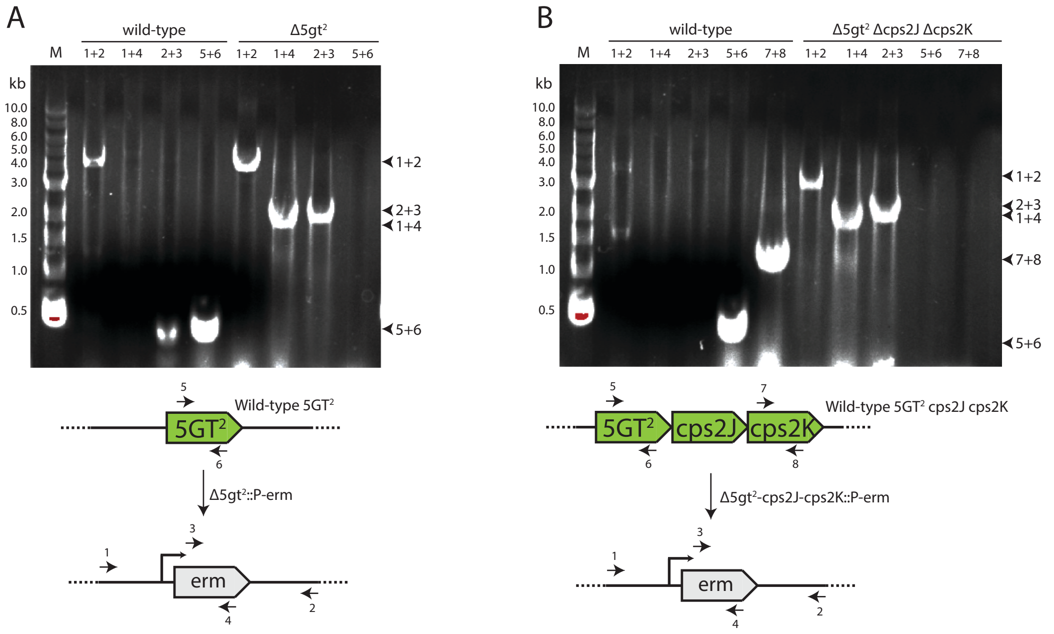

Fig. S18. Validation of the 5GT^2 deletion mutants. (**A**) Cells of strains IU1781 [wild-type] and NUS2987 [Δ5GT^2] were diagnosed by PCR using the indicated primers. (**B**) The same experiment was performed as described in (**A**) except different primers were used for analyzing strains NUS3000 [Δ5GT^2 Δ*cpsJ* Δ*cpsK*].

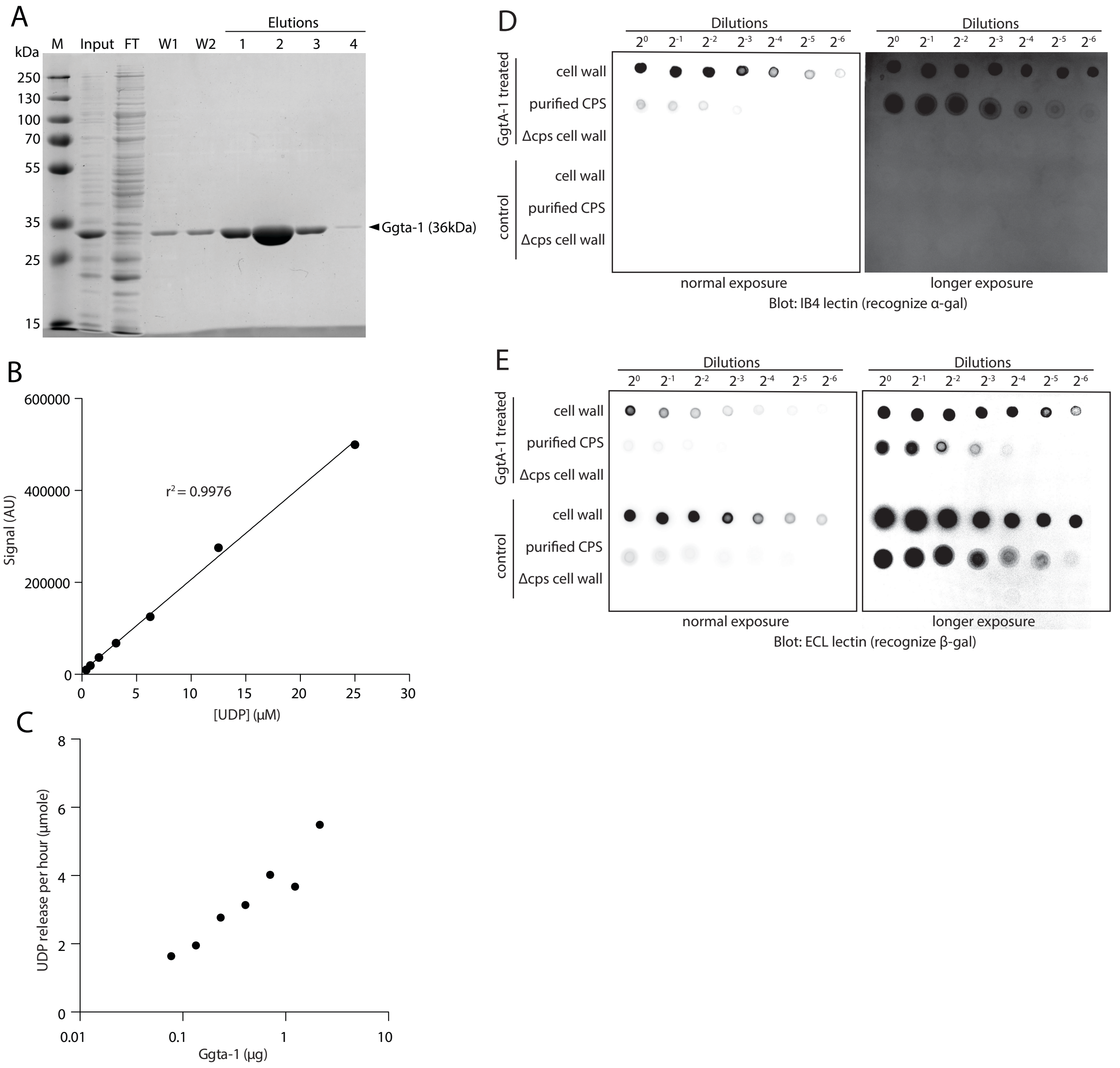

Fig. S19. Biochemical characterization of Ggta-1. (**A**) Purification of Ggta-1. Strain LEMO21(IDE3)/pCS190 [P_{T7}-his_6-*ggta1*Δ74] was grown in the terrific broth and induced at 16°C overnight. Cells were collected and lysed by sonication (input). The lysate was loaded on a TALON metal affinity column and the flow-through fraction was discarded (flow-through; FT). The column was washed twice (W1 and W2) and eluted by buffer with imidazole (Elution 1 to 4). Protein fractions were resolved on an SDS-PAGE and stained with Coomassie blue. UDP release after mixing Ggta-1, UDP-Gal, and the serotype 14 substrate was measured using the UDP-Glo kit. Shown is the standard curve with the internal UDP standard (**B**) and the amount of UDP released per hour concerning the amount of Ggta-1 added to the reaction mix (**C**). Shown are the representative diagrams from two biological replicates. Ggta-1 can utilize purified CPS as the

acceptor. Cells boiled in SDS (cell wall), purified CPS, and the cell wall extracted from an isogenic unencapsulated strain NUS3278 [CPS14 Δ*cpsE*] were treated with Ggta-1 overnight. Samples were serially diluted and spotted on a nitrocellulose membrane. After air drying, the membrane was blotted with fluorescein-labeled IB4 lectin (**D**) or ECL lectin (**E**). The same blot was exposed longer (on the right) to detect weaker signals. Shown are the representative blots from three biological replicates.

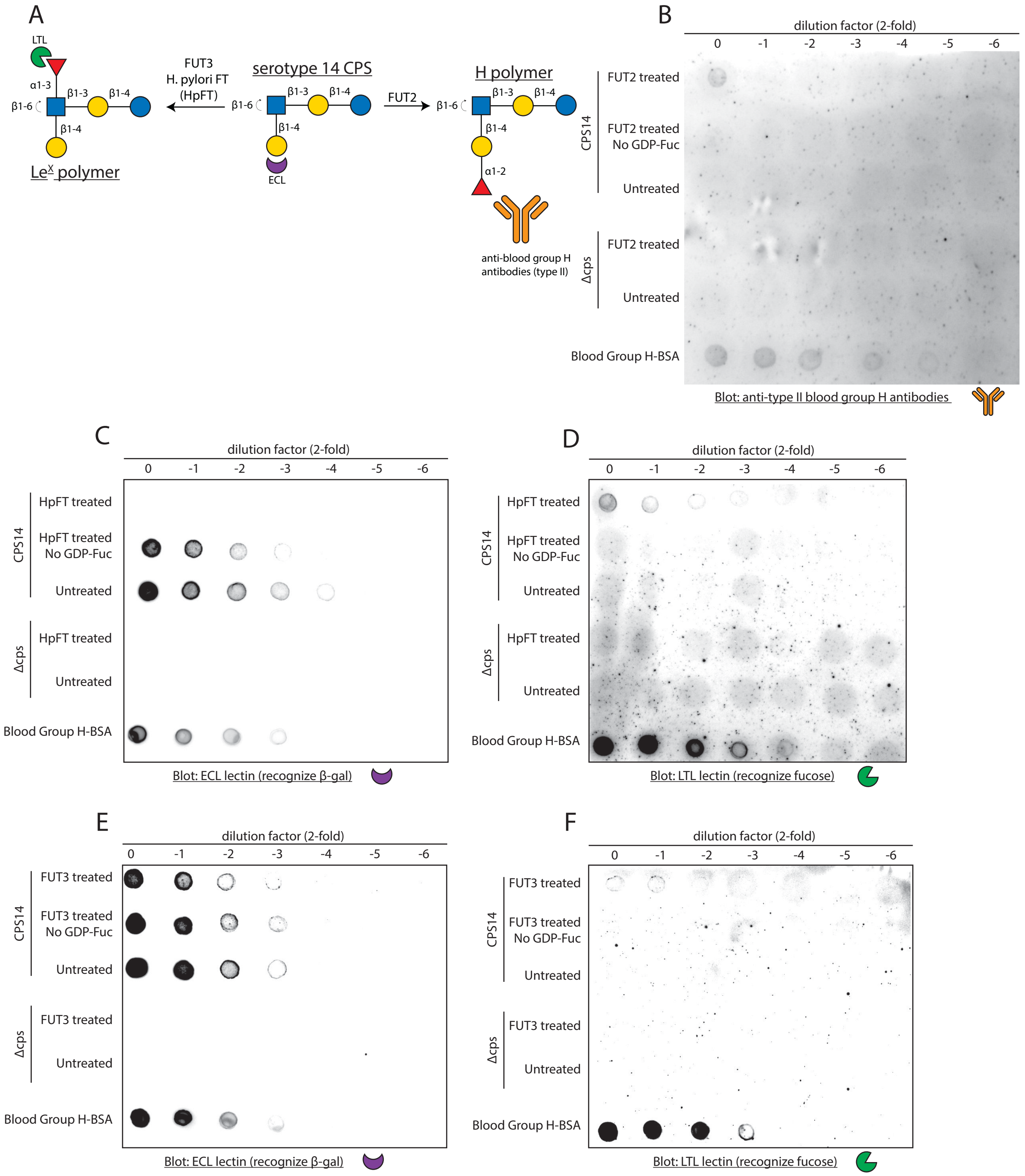

Fig. S20. Synthesis of polymers containing the Lewis X and the blood group H antigens. (**A**) The serotype 14 CPS structurally resembles paragloboside (nLC4), when modified by human α1-3 fucosyltransferase (FUT3) or *Helicobacter pylori* fucosyltransferase (HpFT), produces the Lewis X antigen that interacts with the LTL lectin. Alternatively, the CPS can be modified by human α1-2 fucosyltransferase (FUT2) to make the blood group H antigen. (**B**) Detecting the blood group H antigen by dot blotting. Cells of strain NUS0403 [isogenic serotype 14 capsule-switch mutant;

CPS14] and NUS0483 [Δ*cps*] were heat-inactivated and treated with FUT2 with or without GDP-fucose (GDP-Fuc). Samples were serially diluted and spotted onto a nitrocellulose membrane. The adduct was detected using antibodies specific to the type II blood group H antigen. The blood group H antigen conjugated to bovine serum albumin (blood group H-BSA) was included as the positive control. Shown is the representative blot from three biological replicates. Synthesis of the Lewis X antigen using HpFT (**C** and **D**) and FUT3 (**E** and **F**). Heat-inactivated cells from NUS0403 and NUS0483 were reacted with the indicated fucosyltransferase and spotted on a nitrocellulose membrane. Fucosylation was detected by the increase in LTL lectin binding (**D** and **F**) with a concomitant reduction in ECL binding (**C** and **E**). Untreated cells, Δ*cps* cells, and the no GDP-Fuc added controls were included for comparison. Shown are the representative blots from three biological replicates.

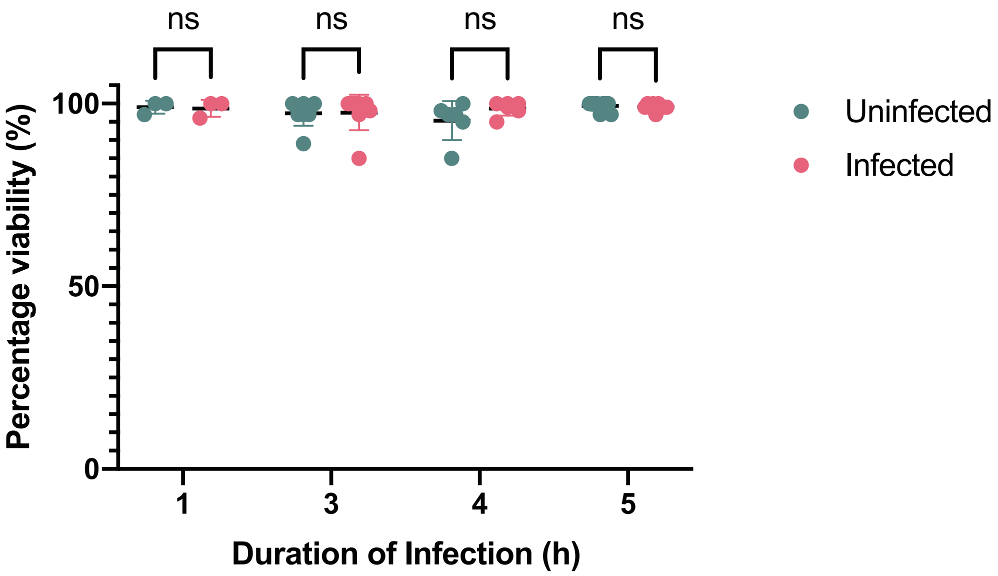

Fig. S21. **Viability of A549 cells when infected with *S. pneumoniae*.** Strain NUS0253 [the serotype 39 capsule-switch mutant] was grown in BHI broth to the mid-log phase. Bacterial cells were collected by centrifugation, resuspended in DMEM, and introduced to A549 cells. The attached A549 cells were released by trypsinization at the indicated time points for measuring cell viability using the trypan blue exclusion assay. P-values were calculated with Mann-Whitney U-tests. n.s.; not significant. Shown are the percentages of viable cells from three biological replicates.

Table S1. Nomenclature of the glycosyltransferases in this study.

Serotype	Gene name (*38*)	Corresponding to
2	*wchH*^{2} *(cps2G)*	4GT^{2}
	wchI^{2} *(cps2I)*	5GT^{2}
5	*whaD*^{5}	4GT^{5}
7A	*wcwH*^{7A}	5GT^{7A}
7F	*wcwH*^{7F}	5GT^{7F}
	wcwD^{7F}	6GT^{7F}
10F	*wcrF*^{10F}	3GT^{10F}
	wcrH^{10F}	5GT^{10F}
10A	*wcrB*^{10A}	2GT^{10A}
	wcrC^{10A}	3GT^{10A}
10B	*wcrF*^{10B}	3GT^{10B}
10C	*wcrB*^{10C}	2GT^{10C}
	wcrC^{10C}	3GT^{10C}
	wcrH^{10C}	5GT^{10C}
15A	*wchX*^{15A}	5GT^{15A}
15B	*wchN*^{15B}	4GT^{15B}
	wchX^{15B}	5GT^{15B}
15C	*wchN*^{15C}	4GT^{15C}
	wchX^{15C}	5GT^{15C}
16F	*wciU*^{16F}	2GT^{16F}
	wcxP^{16F}	4GT^{16F}
	wcxQ^{16F}	5GT^{16F}
18A	*wciU*^{18A}	2GT^{18A}
18B	*wciW*^{18B}	4GT^{18B}
	wciY^{18B}	5GT^{18B}
19B	*wchR*^{19B}	5GT^{19B}
19C	*wchR*^{19C}	5GT^{19C}
	wchU^{19C}	6GT^{19C}
23A	*wchW*^{23A}	3GT^{23A}
	wchX^{23A}	4GT^{23A}
23B	*wchX*^{23B}	3GT^{23B}
23F	*wchW*^{23F}	3GT^{23F}
	wchX^{23F}	4GT^{23F}
24B	*rbsF*^{24B}	4GT^{24B}
	wcxG^{24B}	5GT^{24B}
24F	*rbsF*^{24F}	4GT^{24F}
	wcxG^{24F}	5GT^{24F}
27	*whaL*^{27}	4GT^{27}
	wcrN^{27}	5GT^{27}
28A	*wcxP*^{28A}	4GT^{28A}

	wcxQ^{28A}	5GT^{28A}
28F	*wcxP*^{28F}	4GT^{28F}
	wcxQ^{28F}	5GT^{28F}
32A	*wchQ*^{32A}	3GT^{32A}
	wcrN^{32A}	4GT^{32A}
32F	*wchQ*^{32F}	3GT^{32F}
	wcrN^{32F}	4GT^{32F}
33A	*wciF*^{33A}	5GT^{33A}
33B	*wciO*^{33B}	2GT^{33B}
	wcrC^{33B}	3GT^{33B}
33C	*wcrC*^{33C}	3GT^{33C}
33D	*wciO*^{33D}	2GT^{33D}
	wcrC^{33D}	3GT^{33D}
	wciF^{33D}	6GT^{33D}
33F	*wciF*^{33F}	5GT^{33F}
34	*wcrO*^{34}	2GT^{34}
	wcrC^{34}	3GT^{34}
35C	*wcrK*^{35C}	5GT^{35C}
35F	*wcrC*^{35F}	3GT^{35F}
39	*wcjH*^{39}	PGT^{39}
	whaI^{39}	2GT^{39}
	wcrC^{39}	3GT^{39}
41A	*wcrW*^{41A}	5GT^{41A}
	wcrQ^{41A}	6GT^{41A}
47F	*whaI*^{47F}	2GT^{47F}
	wcrC^{47F}	3GT^{47F}
47A	*whaI*^{47A}	2GT^{47A}
	wcrC^{47A}	3GT^{47A}

Table S2. 3GT variants could complement $3GT^{39}$ as the sole copy of 3GT.

Genotype of the recipient[a]	Transformants obtained (colony forming units; CFU)
	Δ*bgaA*::P-*erm*
Δ*bgaA*::P_{Zn}-$3GT^{39}$	>300
$\Delta 3GT^{39}$ // Δ*bgaA*::P_{Zn}-$3GT^{39}$	2[b]
$\Delta 3GT^{39}<>3GT^{10F}$ // Δ*bgaA*::P_{Zn}-$3GT^{39}$	>300
$\Delta 3GT^{39}<>3GT^{10B}$ // Δ*bgaA*::P_{Zn}-$3GT^{39}$	>300
$\Delta 3GT^{39}<>3GT^{33C}$ // Δ*bgaA*::P_{Zn}-$3GT^{39}$	>300
$\Delta 3GT^{39}<>3GT^{34}$ // Δ*bgaA*::P_{Zn}-$3GT^{39}$	>300
$\Delta 3GT^{39}<>3GT^{35F}$ // Δ*bgaA*::P_{Zn}-$3GT^{39}$	>300
$\Delta 3GT^{39}<>3GT^{47F}$ // Δ*bgaA*::P_{Zn}-$3GT^{39}$	>300
$\Delta 3GT^{39}<>3GT^{47A}$ // Δ*bgaA*::P_{Zn}-$3GT^{39}$	>300

[a] Strains are derivatives of NUS0214 [isogenic capsule-switch mutant of serotype 39; *rpsL1* CPS39].
[b] These transformants are likely suppressor mutants because they are unencapsulated.

Table S3. NMR analyses of the 3GT-switch mutants.

Linkage	Residue		Position[a]					
			1	2	3	4	5	6
α(1-1)	Galactose (WT; Strain NUS0214)	H	5.015	3.963	4.029	4.308	4.029	3.809, 3.768
		C	99.26	67.54	79.66	69.3	70.78	61.1
	Galactose (Serotype 39; Literature value (*61*))	H	4.961	3.902	3.987	4.253	3.986	3.74, 3.73
		C	99.2	67.5	79.7	69.2	70.7	61.2
	Ribitol (WT; Strain NUS0214)	H	4.017, 3.668	3.985	3.809	3.975	3.969, 4.113	/
		C	68.89	71.1	71.61	68.72	66.66	/
	Ribitol (Serotype 39; Literature value (*61*))	H	3.958, 3.606	4.051	3.808	3.924	3.984, 4.077	/
		C	68.8	71	71.6	68.7	66.6	/
α(1-2)	Galactose (3GT^{34}, Strain NUS1429)	H	5.244	3.972	4.071	4.315	4.022	3.809
		C	99.39	67.72	79.48	69.19	70.71	61.1
	Galactose (Serotype 34; Literature value (*62*))	H	5.238	3.957	4.008	4.143	4.091	3.747
		C	100.31	68.52	77.92	70.28	72.0	61.8
	Ribitol (3GT^{34}; Strain NUS1429)	H	**3.888, 3.935**	**4.079**	3.809	3.975	3.969, 4.113	/
		C	**61.09**	**79.8**	71.6	68.72	66.66	/
	Ribitol (Serotype 34; Literature value (*62*))	H	3.847, 3.922	4.047	3.869	4.044	4.008, 4.096	/
		C	60.77	80.8	71.24	72.35	67.79	/
α(1-3)	Galactose (3GT^{33C}; Strain NUS1409)	H	5.244	3.972	4.072	4.315	4.022	3.794, 3.813
		C	99.39	67.72	79.6	69.19	70.71	60.98
	Galactose (Serotype 33C; Literature value (*63*))	H	5.19	4.01	4.45	4.25	4.24	3.74, 3.76
		C	97.1	68.5	75.9	69.7	71.9	62.2
	Ribitol (3GT^{33C}; Strain NUS1409)	H	**3.962, 3.935**	4.054	**4.071**	3.975	4.034, 4.113	/
		C	**61.09**	71.83	**79.48**	68.72	66.69	/
	Ribitol (Serotype 33C; Literature value (*63*))	H	3.80, 3.83	4.09	3.91	4.19	4.06, 4.14	/
		C	63.7	72.2	79.6	71.3	68.2	/
α(1-4)	Galactose (3GT^{10F}; Strain NUS1415)	H	5.014	3.97	4.034	4.359	4.022	3.768
		C	98.22	67.33	80.04	68.95	70.71	61.15
	Galactose (Serotype 10F; Literature value (*64*))	H	5.08	3.926	3.97	4.235	4.246	3.74
		C	99.18	68.31	80.84	70.07	71.58	62.21
	Ribitol (3GT^{10F}, Strain NUS1415)	H	**3.719, 3.876**	3.861	3.809	**4.139**	4.042, 4.113	/
		C	**63.04**	71.41	71.61	**78.12**	66.66	/
	Ribitol (Serotype 10F; Literature value (*64*))	H	3.677, 3.826	3.814	3.902	4.086	4.12, 4.203	/
		C	63.95	72.09	71.56	79.14	65.79	/

[a] Highlighted in red are the significant changes in the ribitol residue between the wild type and the indicated mutants.

Table S4. NMR analyses of the double GT-switch mutant.

Linkage	Residue		Positions[a]					
			1	2	3	4	5	6
5-P-6	Galactofuranose (WT; Strain NUS0214)	H	5.272	4.261	4.11	4.095	4.03	3.997, 4.034
		C	109.3	81.52	76.52	83.45	69.62	66.52
	Galactofuranose (Serotype 39; Literature value (*62*))	H	5.22	4.208	4.094	4.092	3.977	3.981, 3.936
		C	109.4	81.5	76.7	82.7	69.4	66.5
5-P-5	Galactofuranose (2GT^{10A} switched; Strain NUS1776)	H	5.261	4.261	4.113	4.108	**4.226**	**3.90, 3.996**
		C	109.1	81.52	76.46	83.27	**76.03**	**61.47**
	Galactofuranose (Serotype 10A; Literature value (*64*))	H	5.225	4.215	4.189	4.21	4.378	3.784, 3.862
		C	110.17	82.73	77.3	82.67	75.77	62.82
α(1-1)	Ribitol (WT; Strain NUS0214)	H	4.017, 3.668	3.985	3.809	3.975	3.969, 4.113	
		C	68.89	71.1	71.61	68.72	66.66	
	Ribitol (Serotype 39; Literature value (*62*))	H	3.958, 3.606	4.051	3.808	3.924	3.984, 4.077	
		C	68.8	71	71.6	68.7	66.6	
α(1-2)	Ribitol (3GT^{10A} switched; Strain NUS1776)	H	**3.884, 3.961**	**4.43**	3.808	3.989	3.976, 4.127	
		C	**61.34**	**79.75**	71.62	73.64	68.59	
	Ribitol (Serotype 10A; Literature value (*64*))	H	3.856, 3.931	4.017	3.879	3.998	4.022, 4.145	
		C	61.36	80.81	71.91	73.18	68.33	

[a] Highlighted in red are the significant changes in the ribitol residue between the wild type and the indicated mutants.

Table S5. *cps23BJ* bypassed the essentiality of the last GTs**

Serotype	Gene deleted	Recipient strain	
		WT	Cps23BJ**
Dispensable			
7F	$6GT^{7F}$	>300	>300
19C	$6GT^{19C}$	>300	>300
23A	$3GT^{23A}$	>300	>300
24B	$4GT^{24B}$	>300	>300
	$5GT^{24B}$	>300	>300
24F	$4GT^{24F}$	>300	>300
	$5GT^{24F}$	>300	>300
Essential in the presence of *cps23BJ***			
5	$4GT^{5}$	0	0
7A	$5GT^{7A}$	0	1
15A	$5GT^{15A}$	0	0
18B	$5GT^{18B}$	0	0
19C	$5GT^{19C}$	3[a]	25[a]
23A	$4GT^{23A}$	0	34[a]
23F	$4GT^{23F}$	0	0
27	$4GT^{27}$	0	0
	$5GT^{27}$	0	0
28F	$5GT^{28F}$	13[a]	21[a]
32F	$3GT^{32F}$	3[a]	4[a]
	$4GT^{32F}$	1[a]	0
33A	$5GT^{33A}$	0	0
33D	$6GT^{33D}$	0	0
33F	$5GT^{33F}$	0	0
35C	$5GT^{35C}$	0	0
41A	$5GT^{41A}$	0	0
	$6GT^{41A}$	0	2
Essentiality likely suppressed by *cps23BJ***			
2	$5GT^{2}$	5[a]	>300
10C	$5GT^{10C}$	53[a]	>300
10F	$5GT^{10F}$	3[a]	207
15B	$4GT^{15B}$	1	84
	$5GT^{15B}$	1	95
15C	$4GT^{15C}$	2	288
	$5GT^{15C}$	4	98
16F	$4GT^{16F}$	0	>300
18B	$4GT^{18B}$	6[a]	>300
23F	$3GT^{23F}$	3	>300
28A	$4GT^{28A}$	0	>300
	$5GT^{28A}$	16[a]	>300
28F	$4GT^{28F}$	4[a]	>300

[a] These mutants are likely suppressor mutants because they are unencapsulated. Alternatively, PCR diagnosis of these mutants showed that the corresponding GTs were not deleted.
[b] These strains are likely unstable as they could not grow upon re-streaking or in the BHI medium.

Table S6. *wzk* and *wzxC* variants could not complement *ytgP* in *S. pneumoniae*.

Genotype of the recipient[a]	Transformants obtained when the indicated amplicon was introduced (CFU)		
	None	Δ*pgdA*::P-*erm*[b]	Δ*ytgP*::P-*erm*
Δ*cps2J*<>*ytgP* // Δ*bgaA*::P_{Zn}-*cps2J*	not tested	not tested	>300
Δ*cps2J*<>*wzxC*-FLAG^{A33V} // Δ*bgaA*::P_{Zn}-*cps2J*	0	>300	6[c]
Δ*cps2J*<>*wzk* // Δ*bgaA*::P_{Zn}-*cps2J*	0	>300	5[c]

[a] Strains are derivatives of IU1781 [D39 *rpsL1*].
[b] The Δ*pgdA*::P-*erm* cassette was used as the transformation efficiency control.
[c] PCR diagnosis showed that the corresponding genes were not deleted in these isolates.

Table S7. *cps23BJ** variants could not transport the lipid-linked precursor of serotype 14 CPS.

**Genotype of the recipient**[a]	**Transformants obtained when the indicated amplicon was introduced (CFU)**		
	None	Δ*pgdA*::P-*erm*[b]	Δ*cps14J*::P-*erm*
ΔCEP::P-*kan-cps14J*	0	>300	>300
Δ*bgaA*::P_{spxB}-*cps23BJ*^{P254S}	0	>300	9[c]
Δ*bgaA*::P_{spxB}-*cps23BJ*^{D231G}	0	>300	3[c]

[a] Strains are derivatives of NUS0403 [isogenic capsule-switch mutant of serotype 14; *rpsL1* CPS14].
[b] The Δ*pgdA*::P-*erm* cassette was used as a transformation efficiency control.
[c] PCR diagnosis showed that the corresponding genes were not deleted in these isolates.

Table S8. *cps23J*** (P-*cps23J*$^{P254S\ T38M}$) bypasses the essentiality of *cps2I* (5GT^2) and *cps2K*, but not *cps2G* (4GT^2).

**Genotype of the recipient**[a]	**Transformants obtained when the indicated amplicon was introduced (colony forming units; CFU)**			
	Δ*cps2I*::P-*erm*	Δ*cps2K*::P-*erm*	Δ*cps2G*::P-*erm*	Δ*pgdA*::P-*erm*[c]
Wild type	5[b]	33[b]	2[b]	>300
Δ*cps2E*	>300	>300	>300	>300
P-*cps23BJ***	>300	>300	12[b]	>300

[a] Strains are derivatives of IU1781 [D39 *rpsL1*].
[b] The survivors on these plates (<50 CFU) are likely suppressors because they do not produce CPS.
[c] The Δ*pgdA*::P-*erm* cassette was used as a transformation efficiency control.

Table S9. *cps23J*** allows simultaneous deletions of *cps2I* (5GT2), *cps2J* (flippase), and *cps2K* (UDP-GlcUA synthase).

**Genotype of the recipient**[a]	**Transformants obtained (CFU)**
	Δ*cps2IJK*::P-*erm*
Wild type	0
Δ*cps2E*	>300
P-*cps23J*$^{P254S\ T38M}$	>300
P-*cps23J*$^{P254S\ I246T}$	>300
P-*cps23J*$^{P254S\ Q276L}$	>300
P-*cps23J*$^{P254S\ L414P}$	>300

[a] Strains are derivatives of IU1781 [D39 *rpsL1*].

Table S10. *cps2H* (polymerase) remained essential in the *cps23J*** background.

Genotype of the recipient[a]	Amplicon transformed	Transformants obtained (CFU)
Δ*cps2I-K*::P-*erm* // P-*cps23J*$^{P254S\ T38M}$	Δ*cps2H*::P-*spec-rpsL*$^{+}$	36[b]
Δ*cps2I-K*::P-*erm* // P-*cps23J*$^{P254S\ I246T}$	Δ*cps2H*::P-*spec-rpsL*$^{+}$	11[b]
Δ*cps2I-K*::P-*erm* // P-*cps23J*$^{P254S\ Q276L}$	Δ*cps2H*::P-*spec-rpsL*$^{+}$	9[b]
Δ*cps2I-K*::P-*erm* // P-*cps23J*$^{P254S\ L414P}$	Δ*cps2H*::P-*spec-rpsL*$^{+}$	7[b]
Δ*cps2E* Δ*cps2I-K*::P-*erm* // P-*cps23J*$^{P254S\ T38M}$	Δ*cps2H*::P-*spec-rpsL*$^{+}$	>300
Δ*cps2E* Δ*cps2I-K*::P-*erm* // P-*cps23J*$^{P254S\ I246T}$	Δ*cps2H*::P-*spec-rpsL*$^{+}$	>300
Δ*cps2E* Δ*cps2I-K*::P-*erm* // P-*cps23J*$^{P254S\ Q276L}$	Δ*cps2H*::P-*spec-rpsL*$^{+}$	>300
Δ*cps2E* Δ*cps2I-K*::P-*erm* // P-*cps23J*$^{P254S\ L414P}$	Δ*cps2H*::P-*spec-rpsL*$^{+}$	>300

[a] Strains are derivatives of IU1781 [D39 *rpsL1*].
[b] PCR diagnosis showed that the corresponding genes were not deleted in these isolates.

Table S11. Strains used in this study

Strain	Relevant genotype[a,b]	Derivation	Selectable marker[c]	Source
IU1690	Serotype 2 strain D39W	-	None	(*65*)
IU1781	*rpsL1*	-	Str^R	(*66*)
NUH0007	Serotype 14 clinical isolate	-	None	(*5*)
PATH344	Serotype 33C clinical isolate	-	None	(*5*)
PATH691	Serotype 10A clinical isolate	-	None	(*5*)
PATH1539	Serotype 10F clinical isolate	-	None	(*5*)
PATH1702	Serotype 16F clinical isolate	-	None	(*5*)
PATH1748	Serotype 34 clinical isolate	-	None	CDC, USA
PATH1945	Serotype 33B clinical isolate	-	None	(*5*)
PATH2009	Serotype 39 clinical isolate	-	None	
PATH2459	Serotype 10B clinical isolate	-	None	(*5*)
PATH2460	Serotype 10C clinical isolate	-	None	(*5*)
PATH2463	Serotype 19C clinical isolate	-	None	(*5*)
PATH2475	Serotype 47F clinical isolate	-	None	(*5*)
PATH2476	Serotype 47A clinical isolate	-	None	(*5*)
PATH2481	Serotype 33D clinical isolate	-	None	(*5*)
PATH2606	Serotype 19B clinical isolate	-	None	(*5*)
PATH4560	Serotype 18A clinical isolate	-	None	(*5*)
PATH46	Serotype 5 clinical isolate	-	None	(*5*)
PATH2477	Serotype 7A clinical isolate	-	None	(*5*)
NUH0005	Serotype 7F clinical isolate	-	None	(*5*)
NUH0008	Serotype 15A clinical isolate	-	None	(*5*)

NUH0009	Serotype 15B clinical isolate	-	None	(*5*)
NUH0010	Serotype 15C clinical isolate	-	None	(*5*)
PATH269	Serotype 18B clinical isolate	-	None	(*5*)
NUH0016	Serotype 23A clinical isolate	-	None	(*5*)
NUH0017	Serotype 23F clinical isolate	-	None	(*5*)
PATH2465	Serotype 24B clinical isolate	-	None	(*5*)
PATH20	Serotype 24F clinical isolate	-	None	(*5*)
PATH2467	Serotype 27 clinical isolate	-	None	(*5*)
PATH9002	Serotype 28A clinical isolate	-	None	(*5*)
PATH382	Serotype 28F clinical isolate	-	None	(*5*)
PATH2468	Serotype 32F clinical isolate	-	None	(*5*)
PATH1754	Serotype 33A clinical isolate	-	None	(*5*)
PATH101	Serotype 33F clinical isolate	-	None	(*5*)
PATH1895	Serotype 35C clinical isolate	-	None	(*5*)
PATH2471	Serotype 41A clinical isolate	-	None	(*5*)
NUS0016	*rpsL1 ΔbgaA*::P-*kan-rpsL*$^{+}$	Δ*bgaA*::P-*kan-rpsL*$^{+}$ x IU1781	Kan^{R}	(*5*)
NUS0029	*rpsL1 ΔcpsE*::P-*spec-rpsL*$^{+}$	Δ*cpsE*::P-*spec-rpsL*$^{+}$ x IU1781	Spec^{R}	This study
NUS0064	*rpsL1 Δcps2J*::P-*kan-rpsL ΔbgaA*::P$_{Zn}$-*cps2J*	-	Kan^{R}	(*5*)
NUS0759	*rpsL1 Δcps2J<>ytgP ΔbgaA*::P$_{Zn}$-*cps2J*	Δ*cps2J<>ytgP* x NUS0064	Str^{R}	(*5*)
NUS1963	*rpsL1* CPS23B Δ*bgaA*::P-*kan-cps23BJ*^{P254S}	-	Kan^{R}, Str^{R}	(*5*)
HMS0002	*rpsL1 ΔcpsE*	-	Str^{R}	(*5*)
HMS0019	*rpsL1* CEP::P-*kan-rpsL*$^{+}$	CEP::P-*kan-rpsL*$^{+}$ x IU1781	Kan^{R}, Str^{S}	(*5*)
NUS0114	*rpsL1 Δcps*::P-*sacB-kan-rpsL*$^{+}$	Δ*cps*::P-*sacB-kan-rpsL*$^{+}$ x IU1781	Kan^{R}, Str^{S}, Suc^{S}	(*5*)
NUS0403	*rpsL1* CPS14	DNA(NUH0007) x NUS0114	Str^{R}	(*43*)

NUS0214	*rpsL1* CPS39	DNA(PATH2009) x NUS0114	Str^R	(*43*)
NUS0215	*rpsL1* CPS10C	DNA(PATH2460) x NUS0114	Str^R	(*43*)
NUS0253	*rpsL1* CPS39	DNA(PATH2009) x NUS0114	Str^R	(*43*)
NUS0312	*rpsL1* CPS10A	DNA(PATH691) x NUS0114	Str^R	(*43*)
NUS0672	*rpsL1* CPS33D	DNA(PATH2481) x NUS0114	Str^R	(*43*)
NUS0391	*rpsL1* CPS18A	DNA(PATH4560) x NUS0114	Str^R	(*43*)
NUS0327	*rpsL1* CPS5	DNA(PATH46) x NUS0114	Str^R	(*43*)
NUS0667	*rpsL1* CPS7A	DNA(PATH2477) x NUS0114	Str^R	(*43*)
NUS0310	*rpsL1* CPS7F	DNA(NUH0005) x NUS0114	Str^R	(*43*)
NUS0280	*rpsL1* CPS10F	DNA(PATH1539) x NUS0114	Str^R	(*43*)
NUS0315	*rpsL1* CPS10C	DNA(PATH2460) x NUS0114	Str^R	(*43*)
NUS0999	*rpsL1* CPS15A	DNA(NUH0008) x NUS0114	Str^R	(*43*)
NUS0340	*rpsL1* CPS15B	DNA(NUH0009) x NUS0114	Str^R	(*43*)
NUS0289	*rpsL1* CPS15C	DNA(NUH0010) x NUS0114	Str^R	(*43*)
NUS0292	*rpsL1* CPS16F	DNA(PATH1702) x NUS0114	Str^R	(*43*)
NUS0330	*rpsL1* CPS18B	DNA(PATH269) x NUS0114	Str^R	(*43*)
NUS0317	*rpsL1* CPS19B	DNA(PATH2606) x NUS0114	Str^R	(*43*)
NUS0316	*rpsL1* CPS19C	DNA(PATH2463) x NUS0114	Str^R	(*43*)
NUS0399	*rpsL1* CPS23A	DNA(NUH0016) x NUS0114	Str^R	(*43*)
NUS0389	*rpsL1* CPS23F	DNA(NUH0017) x NUS0114	Str^R	(*43*)
NUS0247	*rpsL1* CPS24B	DNA(PATH2465) x NUS0114	Str^R	(*43*)
NUS0311	*rpsL1* CPS24F	DNA(PATH20) x NUS0114	Str^R	(*43*)
NUS0463	*rpsL1* CPS27	DNA(PATH2467) x NUS0114	Str^R	(*43*)
NUS0671	*rpsL1* CPS28A	DNA(PATH9002) x NUS0114	Str^R	(*43*)
NUS0390	*rpsL1* CPS28F	DNA(PATH382) x NUS0114	Str^R	(*43*)

NUS0342	*rpsL1* CPS32F	DNA(PATH2468) x NUS0114	Str^{R}	(*43*)
NUS0332	*rpsL1* CPS33A	DNA(PATH1754) x NUS0114	Str^{R}	(*43*)
NUS0326	*rpsL1* CPS33F	DNA(PATH101) x NUS0114	Str^{R}	(*43*)
NUS0308	*rpsL1* CPS33B	DNA(PATH1945) x NUS0114	Str^{R}	(*43*)
NUS0290	*rpsL1* CPS33C	DNA(PATH344) x NUS0114	Str^{R}	(*43*)
NUS0672	*rpsL1* CPS33D	DNA(PATH2481) x NUS0114	Str^{R}	(*43*)
NUS0369	*rpsL1* CPS35C	DNA(PATH1895) x NUS0114	Str^{R}	(*43*)
NUS0374	*rpsL1* CPS41A	DNA(PATH2471) x NUS0114	Str^{R}	(*43*)
NUS0747	*rpsL1* CPS39 Δ*wcrC*<>*wcrC*$^{10A(F95S)}$ -FLAG // Δ*bgaA*::P$_{Zn}$-*wcrC*^{39}	Δ*wcrC*<>*wcrC*$^{10A(F95S)}$ -FLAG x NUS1303	Str^{R}	This study
NUS0748	*rpsL1* CPS39 Δ*wcrC*<>*wcrC*$^{10C(F95S)}$ -FLAG // Δ*bgaA*::P$_{Zn}$-*wcrC*^{39}	Δ*wcrC*<>*wcrC*$^{10C(F95S)}$ -FLAG x NUS1303	Str^{R}	This study
NUS1262	*rpsL1* CPS18A // Δ*bgaA*::P-*sacB-kan-rpsL*$^{+}$	Δ*bgaA*::P-*sacB-kan-rpsL*$^{+}$ x NUS0391	Kan^{R}	This study
NUS1289	*rpsL1* CPS18A // Δ*bgaA*::P$_{Zn}$-*wciU*^{18A}	Δ*bgaA*::P$_{Zn}$-*wciU*^{18A} x NUS1262	Str^{R}	This study
NUS1211	*rpsL1* CPS39 // Δ*bgaA*::P-*sacB-kan-rpsL*$^{+}$	Δ*bgaA*::P-*sacB-kan-rpsL*$^{+}$ x NUS0253	Kan^{R}, Str^{S}, Suc^{S}	This study
NUS1223	*rpsL1* CPS39 // Δ*bgaA*::P$_{Zn}$-*wcrC*^{39}	Δ*bgaA*::P$_{Zn}$-*wcrC*^{39} x NUS1211	Str^{R}	This study
NUS1303	*rpsL1* CPS39 Δ*wcrC*::P-*sacB-kan-rpsL*$^{+}$ // Δ*bgaA*::P$_{Zn}$-*wcrC*^{39}	Δ*wcrC*::P-*sacB-kan-rpsL*$^{+}$ x NUS1223	Kan^{R}, Str^{S}, Suc^{S}	This study
NUS1355	*rpsL1* CPS39 Δ*wcrC*<>*wcrC*^{33B} // Δ*bgaA*::P$_{Zn}$-*wcrC*^{39}	Δ*wcrC*<>*wcrC*^{33B} x NUS1303	Str^{R}	This study
NUS1356	*rpsL1* CPS39 Δ*wcrC*<>*wcrC*^{33C} // Δ*bgaA*::P$_{Zn}$-*wcrC*^{39}	Δ*wcrC*<>*wcrC*^{33C} x NUS1303	Str^{R}	This study
NUS1358	*rpsL1* CPS39 Δ*wcrC*<>*wcrC*^{47A} // Δ*bgaA*::P$_{Zn}$-*wcrC*^{39}	Δ*wcrC*<>*wcrC*^{47A} x NUS1303	Str^{R}	This study
NUS1365	*rpsL1* CPS39 Δ*wcjH*::P-*erm*	Δ*wcjH*::P-*erm* x NUS0253	Erm^{R}	This study

NUS1357	*rpsL1* CPS39 Δ*wcrC*<>*wcrC*^{33D} // Δ*bgaA*::P$_{Zn}$-*wcrC*^{39}	Δ*wcrC*<>*wcrC*^{33D} x NUS1303	Str^{R}	This study
NUS1407	*rpsL1* CPS39 Δ*wcrC*<>*wcrF*^{10F} // Δ*bgaA*::P$_{Zn}$-*wcrC*^{39}	Δ*wcrC*<>*wcrF*^{10F} x NUS1303	Str^{R}	This study
NUS1408	*rpsL1* CPS39 Δ*wcrC*<>*wcrF*^{10B} // Δ*bgaA*::P$_{Zn}$-*wcrC*^{39}	Δ*wcrC*<>*wcrF*^{10B} x NUS1303	Str^{R}	This study
NUS1409	*rpsL1* CPS39 Δ*wcrC*<>*wcrC*^{33C} // Δ*bgaA*::P-*erm*	Δ*bgaA*::P-*erm* x NUS1356	Str^{R}, Erm^{R}	This study
NUS1410	*rpsL1* CPS39 Δ*wcrC*<>*wcrC*^{47A} // Δ*bgaA*::P-*erm*	Δ*bgaA*::P-*erm* x NUS1358	Str^{R}, Erm^{R}	This study
NUS1415	*rpsL1* CPS39 Δ*wcrC*<>*wcrF*^{10F} // Δ*bgaA*::P-*erm*	Δ*bgaA*::P-*erm* x NUS1407	Str^{R}, Erm^{R}	This study
NUS1416	*rpsL1* CPS39 Δ*wcrC*<>*wcrF*^{10B} // Δ*bgaA*::P-*erm*	Δ*bgaA*::P-*erm* x NUS1408	Str^{R}, Erm^{R}	This study
NUS1426	*rpsL1* CPS39 Δ*wcrC*<>*wcrC*^{34} // Δ*bgaA*::P$_{Zn}$-*wcrC*^{39}	Δ*wcrC*<>*wcrC*^{34} x NUS1303	Str^{R}	This study
NUS1427	*rpsL1* CPS39 Δ*wcrC*<>*wcrC*^{35F} // Δ*bgaA*::P$_{Zn}$-*wcrC*^{39}	Δ*wcrC*<>*wcrC*^{35F} x NUS1303	Str^{R}	This study
NUS1428	*rpsL1* CPS39 Δ*wcrC*<>*wcrC*^{47F} // Δ*bgaA*::P$_{Zn}$-*wcrC*^{39}	Δ*wcrC*<>*wcrC*^{47F} x NUS1303	Str^{R}	This study
NUS1429	*rpsL1* CPS39 Δ*wcrC*<>*wcrC*^{34} // Δ*bgaA*::P-*erm*	Δ*bgaA*::P-*erm* x NUS1426	Str^{R}, Erm^{R}	This study
NUS1430	*rpsL1* CPS39 // Δ*bgaA*::P$_{Zn}$-*whaI*^{39}	Δ*bgaA*::P$_{Zn}$-*whaI*^{39} x NUS1211	Str^{R}	This study
NUS1463	*rpsL1* CPS39 Δ*whaI*::P-*sacB-kan-rpsL*$^{+}$ // Δ*bgaA*::P$_{Zn}$-*whaI*^{39}	Δ*whaI*::P-*sacB-kan-rpsL*$^{+}$ x NUS1430	Kan^{R}, Str^{S}, Suc^{S}	This study
NUS1465	*rpsL1* CPS39 Δ*wcrC*<>*wcrC*^{35F} // Δ*bgaA*::P-*erm*	Δ*bgaA*::P-*erm* x NUS1427	Str^{R}, Erm^{R}	This study
NUS1466	*rpsL1* CPS39 Δ*wcrC*<>*wcrC*^{47F} // Δ*bgaA*::P-*erm*	Δ*bgaA*::P-*erm* x NUS1428	Str^{R}, Erm^{R}	This study
NUS1479	*rpsL1* CPS39 Δ*wcrC*<>*wcrC*^{10A} // Δ*bgaA*::P$_{Zn}$-*wcrC*^{39}	Δ*wcrC*<>*wcrC*^{10A} x NUS1303	Str^{R}	This study

NUS1480	*rpsL1* CPS39 Δ*wcrC*<>*wcrC*^{10C} // Δ*bgaA*::P$_{Zn}$-*wcrC*^{39}	Δ*wcrC*<>*wcrC*^{10C} x NUS1303	Str^{R}	This study
NUS1485	*rpsL1* CPS39 Δ*wcrC*<>*wcrC*^{33D} // Δ*bgaA*::P$_{Zn}$-*wcrC*^{39} // CEP::P-*kan-rpsL*$^{+}$	CEP::P-*kan-rpsL*$^{+}$ x NUS1357	Kan^{R}, Str^{S}	This study
NUS1486	*rpsL1* CPS39 Δ*wcrC*<>*wcrC*^{10A} // Δ*bgaA*::P$_{Zn}$-*wcrC*^{39} // CEP::P-*kan-rpsL*$^{+}$	CEP::P-*kan-rpsL*$^{+}$ x NUS1479	Kan^{R}, Str^{S}	This study
NUS1487	*rpsL1* CPS39 Δ*wcrC* // Δ*bgaA*::P$_{Zn}$-*wcrC*^{39}	Δ*wcrC* x NUS1303	Str^{R}, Suc^{R}	This study
NUS1552	*rpsL1* CPS10A // CEP::P-*kan-wcrB*^{10A}	CEP::P-*kan-wcrB*^{10A} x NUS0312	Kan^{R}	This study
NUS1553	*rpsL1* CPS33D // CEP::P-*kan-wciO*^{33D}	CEP::P-*kan-wciO*^{33D} x NUS0672	Kan^{R}	This study
NUS1554	*rpsL1* CPS39 Δ*wcrC*<>*wcrC*^{10A} // Δ*bgaA*::P$_{Zn}$-*wcrC*^{39} // CEP::P-*kan-wcrB*^{10A}	CEP::P-*kan-wcrB*^{10A} x NUS1486	Kan^{R}	This study
NUS1555	*rpsL1* CPS39 Δ*wcrC*<>*wcrC*^{33D} // Δ*bgaA*::P$_{Zn}$-*wcrC*^{39} // CEP::P-*kan-wciO*^{33D}	CEP::P-*kan-wciO*^{33D} x NUS1486	Kan^{R}	This study
NUS1586	*rpsL1* CPS10A Δ*wcrB*::P-*erm* // CEP::P-*kan-wcrB*^{10A}	Δ*wcrB*::P-*erm* x NUS1552	Kan^{R}, Erm^{R}	This study
NUS1587	*rpsL1* CPS33D Δ*wciO*::P-*erm* // CEP::P-*kan-wciO*^{33D}	Δ*wciO*::P-*erm* x NUS1553	Kan^{R}, Erm^{R}	This study
NUS1603	*rpsL1* CPS39 Δ*wcrC*<>*wcrC*$^{34\ D129A}$ // Δ*bgaA*::P$_{Zn}$-*wcrC*^{39}	Δ*wcrC*<>*wcrC*$^{34\ D129A}$ x NUS1303	Str^{R}	This study
NUS1604	*rpsL1* CPS39 Δ*wcrC*<>*wcrC*$^{35F\ D123A}$ // Δ*bgaA*::P$_{Zn}$-*wcrC*^{39}	Δ*wcrC*<>*wcrC*$^{35F\ D123A}$ x NUS1303	Str^{R}	This study
NUS1605	*rpsL1* CPS39 Δ*wcrC*<>*wcrC*$^{47F\ D122A}$ // Δ*bgaA*::P$_{Zn}$-*wcrC*^{39}	Δ*wcrC*<>*wcrC*$^{47F\ D122A}$ x NUS1303	Str^{R}	This study
NUS1606	*rpsL1* CPS39 Δ*wcrC*<>*wcrC*$^{47A\ D122A}$ // Δ*bgaA*::P$_{Zn}$-*wcrC*^{39}	Δ*wcrC*<>*wcrC*$^{47A\ D122A}$ x NUS1303	Str^{R}	This study
NUS1607	*rpsL1* CPS39 Δ*wcrC*<>*wcrC*$^{34\ S102F}$ // Δ*bgaA*::P$_{Zn}$-*wcrC*^{39}	Δ*wcrC*<>*wcrC*$^{34\ S102F}$ x NUS1303	Str^{R}	This study

NUS1608	*rpsL1* CPS39 Δ*wcrC*<>*wcrC*$^{35F\ S96F}$ // Δ*bgaA*::P$_{Zn}$-*wcrC*^{39}	Δ*wcrC*<>*wcrC*$^{35F\ S96F}$ x NUS1303	Str^{R}	This study
NUS1609	*rpsL1* CPS39 Δ*wcrC*<>*wcrC*$^{47F\ S95F}$ // Δ*bgaA*::P$_{Zn}$-*wcrC*^{39}	Δ*wcrC*<>*wcrC*$^{47F\ S95F}$ x NUS1303	Str^{R}	This study
NUS1610	*rpsL1* CPS39 Δ*wcrC*<>*wcrC*$^{47A\ S95F}$ // Δ*bgaA*::P$_{Zn}$-*wcrC*^{39}	Δ*wcrC*<>*wcrC*$^{47A\ S95F}$ x NUS1303	Str^{R}	This study
NUS1643	*rpsL1* CPS39 Δ*wcrC*<>*wcrC*$^{10A\ F95S}$ // Δ*bgaA*::P$_{Zn}$-*wcrC*^{39}	Δ*wcrC*<>*wcrC*$^{10A\ F95S}$ x NUS1303	Str^{R}	This study
NUS1644	*rpsL1* CPS39 Δ*whaI*::P-*erm* Δ*wcrC*<>*wcrC*^{10A} // Δ*bgaA*::P$_{Zn}$-*wcrC*^{39} // CEP::P-*kan-wcrB*^{10A}	Δ*whaI*::P-*erm* x NUS1554	Kan^{R}, Erm^{R}	This study
NUS1645	*rpsL1* CPS39 Δ*whaI*::P-*erm* Δ*wcrC*<>*wcrC*^{33D} // Δ*bgaA*::P$_{Zn}$-*wcrC*^{39} // CEP::P-*kan-wciO*^{33D}	Δ*whaI*::P-*erm* x NUS1555	Kan^{R}, Erm^{R}	This study
NUS1662	*rpsL1* CPS39 Δ*wcrC*<>*wcrC*$^{10C\ F95S}$ // Δ*bgaA*::P$_{Zn}$-*wcrC*^{39}	Δ*wcrC*<>*wcrC*$^{10C\ F95S}$ x NUS1303	Str^{R}	This study
NUS1776	*rpsL1* CPS39 Δ*whaI*::P-*erm* Δ*wcrC*<>*wcrC*^{10A} // Δ*bgaA*::P-*spec-rpsL*$^{+}$ // CEP::P-*kan-wcrB*^{10A}	Δ*bgaA*::P-*spec-rpsL*$^{+}$ x NUS1644	Kan^{R}, Spec^{R} Erm^{R}	This study
NUS1777	*rpsL1* CPS39 Δ*whaI*::P-*erm* Δ*wcrC*<>*wcrC*^{33D} // Δ*bgaA*::P-*spec-rpsL*$^{+}$ // CEP::P-*kan-wciO*^{33D}	Δ*bgaA*::P-*spec-rpsL*$^{+}$ x NUS1645	Kan^{R}, Spec^{R} Erm^{R}	This study
NUS1850	*rpsL1* CPS39 Δ*wcrC*^{39}<>*wcrC*$^{10A\ F95S}$ // Δ*bgaA*::P-*erm*	Δ*bgaA*::P-*erm* x NUS1643	Str^{R}, Erm^{R}	This study
NUS1852	*rpsL1* CPS39 Δ*wcrC*^{39}<>*wcrC*$^{10C\ F95S}$ // Δ*bgaA*::P-*erm*	Δ*bgaA*::P-*erm* x NUS1662	Str^{R}, Erm^{R}	This study
NUS1874	*rpsL1* CPS47A // Δ*bgaA*::P-*sacB-kan-rpsL*$^{+}$	Δ*bgaA*::P-*sacB-kan-rpsL*$^{+}$ x NUS1080	Kan^{R}	This study
NUS1895	*rpsL1* CPS39 Δ*wcrC*^{39}<>*wcrC*^{34} // Δ*bgaA*::P-*kan-wcrO*^{34}	Δ*bgaA*::P-*kan-wcrO*^{34} x NUS1429	Kan^{R}	This study

NUS1921	*rpsL1* CPS47A // Δ*bgaA*::P$_{Zn}$-*wcrC*^{47A}	Δ*bgaA*::P$_{Zn}$-*wcrC*^{47A} x NUS1874	Str^{R}	This study
NUS1937	*rpsL1* CPS39 Δ*whaI*^{39}<>*whaI*^{47F} // Δ*bgaA*::P$_{Zn}$-*whaI*^{39}	Δ*whaI*^{39}<>*whaI*^{47F} x NUS1463	Str^{R}	This study
NUS1938	*rpsL1* CPS39 Δ*whaI*^{39}<>*whaI*^{47A} // Δ*bgaA*::P$_{Zn}$-*whaI*^{39}	Δ*whaI*^{39}<>*whaI*^{47A} x NUS1463	Str^{R}	This study
NUS1939	*rpsL1* CPS34 // Δ*bgaA*::P$_{Zn}$-*wcrC*^{34}	Δ*bgaA*::P$_{Zn}$-*wcrC*^{34} x NUS1374	Str^{R}	This study
NUS1945	*rpsL1* CPS39 Δ*whaI*^{39}<>*whaI*^{47F} // Δ*bgaA*::P-*erm*	Δ*bgaA*::P-*erm* x NUS1937	Str^{R}, Erm^{R}	This study
NUS1946	*rpsL1* CPS39 Δ*whaI*^{39}<>*whaI*^{47A} // Δ*bgaA*::P-*erm*	Δ*bgaA*::P-*erm* x NUS1938	Str^{R}, Erm^{R}	This study
NUS1968	*rpsL1* CPS18A Δ*wciU*^{18A}::P-*sacB-kan-rpsL*$^{+}$ // Δ*bgaA*::P$_{Zn}$-*wciU*^{18A}	Δ*wciU*^{18A}::P-*sacB-kan-rpsL*$^{+}$ x NUS1289	Kan^{R}	This study
NUS1969	*rpsL1* CPS34 Δ*wcrC*^{34}::P-*sacB-kan-rpsL*$^{+}$ // Δ*bgaA*::P$_{Zn}$-*wcrC*^{34}	Δ*wcrC*^{34}::P-*sacB-kan-rpsL*$^{+}$ x NUS1939	Kan^{R}	This study
NUS1970	*rpsL1* CPS47A Δ*wcrC*^{47A}::P-*sacB-kan-rpsL*$^{+}$ // Δ*bgaA*::P$_{Zn}$-*wcrC*^{47A}	Δ*wcrC*^{47A}::P-*sacB-kan-rpsL*$^{+}$ x NUS1921	Kan^{R}	This study
NUS2013	*rpsL1* CPS10C // ΔCEP::P-*kan-wcrB*^{10C}	ΔCEP::P-*kan-wcrB*^{10C} x NUS0315	Kan^{R}	This study
NUS2014	*rpsL1* CPS33B // ΔCEP::P-*kan-wciO*^{33B}	ΔCEP::P-*kan-wciO*^{33B} x NUS0308	Kan^{R}	This study
NUS2015	*rpsL1* CPS39 Δ*wcrC*^{39}<>*wcrC*^{10C} // Δ*bgaA*::P$_{Zn}$-*wcrC*^{39} // ΔCEP::P-*kan-wcrB*^{10C}	ΔCEP::P-*kan-wcrB*^{10C} x NUS1480	Kan^{R}	This study
NUS2017	*rpsL1* CPS39 Δ*wcrC*^{39}<>*wcrC*^{33B} // Δ*bgaA*::P$_{Zn}$-*wcrC*^{39}// ΔCEP::P-*kan-wciO*^{33B}	ΔCEP::P-*kan-wciO*^{33B} x NUS1355	Kan^{R} , Str^{R}	This study
NUS2057	*rpsL1* CPS39 Δ*whaI*^{39}<>*wcrO*^{34} // Δ*bgaA*::P$_{Zn}$-*whaI*^{39}	Δ*whaI*^{39}<>*wcrO*^{34} x NUS1463	Str^{R}	This study

NUS2058	*rpsL1* CPS39 Δ*whaI*^{39}<>*wcrB*^{10F} // Δ*bgaA*::P_{Zn}-*whaI*^{39}	Δ*whaI*^{39}<>*wcrB*^{10F} x NUS1463	Str^R	This study
NUS2059	*rpsL1* CPS39 Δ*whaI*^{39}<>*wcrB*^{10B} // Δ*bgaA*::P_{Zn}-*whaI*^{39}	Δ*whaI*^{39}<>*wcrB*^{10B} x NUS1463	Str^R	This study
NUS2060	*rpsL1* CPS39 Δ*whaI*^{39}::P-*erm* Δ*wcrC*^{39}<>*wcrC*^{33B} // Δ*bgaA*::P_{Zn} -*wcrC*^{39} // ΔCEP::P-*kan-wciO*^{33B}	Δ*whaI*^{39}::P-*erm* x NUS2017	Erm^R, Kan^R, Str^R	This study
NUS2061	*rpsL1* CPS39 Δ*whaI*^{39}::P-*erm* Δ*wcrC*^{39}<>*wcrC*^{10C} // Δ*bgaA*::P_{Zn} -*wcrC*^{39} // ΔCEP::P-*kan-wcrB*^{10C}	Δ*whaI*^{39}::P-*erm* x NUS2015	Erm^R, Kan^R, Str^R	This study
NUS2090	*rpsL1* CPS39 Δ*wcrC*^{39}<>*wcrC*^{10A} // Δ*bgaA*::P_{Zn}-wcr*C*^{39} // ΔCEP::P-*kan*-wcr*B*^{10C}	ΔCEP::P-*kan*-wcr*B*^{10C} x NUS1479	Kan^R, Str^R	This study
NUS2148	*rpsL1* CPS39 Δ*wcrC*^{39}<>*wcrC*^{10A} Δ*whaI*^{39}::P-*erm* // Δ*bgaA*::P_{Zn}-*wcrC*^{39} // ΔCEP::P-*kan-wcrB*^{10C}	Δ*whaI*^{39}::P-*erm* x NUS2090	Erm^R, Kan^R, Str^R	This study
NUS2153	*rpsL1* CPS39 Δ*whaI*^{39}<>*wcrO*^{34} // Δ*bgaA*::P-*erm*	Δ*bgaA*::P-*erm* x NUS2057	Str^R, Erm^R	This study
NUS2155	*rpsL1* CPS39 Δ*whaI*^{39}<>*wcrB*^{10B} // Δ*bgaA*::P-*erm*	Δ*bgaA*::P-*erm* x NUS2059	Str^R, Erm^R	This study
NUS2163	*rpsL1* CPS39 Δ*wcrC*^{39}<>*wcrC*^{10A} Δ*whaI*^{39}::P-*erm* // Δ*bgaA*::P-*spec-rpsL*$^{+}$ // ΔCEP::P-*kan-WcrB*^{10C}	Δ*bgaA*::P-*spec-rpsL*$^{+}$ x NUS2148	Erm^R, $Spec^R$, Kan^R	This study
NUS2164	*rpsL1* CPS39 Δ*wcrC*^{39}<>*wcrC*^{10C} // Δ*bgaA*::P_{Zn}-*wcrC*^{39} // ΔCEP::P-*kan-wcrB*^{10A}	ΔCEP::P-*kan-wcrB*^{10A} x NUS1480	Kan^R, Str^R	This study
NUS2187	*rpsL1* CPS39 Δ*wcrC*^{39}<>*wcrC*^{10C} Δ*whaI*^{39}::P-*erm* // Δ*bgaA*::P_{Zn}-*wcrC*^{39} // ΔCEP::P-*kan-wcrB*^{10A}	Δ*whaI*^{39}::P-*erm* x NUS2164	Erm^R, Kan^R, Str^R	This study
NUS2188	*rpsL1* CPS34 Δ*wcrC*^{34}<>*wcrC*^{34}	Δ*wcrC*^{34}<>*wcrC*^{34} ^{S102F} x NUS1969	Str^R	This study

	^{S102F} // Δ*bgaA*::P$_{Zn}$-*wcrC*^{34}			
NUS2189	*rpsL1* CPS47A Δ*wcrC*^{47A}<>*wcrC*^{47A} ^{S95F} // Δ*bgaA*::P$_{Zn}$-*wcrC*^{47A}	Δ*wcrC*^{47A}<>*wcrC*^{47A} ^{S95F} x NUS1970	Str^{R}	This study
NUS2234	*rpsL1* CPS39 Δ*wcrC*^{39}<>*wcrC*^{10C} Δ*whaI*^{39}::P-*erm* // Δ*bgaA*::P-*spec-rpsL*$^{+}$ // ΔCEP::P-*kan-wcrB*^{10A}	Δ*bgaA*::P-*spec-rpsL*$^{+}$ x NUS2187	Str^{R}, Spec^{R}, Kan^{R}	This study
NUS2325	*rpsL1* CPS39 Δ*wcrC*<>*wcrC*^{34}-FLAG // Δ*bgaA*::P$_{Zn}$-*wcrC*^{39}	Δ*wcrC*<>*wcrC*^{34}-FLAG x NUS1303	Str^{R}	This study
NUS2327	*rpsL1* CPS39 Δ*wcrC*<>*wcrC*^{47A}-FLAG // Δ*bgaA*::P$_{Zn}$-*wcrC*^{39}	Δ*wcrC*<>*wcrC*^{47A}-FLAG x NUS1303	Str^{R}	This study
NUS2329	*rpsL1* CPS39 Δ*wcrC*<>*wcrC*^{39}-FLAG // Δ*bgaA*::P$_{Zn}$-*wcrC*^{39}	Δ*wcrC*<>*wcrC*^{39}-FLAG x NUS1303	Str^{R}	This study
NUS2342	*rpsL1* CPS18A Δ*wciU*^{18A}<>*wciU*^{16F}// Δ*bgaA*::P$_{Zn}$-*wciU*^{18A}	Δ*wciU*^{18A}<>*wciU*^{16F} x NUS1968	Str^{R}	This study
NUS2367	*rpsL1* CPS18A Δ*wciU*^{18A}<>*wciU*^{16F}// Δ*bgaA*::P-*erm*	Δ*bgaA*::P-*erm* x NUS2342	Erm^{R}, Str^{R}	This study
NUS2997	*rpsL1* Δ*cps2I-K*::P-*erm* //Δ*bgaA*::P-*kan-cps23BJ*$^{P254S\ T38M}$	Δ*cps2I-K*::P-*erm* x NUS2863	Erm^{R}, Kan^{R}	This study
NUS2998	*rpsL1* Δ*cps2I-K*::P-*erm* // Δ*bgaA*::P-*kan-cps23BJ*$^{P254S\ I246T}$	Δ*cps2I-K*::P-*erm* x NUS2864	Erm^{R}, Kan^{R}	This study
NUS2999	*rpsL1* Δ*cps2I-K*::P-*erm* // Δ*bgaA*::P-*kan-cps23BJ*$^{P254S\ Q276L}$	Δ*cps2I-K*::P-*erm* x NUS2865	Erm^{R}, Kan^{R}	This study
NUS3000	*rpsL1* Δ*cps2I-K*::P-*erm* // Δ*bgaA*::P-*kan-cps23BJ*$^{P254S\ L414P}$	Δ*cps2I-K*::P-*erm* x NUS2866	Erm^{R}, Kan^{R}	This study
NUS3027	*rpsL1* CPS39 Δ*wcrC*<>*wcrC*^{10A}-FLAG // Δ*bgaA*::P$_{Zn}$-*wcrC*^{39}	Δ*wcrC*<>*wcrC*^{10A}-FLAG x NUS1303	Str^{R}	This study
NUS3028	*rpsL1* CPS39 Δ*wcrC*<>*wcrC*^{10C}-FLAG // Δ*bgaA*::P$_{Zn}$-*wcrC*^{39}	Δ*wcrC*<>*wcrC*^{10C}-FLAG x NUS1303	Str^{R}	This study

NUS3059	*rpsL1* Δ*cps2E*::P-*spec-rpsL*$^{+}$ Δ*cps2I-K*::P-*erm* // Δ*bgaA*::P-*kan-cps23BJ*$^{P254S\ T38M}$	Δ*cps2E*::P-*spec-rpsL*$^{+}$ x NUS2997	Erm^{R}, Kan^{R}, Spec^{R}	This study
NUS3060	*rpsL1* Δ*cps2E*::P-*spec-rpsL*$^{+}$ Δ*cps2I-K*::P-*erm* // Δ*bgaA*::P-*kan-cps23BJ*$^{P254S\ I246T}$	Δ*cps2E*::P-*spec-rpsL*$^{+}$ x NUS2998	Erm^{R}, Kan^{R}, Spec^{R}	This study
NUS3061	*rpsL1* Δ*cps2E*::P-*spec-rpsL*$^{+}$ Δ*cps2I-K*::P-*erm* // Δ*bgaA*::P-*kan-cps23BJ*$^{P254S\ Q276L}$	Δ*cps2E*::P-*spec-rpsL*$^{+}$ x NUS2999	Erm^{R}, Kan^{R}, Spec^{R}	This study
NUS3062	*rpsL1* Δ*cps2E*::P-*spec-rpsL*$^{+}$ Δ*cps2I-K*::P-*erm* // Δ*bgaA*::P-*kan-cps23BJ*$^{P254S\ L414P}$	Δ*cps2E*::P-*spec-rpsL*$^{+}$ x NUS3000	Erm^{R}, Kan^{R}, Spec^{R}	This study
NUS3082	*rpsL1* Δ*cps2E* Δ*cps2I-K*::P-*erm* //Δ*bgaA*::P-*kan-cps23BJ*$^{P254S\ T38M}$	Δ*cps2E* x NUS3059	Erm^{R}, Kan^{R}, Str^{R}	This study
NUS3083	*rpsL1* Δ*cps2E* Δ*cps2I-K*::P-*erm*//Δ*bgaA*::P-*kan-cps23BJ*$^{P254S\ I246T}$	Δ*cps2E* x NUS3060	Erm^{R}, Kan^{R}, Str^{R}	This study
NUS3084	*rpsL1* Δ*cps2E* Δ*cps2I-K*::P-*erm* // Δ*bgaA*::P-*kan-cps23BJ*$^{P254S\ Q276L}$	Δ*cps2E* x NUS3061	Erm^{R}, Kan^{R}, Str^{R}	This study
NUS3085	*rpsL1* Δ*cps2E* Δ*cps2I-K*::P-*erm* // Δ*bgaA*::P-*kan-cps23BJ*$^{P254S\ L414P}$	Δ*cps2E* x NUS3062	Erm^{R}, Kan^{R}, Str^{R}	This study
NUS3318	*rpsL1* Δ*cps2I*::P-*erm* Δ*bgaA*::P-*kan-cps23BJ*$^{P254S\ L414P}$ // Δ*ugd*::P-*spec-rpsL*$^{+}$	Δ*ugd*::P-*spec-rpsL*$^{+}$ x NUS2987	Erm^{R}, Kan^{R}, Spec^{R}	This study
NUS3319	*rpsL1* Δ*cps2I-K*::P-*erm* // Δ*bgaA*::P-*kan-cps23BJ*$^{P254S\ L414P}$ // Δ*ugd*::P-*spec-rpsL*$^{+}$	Δ*ugd*::P-*spec-rpsL*$^{+}$ x NUS3000	Erm^{R}, Kan^{R}, Spec^{R}	This study
NUS3362	*rpsL1* Δ*cps2E* Δ*cps2I-K*::P-*erm* // Δ*bgaA*::P-*kan-cps23BJ*$^{P254S\ L414P}$ Δ*ugd*::P-*spec-rpsL*$^{+}$	Δ*ugd*::P-*spec-rpsL*$^{+}$ x NUS3085	Erm^{R}, Kan^{R}, Spec^{R}	This study
NUS3418	*rpsL1* Δ*cps2I*::P-*erm* Δ*bgaA*::P-*kan-cps23BJ*$^{P254S\ L414P}$ // Δ*ugd*	Δ*ugd* x NUS3318	Erm^{R}, Kan^{R}, Str^{R}	This study
NUS3419	*rpsL1* Δ*cps2I-K*::P-*erm* // Δ*bgaA*::P-*kan-cps23BJ*$^{P254S\ L414P}$ // Δ*ugd*	Δ*ugd* x NUS3319	Erm^{R}, Kan^{R}, Str^{R}	This study

NUS3457	*rpsL1* Δ*cps2E*::P-*spec-rpsL*$^{+}$ Δ*cps2I*::P-*erm* Δ*bgaA*::P-*kan-cps23BJ*$^{P254S\ L414P}$ // Δ*ugd*	Δ*cps2E*::P-*spec-rpsL*$^{+}$ x NUS3418	Erm^{R}, Kan^{R}, Spec^{R}	This study
NUS3458	*rpsL1* Δ*cps2E*::P-*spec-rpsL*$^{+}$ Δ*cps2I-K*::P-*erm* // Δ*bgaA*::P-*kan-cps23BJ*$^{P254S\ L414P}$ // Δ*ugd*	Δ*cps2E*::P-*spec-rpsL*$^{+}$ x NUS3419	Erm^{R}, Kan^{R}, Spec^{R}	This study
NUS3528	*rpsL1* CPS39 Δ*wcrC*^{39}<>*wcrC*^{34} ^{S102F} // Δ*bgaA*::P_{Zn}-*wcrC*^{39} // ΔCEP::P-*kan-wcrB*^{10A}	ΔCEP::P-*kan-wcrB*^{10A} x NUS1994	Kan^{R}, Str^{R}	This study
NUS3529	*rpsL1* CPS39 Δ*wcrC*^{39}<>*wcrC*^{35F} ^{S96F} // Δ*bgaA*::P_{Zn}-*wcrC*^{39} // ΔCEP::P-*kan-wcrB*^{10A}	ΔCEP::P-*kan-wcrB*^{10A} *rpsL*+ x NUS1995	Kan^{R}, Str^{R}	This study
NUS3530	*rpsL1* CPS39 Δ*wcrC*^{39}<>*wcrC*^{47F} ^{S95F} // Δ*bgaA*::P_{Zn}-*wcrC*^{39} // ΔCEP::P-*kan-wcrB*^{10A}	ΔCEP::P-*kan-wcrB*^{10A} x NUS1996	Kan^{R}, Str^{R}	This study
NUS3531	*rpsL1* CPS39 Δ*wcrC*^{39}<>*wcrC*$^{47A.S95F}$ // Δ*bgaA*::P_{Zn}-*wcrC*^{39} // ΔCEP::P-*kan-wcrB*^{10A}	ΔCEP::P-*kan-wcrB*^{10A} x NUS1997	Kan^{R}, Str^{R}	This study
NUS3582	*rpsL1* CPS39 Δ*wcrC*^{39}<>*wcrC*^{34} ^{S102F} Δ*whaI*^{39}::P-*erm* // Δ*bgaA*::P_{Zn}-*wcrC*^{39} // ΔCEP::P-*kan-wcrB*^{10A}	Δ*whaI*^{39}::P-*erm* x NUS3528	Erm^{R}, Kan^{R}, Str^{R}	This study
NUS3583	*rpsL1* CPS39 Δ*wcrC*^{39}<>*wcrC*^{35F} ^{S96F} Δ*whaI*^{39}::P-*erm* // Δ*bgaA*::P_{Zn}-*wcrC*^{39} // ΔCEP::P-*kan-wcrB*^{10A}	Δ*whaI*^{39}::P-*erm* x NUS3529	Erm^{R}, Kan^{R}, Str^{R}	This study
NUS3584	*rpsL1* CPS39 Δ*wcrC*^{39}<>*wcrC*^{47F} ^{S95F} Δ*whaI*^{39}::P-*erm* // Δ*bgaA*::P_{Zn}-*wcrC*^{39} // ΔCEP::P-*kan-wcrB*^{10A}	Δ*whaI*^{39}::P-*erm* x NUS3530	Erm^{R}, Kan^{R}, Str^{R}	This study
NUS3585	*rpsL1* CPS39 Δ*wcrC*^{39}<>*wcrC*^{47A} ^{S95F} Δ*whaI*^{39}::P-*erm* // Δ*bgaA*::P_{Zn}-*wcrC*^{39} // ΔCEP::P-*kan-wcrB*^{10A}	Δ*whaI*^{39}::P-*erm* x NUS3531	Erm^{R}, Kan^{R}, Str^{R}	This study

NUS2247	*rpsL1* ΔCEP::P$_{Zn}$-*ytgP*	ΔCEP::P$_{Zn}$-*ytgP* x HMS0019	Str^{R}	This study
NUS1992	*rpsL1* ΔCEP::P$_{Zn}$-*ytgP* Δ*ytgP*::P-*erm*	Δ*ytgP*::P-*erm* x NUS2247	Str^{R}, Erm^{R}, Zn^{2+}	This study
NUS2363	*rpsL1* ΔCEP::P$_{Zn}$-*ytgP* Δ*ytgP*::P-*erm* Δ*bgaA*::P-*kan*-*cps23BJ*$^{P254S\ T38M}$	Δ*bgaA*::P-*kan*-*cps23BJ*$^{P254S\ T38M}$ x NUS1992	Kan^{R}, Str^{R}, Erm^{R}	This study
NUS2364	*rpsL1* ΔCEP::P$_{Zn}$-*ytgP* Δ*ytgP*::P-*erm* Δ*bgaA*::P-*kan*-*cps23BJ*$^{P254S\ I246T}$	Δ*bgaA*::P-*kan*-*cps23BJ*$^{P254S\ I246T}$ x NUS1992	Kan^{R}, Str^{R}, Erm^{R}	This study
NUS2365	*rpsL1* ΔCEP::P$_{Zn}$-*ytgP* Δ*ytgP*::P-*erm* Δ*bgaA*::P-*kan*-*cps23BJ*$^{P254S\ Q276L}$	Δ*bgaA*::P-*kan*-*cps23BJ*$^{P254S\ Q276L}$ x NUS1992	Kan^{R}, Str^{R}, Erm^{R}	This study
NUS2848	*rpsL1* ΔCEP::P$_{Zn}$-*ytgP* Δ*ytgP*::P-*erm* Δ*bgaA*::P-*kan*-*cps23BJ*$^{P254S\ L414P}$	Δ*bgaA*::P-*kan*-*cps23BJ*$^{P254S\ L414P}$ x NUS1992	Kan^{R}, Str^{R}, Erm^{R}	This study
NUS3018	*rpsL1* Δ*bgaA*::P-*kan*-*cps23BJ*-FLAG	Δ*bgaA*::P-*kan*-*cps23BJ*-FLAG x IU1781	Kan^{R}, Str^{R}	This study
NUS3019	*rpsL1* Δ*bgaA*::P-*kan*-*cps23BJ*$^{P254S\ T38M}$ -FLAG	Δ*bgaA*::P-*kan*-*cps23BJ*$^{P254S\ T38M}$ -FLAGx IU1781	Kan^{R}, Str^{R}	This study
NUS3020	*rpsL1* Δ*bgaA*::P-*kan*-*cps23BJ*$^{P254S\ I246T}$-FLAG	Δ*bgaA*::P-*kan*-*cps23BJ*$^{P254S\ I246T}$-FLAG x IU1781	Kan^{R}, Str^{R}	This study
NUS3021	*rpsL1* Δ*bgaA*::P-*kan*-*cps23BJ*$^{P254S\ Q276L}$-FLAG	Δ*bgaA*::P-*kan*-*cps23BJ*$^{P254S\ Q276L}$-FLAG x IU1781	Kan^{R}, Str^{R}	This study
NUS3022	*rpsL1* Δ*bgaA*::P-*kan*-*cps23BJ*$^{P254S\ L414P}$-FLAG	Δ*bgaA*::P-*kan*-*cps23BJ*$^{P254S\ L414P}$-FLAG x IU1781	Kan^{R}, Str^{R}	This study
NUS2064	*rpsL1* CPS19B ΔCEP::P-*kan*-*wchU*^{19C}	ΔCEP::P-*kan*-*wchU*^{19C} x NUS0317	Str^{R}, Kan^{R}	This study
NUS3253	*rpsL1* CPS19C Δ*wchU*^{19C}::P-*erm*	Δ*wchU*^{19C}::P-*erm* x NUS0316	Str^{R}, Erm^{R}	This study
NUS3134	*rpsL1* CPS19C Δ*wchU*^{19C}::P-*erm* // Δ*bgaA*::P-*kan*-*cps23BJ*$^{P254S\ I246T}$	Δ*wchU*^{19C}::P-*erm* x NUS3068	Kan^{R}, Erm^{R}	This study
NUS2841	*rpsL1* CPS14 Δ*bgaA*::P-*kan*-*cps23BJ*$^{P254S\ T38M}$	Δ*bgaA*::P-*kan*-*cps23BJ*$^{P254S\ T38M}$ x NUS0403	Kan^{R}	This study
NUS2842	*rpsL1* CPS14 Δ*bgaA*::P-*kan*-*cps23BJ*$^{P254S\ I246T}$	Δ*bgaA*::P-*kan*-*cps23BJ*$^{P254S\ I246T}$x NUS0403	Kan^{R}	This study

NUS2871	*rpsL1* CPS14 Δ*bgaA*::P-*kan-cps23BJ*$^{P254S\ Q276L}$	Δ*bgaA*::P-*kan-cps23BJ*$^{P254S\ Q276L}$ x NUS0403	Kan^{R}	This study
NUS2843	*rpsL1* CPS14 Δ*bgaA*::P-*kan-cps23BJ*$^{P254S\ L414P}$	Δ*bgaA*::P-*kan-cps23BJ*$^{P254S\ L414P}$ x NUS0403	Kan^{R}	This study
NUS2860	*rpsL1* CPS14 Δ*cps14J*::P-*erm* Δ*bgaA*::P-*kan-cps23BJ*$^{P254S\ T38M}$	Δ*cps14J*::P-*erm* x NUS2841	Kan^{R}, Erm^{R}	This study
NUS2850	*rpsL1* CPS14 Δ*cps14J*::P-*erm* Δ*bgaA*::P-*kan-cps23BJ*$^{P254S\ I246T}$	Δ*cps14J*::P-*erm* x NUS2842	Kan^{R}, Erm^{R}	This study
NUS2904	*rpsL1* CPS14 Δ*cps14J*::P-*erm* Δ*bgaA*::P-*kan-cps23BJ*$^{P254S\ Q276L}$	Δ*cps14J*::P-*erm* x NUS2871	Kan^{R}, Erm^{R}	This study
NUS2861	*rpsL1* CPS14 Δ*cps14J*::P-*erm* Δ*bgaA*::P-*kan-cps23BJ*$^{P254S\ L414P}$	Δ*cps14J*::P-*erm* x NUS2843	Kan^{R}, Erm^{R}	This study
NUS2863	*rpsL1* Δ*bgaA*::P-*kan-cps23BJ*$^{P254S\ T38M}$	Δ*bgaA*::P-*kan-cps23BJ*$^{P254S\ T38M}$ x IU1781	Kan^{R}	This study
NUS2864	*rpsL1* Δ*bgaA*::P-*kan-cps23BJ*$^{P254S\ I246T}$	Δ*bgaA*::P-*kan-cps23BJ*$^{P254S\ I246T}$ x IU1781	Kan^{R}	This study
NUS2865	*rpsL1* Δ*bgaA*::P-*kan-cps23BJ*$^{P254S\ Q276L}$	Δ*bgaA*::P-*kan-cps23BJ*$^{P254S\ Q276L}$ x IU1781	Kan^{R}	This study
NUS2866	*rpsL1* Δ*bgaA*::P-*kan-cps23BJ*$^{P254S\ L414P}$	Δ*bgaA*::P-*kan-cps23BJ*$^{P254S\ L414P}$ x IU1781	Kan^{R}	This study
NUS2867	*rpsL1* CPS23B Δ*bgaA*::P-*kan-cps23BJ*$^{P254S\ T38M}$	Δ*bgaA*::P-*kan-cps23BJ*$^{P254S\ T38M}$ x NUS0329	Kan^{R}	This study
NUS2868	*rpsL1* CPS23B Δ*bgaA*::P-*kan-cps23BJ*$^{P254S\ I246T}$	Δ*bgaA*::P-*kan-cps23BJ*$^{P254S\ I246T}$ x NUS0329	Kan^{R}	This study
NUS2869	*rpsL1* CPS23B Δ*bgaA*::P-*kan-cps23BJ*$^{P254S\ Q276L}$	Δ*bgaA*::P-*kan-cps23BJ*$^{P254S\ Q276L}$x NUS0329	Kan^{R}	This study
NUS2870	*rpsL1* CPS23B Δ*bgaA*::P-*kan-cps23BJ*$^{P254S\ L414P}$	Δ*bgaA*::P-*kan-cps23BJ*$^{P254S\ L414P}$ x NUS0329	Kan^{R}	This study
NUS2906	*rpsL1* CPS23B Δ*cps23BJ*::P-*erm* Δ*bgaA*::P-*kan-cps23BJ*$^{P254S\ T38M}$	Δ*cps23BJ*::P-*erm* x NUS2867	Kan^{R}, Erm^{R}	This study

NUS2907	*rpsL1* CPS23B Δ*cps23BJ*::P-*erm* Δ*bgaA*::P-*kan-cps23BJ*$^{P254S\ I246T}$	Δ*cps23BJ*::P-*erm* x NUS2868	Kan^R, Erm^R	This study
NUS2908	*rpsL1* CPS23B Δ*cps23BJ*::P-*erm* Δ*bgaA*::P-*kan-cps23BJ*$^{P254S\ Q276L}$	Δ*cps23BJ*::P-*erm* x NUS2869	Kan^R, Erm^R	This study
NUS2909	*rpsL1* CPS23B Δ*cps23BJ*::P-*erm* Δ*bgaA*::P-*kan-cps23BJ*$^{P254S\ L414P}$	Δ*cps23BJ*::P-*erm* x NUS2870	Kan^R, Erm^R	This study
NUS2914	*rpsL1* Δ*cps2J*::P-*erm* Δ*bgaA*::P-*kan-cps23BJ*$^{P254S\ T38M}$	Δ*cps2J*::P-*erm* x NUS2863	Kan^R, Erm^R	This study
NUS2915	*rpsL1* Δ*cps2J*::P-*erm* Δ*bgaA*::P-*kan-cps23BJ*$^{P254S\ I246T}$	Δ*cps2J*::P-*erm* x NUS2864	Kan^R, Erm^R	This study
NUS2916	*rpsL1* Δ*cps2J*::P-*erm* Δ*bgaA*::P-*kan-cps23BJ*$^{P254S\ Q276L}$	Δ*cps2J*::P-*erm* x NUS2865	Kan^R, Erm^R	This study
NUS2917	*rpsL1* Δ*cps2J*::P-*erm* Δ*bgaA*::P-*kan-cps23BJ*$^{P254S\ L414P}$	Δ*cps2J*::P-*erm* x NUS2866	Kan^R, Erm^R	This study
NUS2984	*rpsL1* Δ*cps2I*::P-*erm* Δ*bgaA*::P-*kan-cps23BJ*$^{P254S\ T38M}$	Δ*cps2I*::P-*erm* x NUS2863	Kan^R, Erm^R	This study
NUS2985	*rpsL1* Δ*cps2I*::P-*erm* Δ*bgaA*::P-*kan-cps23BJ*$^{P254S\ I246T}$	Δ*cps2I*::P-*erm* x NUS2864	Kan^R, Erm^R	This study
NUS2986	rps*L1* Δ*cps2I*::P-*erm* Δ*bgaA*::P-*kan-cps23BJ*$^{P254S\ Q276L}$	Δ*cps2I*::P-*erm* x NUS2865	Kan^R, Erm^R	This study
NUS2987	*rpsL1* Δ*cps2I*::P-*erm* Δ*bgaA*::P-*kan-cps23BJ*$^{P254S\ L414P}$	Δ*cps2I*::P-*erm* x NUS2866	Kan^R, Erm^R	This study
NUS3038	*rpsL1* CPS14 Δ*cps14E*::P-*sacB-kan-rpsL*$^+$	Δ*cps14E*::P-*sacB-kan-rpsL*$^+$ x NUS0403	Kan^R	This study
NUS3278	*rpsL1* CPS14 Δ*cps14E*	Δ*cps14E* x NUS3038	Str^R	This study
NUS0087	*rpsL1* Δ*cps2J*<>*wzxC*-FLAG^{A33V} // Δ*bgaA*::P$_{Zn}$-*cps2J*	Δ*cps2J*<>*wzxC*-FLAG^{A33V} x NUS0064	Str^R	This study
NUS3266	*rpsL1* Δ*cps2J*<>*wzk* // Δ*bgaA*::P$_{Zn}$-*cps2J*	Δ*cps2J*<>*wzk* x NUS0064	Str^R	This study
NUS2260	*rpsL1* CPS14 Δ*bgaA*::P-*sacB-kan-rpsL*$^+$	Δ*bgaA*::P-*sacB-kan-rpsL*$^+$ x NUS0403	Kan^R, Suc^S Str^S	This study

NUS2107	*rpsL1* CPS14 Δ*bgaA*::P$_{spxB}$-*cps23BJ*^{P254S}	Δ*bgaA*::P$_{spxB}$-*cps23BJ*^{P254S} x NUS2260	Str^{R}	This study
NUS2108	*rpsL1* CPS14 Δ*bgaA*::P$_{spxB}$-*cps23BJ*^{D231G}	Δ*bgaA*::P$_{spxB}$-*cps23BJ*^{D231G} x NUS2260	Str^{R}	This study
LEMO21(IDE3)/pCS190	*fhuA2 lon ompT gal λDE3 dcm* Δ*hsdS* / pLemo	pCS190 x LEMO21	Amp^{R}, Cm^{R}	This study
NUS3213	*rpsL1* CPS5 Δ*bgaA*::P-*kan*-*cps23BJ*$^{P254S\ I246T}$	Δ*bgaA*::P-*kan*-*cps23BJ*$^{P254S\ I246T}$ x NUS0327	Str^{R}, Kan^{R}	This study
NUS3214	*rpsL1* CPS7A Δ*bgaA*::P-*kan*-*cps23BJ*$^{P254S\ I246T}$	Δ*bgaA*::P-*kan*-*cps23BJ*$^{P254S\ I246T}$ x NUS0667	Str^{R}, Kan^{R}	This study
NUS3217	*rpsL1* CPS7F Δ*bgaA*::P-*kan*-*cps23BJ*$^{P254S\ I246T}$	Δ*bgaA*::P-*kan*-*cps23BJ*$^{P254S\ I246T}$ x NUS0310	Str^{R}, Kan^{R}	This study
NUS3231	*rpsL1* CPS10C Δ*bgaA*::P-*kan*-*cps23BJ*$^{P254S\ I246T}$	Δ*bgaA*::P-*kan*-*cps23BJ*$^{P254S\ I246T}$ x NUS0315	Str^{R}, Kan^{R}	This study
NUS3232	*rpsL1* CPS10F Δ*bgaA*::P-*kan*-*cps23BJ*$^{P254S\ I246T}$	Δ*bgaA*::P-*kan*-*cps23BJ*$^{P254S\ I246T}$ x NUS0280	Str^{R}, Kan^{R}	This study
NUS3238	*rpsL1* CPS15A Δ*bgaA*::P-*kan*-*cps23BJ*$^{P254S\ I246T}$	Δ*bgaA*::P-*kan*-*cps23BJ*$^{P254S\ I246T}$ x NUS0999	Str^{R}, Kan^{R}	This study
NUS3069	*rpsL1* CPS15B Δ*bgaA*::P-*kan*-*cps23BJ*$^{P254S\ I246T}$	Δ*bgaA*::P-*kan*-*cps23BJ*$^{P254S\ I246T}$ x NUS0340	Str^{R}, Kan^{R}	This study
NUS3070	*rpsL1* CPS15C Δ*bgaA*::P-*kan*-*cps23BJ*$^{P254S\ I246T}$	Δ*bgaA*::P-*kan*-*cps23BJ*$^{P254S\ I246T}$ x NUS0289	Str^{R}, Kan^{R}	This study
NUS3218	*rpsL1* CPS16F Δ*bgaA*::P-*kan*-*cps23BJ*$^{P254S\ I246T}$	Δ*bgaA*::P-*kan*-*cps23BJ*$^{P254S\ I246T}$ x NUS0292	Str^{R}, Kan^{R}	This study
NUS3092	*rpsL1* CPS18B Δ*bgaA*::P-*kan*-*cps23BJ*$^{P254S\ I246T}$	Δ*bgaA*::P-*kan*-*cps23BJ*$^{P254S\ I246T}$ x NUS0330	Str^{R}, Kan^{R}	This study
NUS3068	*rpsL1* CPS19C Δ*bgaA*::P-*kan*-*cps23BJ*$^{P254S\ I246T}$	Δ*bgaA*::P-*kan*-*cps23BJ*$^{P254S\ I246T}$ x NUS0316	Str^{R}, Kan^{R}	This study
NUS3220	*rpsL1* CPS23A Δ*bgaA*::P-*kan*-*cps23BJ*$^{P254S\ I246T}$	Δ*bgaA*::P-*kan*-*cps23BJ*$^{P254S\ I246T}$ x NUS0399	Str^{R}, Kan^{R}	This study
NUS3222	*rpsL1* CPS23F Δ*bgaA*::P-*kan*-*cps23BJ*$^{P254S\ I246T}$	Δ*bgaA*::P-*kan*-*cps23BJ*$^{P254S\ I246T}$ x NUS0389	Str^{R}, Kan^{R}	This study

NUS3224	*rpsL1* CPS24B Δ*bgaA*::P-*kan-cps23BJ*$^{P254S\ I246T}$	Δ*bgaA*::P-*kan-cps23BJ*$^{P254S\ I246T}$ x NUS0247	Str^R, Kan^R	This study
NUS3225	*rpsL1* CPS24F Δ*bgaA*::P-*kan-cps23BJ*$^{P254S\ I246T}$	Δ*bgaA*::P-*kan-cps23BJ*$^{P254S\ I246T}$ x NUS0311	Str^R, Kan^R	This study
NUS3226	*rpsL1* CPS27 Δ*bgaA*::P-*kan-cps23BJ*$^{P254S\ I246T}$	Δ*bgaA*::P-*kan-cps23BJ*$^{P254S\ I246T}$ x NUS0463	Str^R, Kan^R	This study
NUS3247	*rpsL1* CPS28A Δ*bgaA*::P-*kan-cps23BJ*$^{P254S\ I246T}$	Δ*bgaA*::P-*kan-cps23BJ*$^{P254S\ I246T}$ x NUS0671	Str^R, Kan^R	This study
NUS3241	*rpsL1* CPS28F Δ*bgaA*::P-*kan-cps23BJ*$^{P254S\ I246T}$	Δ*bgaA*::P-*kan-cps23BJ*$^{P254S\ I246T}$ x NUS0390	Str^R, Kan^R	This study
NUS3228	*rpsL1* CPS32F Δ*bgaA*::P-*kan-cps23BJ*$^{P254S\ I246T}$	Δ*bgaA*::P-*kan-cps23BJ*$^{P254S\ I246T}$ x NUS0342	Str^R, Kan^R	This study
NUS3242	*rpsL1* CPS33A Δ*bgaA*::P-*kan-cps23BJ*$^{P254S\ I246T}$	Δ*bgaA*::P-*kan-cps23BJ*$^{P254S\ I246T}$ x NUS0332	Str^R, Kan^R	This study
NUS3250	*rpsL1* CPS33D Δ*bgaA*::P-*kan-cps23BJ*$^{P254S\ I246T}$	Δ*bgaA*::P-*kan-cps23BJ*$^{P254S\ I246T}$ x NUS0672	Str^R, Kan^R	This study
NUS3251	*rpsL1* CPS33F Δ*bgaA*::P-*kan-cps23BJ*$^{P254S\ I246T}$	Δ*bgaA*::P-*kan-cps23BJ*$^{P254S\ I246T}$ x NUS0326	Str^R, Kan^R	This study
NUS3252	*rpsL1* CPS35C Δ*bgaA*::P-*kan-cps23BJ*$^{P254S\ I246T}$	Δ*bgaA*::P-*kan-cps23BJ*$^{P254S\ I246T}$ x NUS0369	Str^R, Kan^R	This study
NUS3095	*rpsL1* CPS41A Δ*bgaA*::P-*kan-cps23BJ*$^{P254S\ I246T}$	Δ*bgaA*::P-*kan-cps23BJ*$^{P254S\ I246T}$ x NUS0374	Str^R, Kan^R	This study
NUS3922	*rpsL1* CPS10C Δ*wcrH*^{10C}::P-*erm* Δ*bgaA::P-kan-cps23BJ*$^{P254S\ I246T}$	Δ*wcrH*^{10C}::P-*erm* x NUS3231	Str^R, Erm^R, Kan^R	This study
NUS3887	*rpsL1* CPS10F Δ*wcrH*^{10F}::P-*erm* Δ*bgaA::P-kan-cps23BJ*$^{P254S\ I246T}$	Δ*wcrH*^{10F}::P-*erm* x NUS3232	Str^R, Erm^R, Kan^R	This study
NUS3888	*rpsL1* CPS15B Δ*wchN*^{15B}::P-*erm* Δ*bgaA::P-kan-cps23BJ*$^{P254S\ I246T}$	Δ*wchN*^{15B}::P-*erm* x NUS3069	Str^R, Erm^R, Kan^R	This study
NUS3889	*rpsL1* CPS15B Δ*wchX*^{15B}::P-*erm* Δ*bgaA::P-kan-cps23BJ*$^{P254S\ I246T}$	Δ*wchX*^{15B}::P-*erm* x NUS3069	Str^R, Erm^R, Kan^R	This study

NUS3890	*rpsL1* CPS15C Δ*wchN*^{15C}::P-*erm* Δ*bgaA::P-kan-cps23BJ*$^{P254S\ I246T}$	Δ*wchN*^{15C}::P-*erm* x NUS3070	Str^R, Erm^R, Kan^R	This study
NUS3917	*rpsL1* CPS15C Δ*wchX*^{15C}::P-*erm* Δ*bgaA::P-kan-cps23BJ*$^{P254S\ I246T}$	Δ*wchX*^{15C}::P-*erm* x NUS3070	Str^R, Erm^R, Kan^R	This study
NUS3912	*rpsL1* CPS16F Δ*wcxP*^{16F}::P-*erm* Δ*bgaA::P-kan-cps23BJ*$^{P254S\ I246T}$	Δ*wcxP*^{16F}::P-*erm* x NUS3218	Str^R, Erm^R, Kan^R	This study
NUS3913	*rpsL1* CPS28A Δ*wcxP*^{28A}::P-*erm* Δ*bgaA::P-kan-cps23BJ*$^{P254S\ I246T}$	Δ*wcxP*^{28A}::P-*erm* x NUS3247	Str^R, Erm^R, Kan^R	This study
NUS3914	*rpsL1* CPS28A Δ*wcxQ*^{28A}::P-*erm* Δ*bgaA::P-kan-cps23BJ*$^{P254S\ I246T}$	Δ*wcxQ*^{28A}::P-*erm* x NUS3247	Str^R, Erm^R, Kan^R	This study

[a] Strains were constructed as described in Methods. "::" indicates an insertion into a gene and "<>" represents a complete open reading frame replacement. Unless otherwise specified, deletion mutants retain 60 base pairs 5' and 3' of the corresponding open reading frames to avoid polarity. Capsule-switch mutants of the indicated serotype are abbreviated for convenience. For example, "CPS23B" is referring to the serotype 23B capsule-switch mutant.

[b] Primers used to synthesize fusion amplicons are listed in **Table S12**. The amino acid sequence of the FLAG epitope is DYKDDDDK.

[c] Antibiotic resistance markers: Amp^R, ampicillin; Erm^R, erythromycin; Kan^R, kanamycin; $Spec^R$, spectinomycin; Str^R, streptomycin; Suc^S, sucrose sensitive (*sacB*$^+$).

Table S12. Oligonucleotides used in this study

Primer	Sequence (5' to 3')	Template	Amplicon
For construction of Δ*cps*::P-*sacB-kan-rpsL*$^+$			
P15	GCGCATAGTGGATGGTAGTT	D39	*cps 5'*
P16	CATTATCCATTAAAAATCAAACGGATCCTAGATTAACACCTATACATTGAACATCTTACG		
P1	TAGGATCCGTTTGATTTTTAATGGATAATG	P-*kan-rpsL*$^+$ cassette	P-*kan-rpsL*$^+$
P2	GGGCCCCTTTCCTTATGCTTTTG		
P17	CGTCCAAAAGCATAAGGAAAGGGGCCCTTTGTTATTCCAACTTGGCAAGATGCATTGC	D39	*cps 3'*
P18	GGAAACAGCTAAACGTCATCAAC		
For construction of Δ*bgaA*::P-*sacB-kan-rpsL*$^+$			
P23	CCGTAGAACCACTATCACAAG	D39	*bgaA* 5'
P24	TTATCCATTAAAAATCAAACGGATCCTATCCCACAGCAAACTTACGAATGCTATAAACTC		
P1	TAGGATCCGTTTGATTTTTAATGGATAATG	SpnYL001	P-*sacB-kan-rpsL*$^+$
P2	GGGCCCCTTTCCTTATGCTTTTG		
P25	AAAAGCATAAGGAAAGGGGCCCTTAGCTCTTCTAGGTTTGAGTGCAGGATTAGTAGTTAC	D39	*bgaA* 3'
P26	GACGAAACTTTGCGGATTTG		
For construction of Δ*bgaA*::P$_{Zn}$-*wcrC*^{39}			
P86	GTTTGACTGCCGGTGTATCT	AF24	BgaA-PczcD 5'
P147	ATTTCTCATTCCTTTGTTATAATAG		
P1775	CTATTATAACAAAGGAATGAGAAATATGAAGAAAATAGCTTTAGTTAAGTGG	PATH2009	*wcrC*^{39} ORF
P1776	TCATTCTCCCGTCATTTTTTCTATC		
P1777	GATAGAAAAAATGACGGGAGAATGATTAGCTCTTCTAGGTTTGAGTGCAGGATTAG	D39	*bgaA* 3'
P6	TGCATGGTTACGATAGTCTTGG		
For construction of Δ*wcrC*^{39}::P-*sacB-kan-rpsL*$^+$			
P1837	AACTGATAGACCTTGGCATGAA	PATH2009	*wcrC*^{39} 5'
P1838	CATTATCCATTAAAAATCAAACGGATCCTAAACTTTCAAGCCACCATCTGTACCGTC		
P1	TAGGATCCGTTTGATTTTTAATGGATAATG	SpnYL001	P-*sacB-kan-rpsL*$^+$
P2	GGGCCCCTTTCCTTATGCTTTTG		
P1839	CAAAAGCATAAGGAAAGGGGCCCAATAAAAAGAACATTACTAAACAATGGATTG	PATH2009	*wcrC*^{39} 3'
P1840	TGCAGATACCGTAGCAACTTT		
For construction of Δ*wcrC*^{39}<>*wcrC*^{33B}			
P1837	AACTGATAGACCTTGGCATGAA	PATH2009	*wcrC*^{39} 5'
P1981	CTTACTATCGCAATTTTTTTCATTTTTTACCATCATTTCAATTTACTTTTAATTAAG		
P1980	ATGAAAAAAATTGCGATAGTAAG	PATH1945	*wcrC*^{33B} ORF
P1983	CTATTCCCCTGTCATTTCTTC		

P1982	GAAGAAATGACAGGGGAATAGAATGTAC<br>GATAAATTAGTAACAATCATAGTAC	PATH2009	*wcrC*^{39} 3’
P1840	TGCAGATACCGTAGCAACTTT		
For construction of Δ*wcrC*^{39}<>*wcrC*^{33C}			
P1837	AACTGATAGACCTTGGCATGAA	PATH2009	*wcrC*^{39} 5’
P1985	CTCACTATACCTATTTTTTTCATTTTTTACC<br>ATCATTTCAATTTACTTTTAATTAAG		
P1984	ATGAAAAAAATAGGTATAGTGAG	PATH334	*wcrC*^{33C} ORF
P1987	TCATTCTCCCGTCATTTCTTCTAT		
P1986	ATAGAAGAAATGACGGGAGAATGAAATGT<br>ACGATAAATTAGTAACAATCATAG	PATH2009	*wcrC*^{39} 3’
P1840	TGCAGATACCGTAGCAACTTT		
For construction of Δ*wcrC*^{39}<>*wcrC*^{47A}			
P1837	AACTGATAGACCTTGGCATGAA	PATH2009	*wcrC*^{39} 5’
P1989	CACTAAAGCGATTTTTTTCATTTTTTACCA<br>TCATTTCAATTTACTTTTAATTAAG		
P1988	ATGAAAAAAATCGCTTTAGTG	PATH2476	*wcrC*^{47A} ORF
P1991	TTAACGATTATCCGCTCCTATTTC		
P1990	GAAATAGGAGCGGATAATCGTTAAAATGT<br>ACGATAAATTAGTAACAATCATAG	PATH2009	*wcrC*^{39} 3’
P1840	TGCAGATACCGTAGCAACTTT		
For construction of Δ*wcjH*^{39}::P-*erm*			
P1996	CCAGAAGAAGCTAGCCGTATC	PATH2009	*wcrC*^{39} 5’
P1997	CATTATCCATTAAAAATCAAACGGATCCT<br>ATAGGAAAAACGCCAAAAATCTATC		
P1	TAGGATCCGTTTGATTTTTAATGGATAATG	P-*erm* cassette	P-*erm*
P2	GGGCCCCTTTCCTTATGCTTTTG		
P1998	CAAAAGCATAAGGAAAGGGGCCCACTGAT<br>TTCTTGCTTATTTTGAAGGG	PATH2009	*wcrC*^{39} 3’
P1999	GGTTCCACCCAAGGCATAATA		
For construction of Δ*wcrC*^{39}<>*wcrC*^{33D}			
P1837	AACTGATAGACCTTGGCATGAA	PATH2009	*wcrC*^{39} 5’
P1981	CTTACTATCGCAATTTTTTTCATTTTTTACC<br>ATCATTTCAATTTACTTTTAATTAAG		
P1980	ATGAAAAAAATTGCGATAGTAAG	PATH2481	*wcrC*^{33D} ORF
P1983	CTATTCCCCTGTCATTTCTTC		
P1982	GAAGAAATGACAGGGGAATAGAATGTAC<br>GATAAATTAGTAACAATCATAGTAC	PATH2009	*wcrC*^{39} 3’
P1840	TGCAGATACCGTAGCAACTTT		
For construction of Δ*wcrC*^{39}<>*wcrF*^{10F}			
P1837	AACTGATAGACCTTGGCATGAA	PATH2009	*wcrC*^{39} 5’
P2004	CTAACAATTGCTATTTTTTTCATTTTTTACC<br>ATCATTTCAATTTACTTTTAATTAAG		
P1889	ATGAAAAAAATAGCAATTGTTAG	PATH1539	*wcrC*^{10F} ORF
P1890	CTAAATTGTCTCAATTAATTCTATC		
P2005	GATAGAATTAATTGAGACAATTTAGAATG<br>TACGATAAATTAGTAACAATC	PATH2009	*wcrC*^{39} 3’
P1840	TGCAGATACCGTAGCAACTTT		
For construction of Δ*wcrC*^{39}<>*wcrF*^{10B}			

P1837	AACTGATAGACCTTGGCATGAA	PATH2009	*wcrC*^{39} 5'
P2004	CTAACAATTGCTATTTTTTTCATTTTTTACC ATCATTTCAATTTACTTTTAATTAAG		
P1889	ATGAAAAAAATAGCAATTGTTAG	PATH2459	*wcrC*^{10B} ORF
P2248	CTAAATTGTCTCAATCAACTCTATC		
P2249	GATAGAGTTGATTGAGACAATTTAGAATG TACGATAAATTAGTAA	PATH2009	*wcrC*^{39} 3'
P1840	TGCAGATACCGTAGCAACTTT		
For construction of Δ*bgaA*::P-*erm*			
P23	CCGTAGAACCACTATCACAAG	D39	*bgaA* 5'
P24	TTATCCATTAAAAATCAAACGGATCCTATC CCACAGCAAACTTACGAATGCTATAAACT C		
P1	TAGGATCCGTTTGATTTTTAATGGATAATG	P-*erm* cassette	P-*erm*
P2	GGGCCCCTTTCCTTATGCTTTTG		
P25	AAAAGCATAAGGAAAGGGGCCCTTAGCTC TTCTAGGTTTGAGTGCAGGATTAGTAGTTA C	D39	*bgaA* 3'
P26	GACGAAACTTTGCGGATTTG		
For construction of Δ*wcrC*^{39}<>*wcrC*^{34}			
P1837	AACTGATAGACCTTGGCATGAA	PATH2009	*wcrC*^{39} 5'
P2074	TTTTTACCATCATTTCAATTTAC		
P2076	CTTAATTAAAAGTAAATTGAAATGATGGT AAAAAGTGAAGGGTAGGATAGAAATTG	PATH1748	*wcrC*^{34} ORF
P2077	CTATGATTGTTACTAATTTATCGTACATTT TAGACATTTCCCCCCCAGATAC		
P2075	AATGTACGATAAATTAGTAACAATC	PATH2009	*wcrC*^{39} 3'
P1840	TGCAGATACCGTAGCAACTTT		
For construction of Δ*wcrC*^{39}<>*wcrC*^{35F}			
P1837	AACTGATAGACCTTGGCATGAA	PATH2009	*wcrC*^{39} 5'
P2074	TTTTTACCATCATTTCAATTTAC		
P2084	GTAAATTGAAATGATGGTAAAAATTGGGA AAAAAAATATGTATTGTGAAATG	PATH676	*wcrC*^{35F} ORF
P2085	GATTGTTACTAATTTATCGTACATTTTAGA CATTTCCCCCCCCAGATACTTC		
P2075	AATGTACGATAAATTAGTAACAATC	PATH2009	*wcrC*^{39} 3'
P1840	TGCAGATACCGTAGCAACTTT		
For construction of Δ*wcrC*^{39}<>*wcrC*^{47F}			
P1837	AACTGATAGACCTTGGCATGAA	PATH2009	*wcrC*^{39} 5'
P2074	TTTTTACCATCATTTCAATTTAC		
P2078	GTAAATTGAAATGATGGTAAAAAATGAAA AAAATCGCTTTAGTGAAATGG	PATH2475	*wcrC*^{47F} ORF
P2079	GATTGTTACTAATTTATCGTACATTTTAGA CATTTCCCCCCCCAGATACTTC		
P2075	AATGTACGATAAATTAGTAACAATC	PATH2009	*wcrC*^{39} 3'
P1840	TGCAGATACCGTAGCAACTTT		
For construction of Δ*bgaA*::P_{Zn}-*whaI*^{39}			
P86	GTTTGACTGCCGGTGTATCT	AF24	*bgaA*-P_{czcD} 5'
P147	ATTTCTCATTCCTTTGTTATAATAG		

P2096	CTATTATAACAAAGGAATGAGAAATATGA AATCAGAGATAGAAATTATTCAG	PATH2009	*whaI*^{39} ORF
P2097	TTACTCACCTATATTCTCTCCTAAAC		
P2098	GTTTAGGAGAGAATATAGGTGAGTAATTA GCTCTTCTAGGTTTGAGTGCAGG	D39	*bgaA* 3’
P6	TGCATGGTTACGATAGTCTTGG		
For construction of Δ*whaI*^{39}::P-*sacB-kan-rpsL*$^{+}$			
P2080	AGATGCCGATCAGAGAATAAAGG	PATH2009	*whaI*^{39} 5’
P2082	CATTATCCATTAAAAATCAAACGGATCCT ATATAAATAATTTTAACAACTC		
P1	TAGGATCCGTTTGATTTTTAATGGATAATG	SpnYL001	P-*sacB-kan-rpsL*$^{+}$
P2	GGGCCCCTTTCCTTATGCTTTTG		
P2083	CAAAAGCATAAGGAAAGGGGCCCGAACA CAGAGTTCCTCATCAATATAGAGTC	PATH2009	*whaI*^{39} 3’
P2753	CGGATTTGTTGATTCCAATTCCA		
For construction of Δ*wcrC*^{39}<>*wcrC*^{10A}			
P1837	AACTGATAGACCTTGGCATGAA	PATH2009	*wcrC*^{39} 5’
P1969	CATTTCACTAAAGCTATTTTTTTCATTTTTT ACCATCATTTCAATTTAC		
P1940	ATGAAAAAAATAGCTTTAGTGAAATG	PATH691	*wcrC*^{10A} ORF
P1941	CTAAATTGTCTCAATCAACTCTATC		
P1970	GATAGAGTTGATTGAGACAATTTAGAATG TACGATAAATTAGTAACAATCATAG	PATH2009	*wcrC*^{39} 3’
P1840	TGCAGATACCGTAGCAACTTT		
For construction of Δ*wcrC*^{39}<>*wcrC*^{10C}			
P1837	AACTGATAGACCTTGGCATGAA	PATH2009	*wcrC*^{39} 5’
P1969	CATTTCACTAAAGCTATTTTTTTCATTTTTT ACCATCATTTCAATTTAC		
P1940	ATGAAAAAAATAGCTTTAGTGAAATG	PATH2460	*wcrC*^{10C} ORF
P1941	CTAAATTGTCTCAATCAACTCTATC		
P1970	GATAGAGTTGATTGAGACAATTTAGAATG TACGATAAATTAGTAACAATCATAG	PATH2009	*wcrC*^{39} 3’
P1840	TGCAGATACCGTAGCAACTTT		
For construction of ΔCEP::P-*kan-rpsL*$^{+}$			
P3	ATTTGCCCATCTGGCTGACTAGGAGGAA	IU5122	ΔCEP::P-*kan-rpsL*
P4	TGGGCACGTCATTTCCAATGATTACCTG		
For construction of Δ*wcrC*^{39}			
P1837	AACTGATAGACCTTGGCATGAA	PATH2009	*wcrC*^{39} 5’
P2162	GTTACTAATTTATCGTACATTTTTTTACCAT CATTTCAATTTAC		
P2161	GTAAATTGAAATGATGGTAAAAAAATGTA CGATAAATTAGTAAC	PATH2009	*wcrC*^{39} 3’
P1840	TGCAGATACCGTAGCAACTTT		
For construction of ΔCEP::P-*kan-wcrB*^{10A}			
P3	ATTTGCCCATCTGGCTGACTAGGAGGAA	IU5122	CEP 5’
P2398	CAAGAAATAAAATAATAGCACCTACATTC TCCTGTGTTTTTTTA		
P2395	GTGCTATTATTTTATTTCTTGGAG	PATH691	*wcrB*^{10A} ORF
P2389	TTAGTCGCTAGGGAAGGAGATTTC		

P2396	CTCCTTCCCTAGCGACTAACAAAAGCATA AGGAAAGGGGCCC	IU5122	CEP 3’
P4	TGGGCACGTCATTTCCAATGATTACCTG		
For construction of ΔCEP::P-*kan-wciO*^{33D}			
P3	ATTTGCCCATCTGGCTGACTAGGAGGAA	IU5122	CEP 5’
P2397	CTATACTTTTAACACTTACCATCTACATTC TCCTGTGTTTTTTA		
P2392	ATGGTAAGTGTTAAAAGTATAGAAC	PATH2481	*wciO*^{33D} ORF
P2387	TTATTTTACTATCTTTTTAATAGAG		
P2393	CTATTAAAAAGATAGTAAAATAACAAAAG CATAAGGAAAGGGGCC	IU5122	CEP 3’
P4	TGGGCACGTCATTTCCAATGATTACCTG		
For construction of Δ*wcrB*^{10A}::P-*erm*			
P2250	GCGCTATATTCCTCATCCTATGG	PATH691	*wcrB*^{10A} 5’
P2252	CCATTAAAAATCAAACGGATCCTATTTAA ACAATGTTACTGTAG		
P1	TAGGATCCGTTTGATTTTTAATGGATAATG	P-*erm* cassette	P-*erm*
P2	GGGCCCCTTTCCTTATGCTTTTG		
P2253	CAAAAGCATAAGGAAAGGGGCCCCCTGCA GTTGAAGATAGGG	PATH691	*wcrB*^{10A} 3’
P2251	CCGTTGGACATCTGAAGCTAA		
For construction of Δ*wciO*^{33D}::P-*erm*			
P2254	ATGGTTGGACAATCTGGAAAGA	PATH2481	*wciO*^{33D} 5’
P2256	CCATTAAAAATCAAACGGATCCTATGCTTC CCTTTTCTTTTCTTC		
P1	TAGGATCCGTTTGATTTTTAATGGATAATG	P-*erm* cassette	P-*erm*
P2	GGGCCCCTTTCCTTATGCTTTTG		
P2257	CAAAAGCATAAGGAAAGGGGCCCGTTTAT TTCCTCATTAATTTAT	PATH2481	*wciO*^{33D} 3’
P2255	GATGGACCCGTTGGACATTTA		
For construction of Δ*wcrC*^{39}<>*wcrC*$^{34\ D129A}$			
P1837	AACTGATAGACCTTGGCATGAA	NUS1429	*wcrC*^{34} $_{D129A}$ 5’
P2259	CTGAGAAAATTCTCTATTAGCCGTAATCGA ATTGGTATG		
P2258	CATACCAATTCGATTACGGCTAATAGAGA ATTTTCTCAG	NUS1429	*wcrC*^{34} $_{D129A}$ 3’
P1840	TGCAGATACCGTAGCAACTTT		
For construction of Δ*wcrC*^{39}<>*wcrC*$^{35F\ D123A}$			
P1837	AACTGATAGACCTTGGCATGAA	NUS1465	*wcrC*^{35F} $_{D123A}$ 5’
P2259	CTGAGAAAATTCTCTATTAGCCGTAATCGA ATTGGTATG		
P2258	CATACCAATTCGATTACGGCTAATAGAGA ATTTTCTCAG	NUS1465	*wcrC*^{35F} $_{D123A}$ 3’
P1840	TGCAGATACCGTAGCAACTTT		
For construction of Δ*wcrC*^{39}<>*wcrC*$^{47F\ D122A}$			
P1837	AACTGATAGACCTTGGCATGAA	NUS1466	*wcrC*^{47F} $_{D122A}$ 5’
P2261	CTGATAAAATTCTCTATTAGCCGTAATCGA ATTGGTATG		

P2260	CATACCAATTCGATTACGGCTAATAGAGA ATTTTATCAG	NUS1466	*wcrC*$^{47F}_{D122A}$ 3’
P1840	TGCAGATACCGTAGCAACTTT		
For construction of Δ*wcrC*^{39}<>*wcrC*$^{47A\ D122A}$			
P1837	AACTGATAGACCTTGGCATGAA	NUS1410	*wcrC*$^{47A}_{D122A}$ 5’
P2263	CTGAGAAAACTCTCGATTAGCTGTAATTG AATTCGTATG		
P2262	CATACGAATTCAATTACAGCTAATCGAGA GTTTTCTCAG	NUS1410	*wcrC*$^{47A}_{D122A}$ 3’
P1840	TGCAGATACCGTAGCAACTTT		
For construction of Δ*wcrC*^{39}<>*wcrC*$^{34\ S102F}$			
P1837	AACTGATAGACCTTGGCATGAA	NUS1429	*wcrC*$^{34}_{S102F}$ 5’
P2446	GATAAAAATACATTAGCGAATATCCCTAT CCCAAAACC		
P2445	GGTTTTGGGATAGGGATATTCGCTAATGTA TTTTTATC	NUS1429	*wcrC*$^{34}_{S102F}$ 3’
P2086	ACGTCTCTGACTACTGGTTCT		
For construction of Δ*wcrC*^{39}<>*wcrC*$^{35F\ S96F}$			
P1837	AACTGATAGACCTTGGCATGAA	NUS1465	*wcrC*$^{35F}_{S96F}$ 5’
P2446	GATAAAAATACATTAGCGAATATCCCTAT CCCAAAACC		
P2445	GGTTTTGGGATAGGGATATTCGCTAATGTA TTTTTATC	NUS1465	*wcrC*$^{35F}_{S96F}$ 3’
P2086	ACGTCTCTGACTACTGGTTCT		
For construction of Δ*wcrC*^{39}<>*wcrC*$^{47F\ S95F}$			
P1837	AACTGATAGACCTTGGCATGAA	NUS1466	*wcrC*$^{47F}_{S95F}$ 5’
P2448	GATAAAAATACATTAGCGAATATCCCTAT TCCAAAGGC		
P2447	GCCTTTGGAATAGGGATATTCGCTAATGTA TTTTTATC	NUS1466	*wcrC*$^{47F}_{S95F}$ 3’
P2086	ACGTCTCTGACTACTGGTTCT		
For construction of Δ*wcrC*^{39}<>*wcrC*$^{47A\ S95F}$			
P1837	AACTGATAGACCTTGGCATGAA	NUS1410	*wcrC*$^{47A}_{S95F}$ 5’
P2450	GATAAAAATATATTTGCGAATATTCCTATT CCAAAGGC		
P2449	GCCTTTGGAATAGGAATATTCGCAAATAT ATTTTTATC	NUS1410	*wcrC*$^{47A}_{S95F}$ 3’
P2086	ACGTCTCTGACTACTGGTTCT		
For construction of Δ*wcrC*^{39}<>*wcrC*$^{10A\ F95S}$			
P1837	AACTGATAGACCTTGGCATGAA	NUS1479	*wcrC*$^{10A}_{F95S}$ 5’
P2508	GATAAAAATATATTAGAAGATATCCCTAT CCCAAAG		
P2507	CTTTGGGATAGGGATATCTTCTAATATATT TTTATC	NUS1479	*wcrC*$^{10A}_{F95S}$ 3’
P1840	TGCAGATACCGTAGCAACTTT		
For construction of Δ*wcrC*^{39}<>*wcrC*$^{10C\ F95S}$			
P1837	AACTGATAGACCTTGGCATGAA	NUS1480	*wcrC*$^{10C}_{F95S}$ 5’
P2510	GATAAAAATACATTAGCAGATATTCCTAT CCCAAAG		

P2509	CTTTGGGATAGGAATATCTGCTAATGTATT TTTATC	NUS1480	*wcrC*$^{10C\ F95S}$ 3’
P1840	TGCAGATACCGTAGCAACTTT		
For construction of Δ*wcrC*^{39}<>*wcrC*^{39}-FLAG			
P1837	AACTGATAGACCTTGGCATGAA	PATH2009	*wcrC*^{39} 5’
O1571	CACTTAACTAAAGCTATTTCTTCATTTTTT ACCATCATTTCAATTTAC		
O1572_	ATGAAGAAAATAGCTTTAGTTAAGTG	PATH2009	*wcrC*^{39}-FLAG ORF
O1573_	TTTGTCATCATCATCTTTATAATCTCATTCT CCCGTCATTTTTTCTATC		
O1393	GATTATAAAGATGATGATGACAAAAATGT ACGATAAATTAGTAACAATCATAG	PATH2009	*wcrC*^{39} 3’
P1840	TGCAGATACCGTAGCAACTTT		
For construction of Δ*wcrC*^{39}<>*wcrC*^{10A}-FLAG			
P1837	AACTGATAGACCTTGGCATGAA	PATH2009	*wcrC*^{39} 5’
P1969	CATTTCACTAAAGCTATTTTTTTCATTTTTT ACCATCATTTCAATTTAC		
P1940	ATGAAAAAAATAGCTTTAGTGAAATG	PATH691	*wcrC*^{10A}-FLAG ORF
O1387_	TTTGTCATCATCATCTTTATAATCCTAAAT TGTCTCAATCAACTCTATC		
O1393	GATTATAAAGATGATGATGACAAAAATGT ACGATAAATTAGTAACAATCATAG	PATH2009	*wcrC*^{39} 3’
P1840	TGCAGATACCGTAGCAACTTT		
For construction of Δ*wcrC*^{39}<>*wcrC*^{10C}-FLAG			
P1837	AACTGATAGACCTTGGCATGAA	PATH2009	*wcrC*^{39} 5’
P1969	CATTTCACTAAAGCTATTTTTTTCATTTTTT ACCATCATTTCAATTTAC		
P1940	ATGAAAAAAATAGCTTTAGTGAAATG	PATH2460	*wcrC*^{10C}-FLAG ORF
O1387_	TTTGTCATCATCATCTTTATAATCCTAAAT TGTCTCAATCAACTCTATC		
O1393	GATTATAAAGATGATGATGACAAAAATGT ACGATAAATTAGTAACAATCATAG	PATH2009	*wcrC*^{39} 3’
P1840	TGCAGATACCGTAGCAACTTT		
For construction of Δ*wcrC*^{39}<>*wcrC*$^{10A\ F95S}$-FLAG			
P1837	AACTGATAGACCTTGGCATGAA	PATH2009	*wcrC*^{39} 5’
P1969	CATTTCACTAAAGCTATTTTTTTCATTTTTT ACCATCATTTCAATTTAC		
P1940	ATGAAAAAAATAGCTTTAGTGAAATG	PATH691	*wcrC*^{10A}-FLAG ORF
O1387_	TTTGTCATCATCATCTTTATAATCCTAAAT TGTCTCAATCAACTCTATC		
O1393	GATTATAAAGATGATGATGACAAAAATGT ACGATAAATTAGTAACAATCATAG	PATH2009	*wcrC*^{39} 3’
P1840	TGCAGATACCGTAGCAACTTT		
For construction of Δ*wcrC*^{39}<>*wcrC*$^{10C\ F95S}$-FLAG			
P1837	AACTGATAGACCTTGGCATGAA	PATH2009	*wcrC*^{39} 5’
P1969	CATTTCACTAAAGCTATTTTTTTCATTTTTT ACCATCATTTCAATTTAC		
P1940	ATGAAAAAAATAGCTTTAGTGAAATG	PATH2460	

O1387_	TTTGTCATCATCATCTTTATAATCCTAAATTGTCTCAATCAACTCTATC		*wcrC*^{10C}-FLAG ORF
O1393	GATTATAAAGATGATGATGACAAAAATGTACGATAAATTAGTAACAATCATAG	PATH2009	*wcrC*^{39} 3’
P1840	TGCAGATACCGTAGCAACTTT		
For construction of Δ*wcrC*^{39}<>*wcrC*^{34}-FLAG			
P1837	AACTGATAGACCTTGGCATGAA	PATH2009	*wcrC*^{39} 5’
P2074	TTTTTACCATCATTTCAATTTAC		
P2076	CTTAATTAAAAGTAAATTGAAATGATGGTAAAAAGTGAAGGGTAGGATAGAAATTG	PATH1748	*wcrC*^{34}-FLAG ORF
O1391_	TTTGTCATCATCATCTTTATAATCTTAGACATTTTCCCCCCCAGATACTTC		
O1393	GATTATAAAGATGATGATGACAAAAATGTACGATAAATTAGTAACAATCATAG	PATH2009	*wcrC*^{39} 3’
P1840	TGCAGATACCGTAGCAACTTT		
For construction of Δ*wcrC*^{39}<>*wcrC*^{34}-FLAG			
P1837	AACTGATAGACCTTGGCATGAA	PATH2009	*wcrC*^{39} 5’
P1989	CACTAAAGCGATTTTTTTCATTTTTACCATCATTTCAATTTACTTTTAATTAAG		
P1988	ATGAAAAAAATCGCTTTAGTG	PATH2476	*wcrC*^{47A}-FLAG ORF
O1389_	TTTGTCATCATCATCTTTATAATCTTAACGATTATCCGCTCCTATTTCTTC		
O1393	GATTATAAAGATGATGATGACAAAAATGTACGATAAATTAGTAACAATCATAG	PATH2009	*wcrC*^{39} 3’
P1840	TGCAGATACCGTAGCAACTTT		
For construction of Δ*whaI*^{39}::P-*erm*			
P2080	AGATGCCGATCAGAGAATAAAGG	PATH2009	*whaI*^{39} 5’
P2082	CATTATCCATTAAAAATCAAACGGATCCTATATAAATAATTTTAACAACTC		
P1	TAGGATCCGTTTGATTTTTAATGGATAATG	P-*erm* cassette	P-*erm*
P2	GGGCCCCTTTCCTTATGCTTTTG		
P2083	CAAAAGCATAAGGAAAGGGGCCCGAACACAGAGTTCCTCATCAATATAGAGTC	PATH2009	*whaI*^{39} 3’
P2753	CGGATTTGTTGATTCCAATTCCA		
For construction of Δ*bgaA*::P-*spec-rpsL*$^{+}$			
P23	CCGTAGAACCACTATCACAAG	D39	*bgaA* 5’
P24	TTATCCATTAAAAATCAAACGGATCCTATCCCACAGCAAACTTACGAATGCTATAAACTC		
P1	TAGGATCCGTTTGATTTTTAATGGATAATG	P-*spec-rpsL*+ cassette	P-*spec-rpsL*$^{+}$
P2	GGGCCCCTTTCCTTATGCTTTTG		
P25	AAAAGCATAAGGAAAGGGGCCCTTAGCTCTTCTAGGTTTGAGTGCAGGATTAGTAGTTAC	D39	*bgaA* 3’
P26	GACGAAACTTTGCGGATTTG		
For construction of Δ*bgaA*::P-*kan-wcrO*^{34}			
P3	ATTTGCCCATCTGGCTGACTAGGAGGAA	NUS0016	

O357	CTTATATAACTTTGTATCTTCTTCCATCTAC ATTCTCCTGTGTTTTTTATTTTTGGTG		*bgaA*::P-*kan*
O358	ATGGAAGAAGATACAAAGTTATATAAG	PATH1748	*wcrO*^{34}
O359	TCACTCTGCATCACCTACTGATATAAG		
O360	CTTATATCAGTAGGTGATGCAGAGTGACA AAAGCATAAGGAAAGGGGCCC	D39	*bgaA 3'*
P26	GACGAAACTTTGCGGATTTG		
For construction of Δ*whaI*^{39} <> *whaI*^{47F}			
P2080	AGATGCCGATCAGAGAATAAAGG	PATH2009	*whaI*^{39} *5'*
P3189	TTATTGACCAATTTCTCTCAATCTTTTC		
P3192	GATTGAGAGAAATTGGTCAATAAATGTCG GATATAAAAATCATTC	PATH2475	*whaI*^{47F}
P3193	GCTTGCGCTTTTCTAATTTCATTCTTCATTA TCACCAAGAG		
P3191	AATTAGAAAAGCGCAAGCATATTATTTG	PATH2009	*whaI*^{39} *3'*
P2081	CCACCATCTGTACCGTCTATAATC		
For construction of Δ*whaI*^{39} <> *whaI*^{47A}			
P2080	AGATGCCGATCAGAGAATAAAGG	PATH2009	*whaI*^{39} *5'*
P3189	TTATTGACCAATTTCTCTCAATCTTTTC		
P3194	GATTGAGAGAAATTGGTCAATAAATGTCA GATATAAAAATTATTC	PATH2476	*whaI*^{47A}
P3193	GCTTGCGCTTTTCTAATTTCATTCTTCATTA TCACCAAGAG		
P3191	AATTAGAAAAGCGCAAGCATATTATTTG	PATH2009	*whaI*^{39} *3'*
P2081	CCACCATCTGTACCGTCTATAATC		
For construction of Δ*bgaA*::P_{Zn}-*wcrC*^{34}			
P86	GTTTGACTGCCGGTGTATCT	AF24	*bgaA*::P_{czcD} 5'
P147	ATTTCTCATTCCTTTGTTATAATAG		
P3132	CTATTATAACAAAGGAATGAGAAATGTGA AGGGTAGGATAG	PATH1748	*wcrC*^{34}
P3133	TTAGACATTTTCCCCCCCAGATAC		
P3134	GTATCTGGGGGGGAAAATGTCTAATTAGC TCTTCTAGGTTTGAG	D39	*bgaA* 3'
P6	TGCATGGTTACGATAGTCTTGG		
For construction of Δ*wciU*^{18A}::P-*sacB-kan-rpsL*$^{+}$			
O310	TCAGGGACTACTGAGGATGTT	PATH4560	*wciU*^{18A} *5'*
O311	CATTATCCATTAAAAATCAAACGGATCCT ATTTAACCAAACCACCTGTTCGCTG		
P1	TAGGATCCGTTTGATTTTTAATGGATAATG	SpnYL001	P-*sacB-kan-rpsL*$^{+}$
P2	GGGCCCCTTTCCTTATGCTTTTG		
O312	CAAAAGCATAAGGAAAGGGGCCCATGGA AAAACATGTAGAAGAAGTTATTAG	PATH4560	*wciU*^{18A} *3'*
O313	GGTAAGCGATGGATTGGATAGT		
For construction of Δ*wciU*^{18A}::*wciU*^{16F}			
O310	TCAGGGACTACTGAGGATGTT	PATH4560	*wciU*^{18A} *5'*
O1618	TTTTTAAATAATTTCTCGTAGT		
O1603	ACTACGAGAAATTATTTAAAAAATGAAAA TATTACATTATACTTTAGGAT	PATH1702	*wciU*^{16F}

O1604	GACATATCTTTAATCATTCTCCTTAACTAT CATTTATCACTCTTTCATAA		
O1605	GGAGAATGATTAAAGATATGTC	PATH4560	*wciU*^{18A} *3'*
O313	GGTAAGCGATGGATTGGATAGT		
For construction of Δ*bgaA*::P$_{Zn}$-*wciU*^{18A}			
P86	GTTTGACTGCCGGTGTATCT	AF24	*bgaA*-P$_{czcD}$ 5'
P147	ATTTCTCATTCCTTTGTTATAATAG		
O301	CTATTATAACAAAGGAATGAGAAATATGA AAATACTACATTATACTTTAGGATTTC	PATH4560	*wciU*^{18A}
O293	TTAATTGTTGCTTCTAACCTGTTTG		
O302	CAAACAGGTTAGAAGCAACAATTAATTAG CTCTTCTAGGTTTGAGTGCAGGATTAG	D39	*bgaA* 3'
P6	TGCATGGTTACGATAGTCTTGG		
For construction of Δ*wcrC*^{34}::P-*sacB-kan-rpsL*$^+$			
O346	TGCCTACAGGGAATGTCTTTAC	PATH1748	*wcrC*^{34} *5'*
O347	CATTAAAAATCAAACGGATCCTAATCATC CAAAATCCATTTCAC		
P1	TAGGATCCGTTTGATTTTTAATGGATAATG	SpnYL001	P-*sacB-kan-rpsL*$^+$
P2	GGGCCCCTTTCCTTATGCTTTTG		
O348	GCATAAGGAAAGGGGCCCCGAATTATACA TCAGTGGATAGATTTG	PATH1748	*wcrC*^{34} *3'*
O349	CTTTCATACCTGCACTCTCCTATT		
For construction of Δ*wcrC*^{47A}::P-*sacB-kan-rpsL*$^+$			
O354	GGACTGTCGTAGTGGCTATATG	PATH2476	*wcrC*^{47A} *5'*
O353	CCATTAAAAATCAAACGGATCCTAAACTC GCTCACCACCGCCAG		
P1	TAGGATCCGTTTGATTTTTAATGGATAATG	SpnYL001	P-*sacB-kan-rpsL*$^+$
P2	GGGCCCCTTTCCTTATGCTTTTG		
O355	CAAAAGCATAAGGAAAGGGGCCCAATAA AATTCTTAATCAGTGG	PATH2476	*wcrC*^{47A} *3'*
O356	GAGCTTTGAATCAGCCCTTTG		
For construction of ΔCEP::P-*kan-wcrB*^{10C}			
P3	ATTTGCCCATCTGGCTGACTAGGAGGAA	IU5122	CEP 5'
P2398	CAAGAAATAAAATAATAGCACCTACATTC TCCTGTGTTTTTTA		
O483	GTGCTATTATTTTATTTCTTGGAG	PATH2460	*wcrB*^{10C}
O536	TTACTCGCTAGGGAAGGAGATTTC		
O537	GAAATCTCCTTCCCTAGCGAGTAACAAAA GCATAAGGAAAGGGGC	IU5122	CEP 5'
P4	TGGGCACGTCATTTCCAATGATTACCTG		
For construction of ΔCEP::P-*kan-wciO*^{33B}			
P3	ATTTGCCCATCTGGCTGACTAGGAGGAA	IU5122	CEP 5'
P2397	CTATACTTTTAACACTTACCATCTACATTC TCCTGTGTTTTTTA		
P2392	ATGGTAAGTGTTAAAAGTATAGAAC	PATH1945	*wcrB*^{33B}
P2387	TTATTTTACTATCTTTTTAATAGAG		

P2393	CTATTAAAAGATAGTAAAATAACAAAAGCATAAGGAAAGGGGCC	IU5122	CEP 3'
P4	TGGGCACGTCATTTCCAATGATTACCTG		
For construction of Δ*whaI*^{39} <> *wcrO*^{34}			
P2080	AGATGCCGATCAGAGAATAAAGG	PATH2009	*whaI*^{39} *5'*
P3189	TTATTGACCAATTTCTCTCAATCTTTTC		
P3190	GAGAAATTGGTCAATAAATGGAAGAAGATACAAAGTTATATAAG	PATH1748	*wcrO*^{34}
P3191	GCTTGCGCTTTTCTAATTTCACTCTGCATCACCTACTGATATAAG		
P3192	AATTAGAAAAGCGCAAGCATATTATTTG	PATH2009	*whaI*^{39} *3'*
P2081	CCACCATCTGTACCGTCTATAATC		
For construction of Δ*whaI*^{39} <> *wcrB*^{10F}			
P2080	AGATGCCGATCAGAGAATAAAGG	PATH2009	*whaI*^{39} *5'*
P3189	TTATTGACCAATTTCTCTCAATCTTTTC		
O548	GATTGAGAGAAATTGGTCAATAAGTGCTATTATTTTATTTTTTGG	PATH1539	*wcrB*^{10F}
O549	GCTTGCGCTTTTCTAATTTTACTCGTTAGGGAAGGAAATTTC		
P3192	AATTAGAAAAGCGCAAGCATATTATTTG	PATH2009	*whaI*^{39} *3'*
P2081	CCACCATCTGTACCGTCTATAATC		
For construction of Δ*whaI*^{39} <> *wcrB*^{10B}			
P2080	AGATGCCGATCAGAGAATAAAGG	PATH2009	*whaI*^{39} *5'*
P3189	TTATTGACCAATTTCTCTCAATCTTTTC		
O550	GATTGAGAGAAATTGGTCAATAAGTGCTATTATTTTATTTCTTGG	PATH2459	*wcrB*^{10B}
O551	GCTTGCGCTTTTCTAATTTTACTCATTAGGGAAGGAGATTTC		
P3192	AATTAGAAAAGCGCAAGCATATTATTTG	PATH2009	*whaI*^{39} *3'*
P2081	CCACCATCTGTACCGTCTATAATC		
For construction of Δ*wcrC*^{34} <> *wcrC*^{34} $^{\text{S102F}}$			
O346	TGCCTACAGGGAATGTCTTTAC	PATH1748	*wcrC*^{34} *5'*
O513	CAATTTCTATCCTACCCTTCACTCTGCATCACCTACTGATATAAG		
O369	GTGAAGGGTAGGATAGAAATTG	NUS1607	*wcrC*$^{34(S102F)}$
O362	TTAGACATTTTCCCCCCCAGATAC		
O514	GTATCTGGGGGGGAAAATGTCTAATAAAATACTGACGATTACTG	PATH1748	*wcrC*^{34} *3'*
O349	CTTTCATACCTGCACTCTCCTATT		
For construction of Δ*wcrC*^{47A} <> *wcrC*^{47A} $^{\text{S96F}}$			
O354	GGACTGTCGTAGTGGCTATATG	PATH2476	*wcrC*^{47A} *5'*
O517	CACTAAAGCGATTTTTTTCATGTTTCAACTACTCTCATTCTTC		
O455	ATGAAAAAAATCGCTTTAGTG	PATH2476	*wcrC*$^{47A(S96F)}$
O456	TTAACGATTATCCGCTCCTATTTC		
O518	GAAATAGGAGCGGATAATCGTTAAAATGAAAAAAATGTTAATTTG	PATH2476	*wcrC*^{47A} *3'*
O356	GAGCTTTGAATCAGCCCTTTG		
For construction of Δ*cps2I-K*::P-*erm*			

P47	GCAGTTAAATGGATACAAACGATGA	D39	*cps2I* 5’
O1904	CATTATCCATTAAAAATCAAACGGATCCT AAGATAAAAAATATAAGATACTTTTTGTC		
P1	TAGGATCCGTTTGATTTTTAATGGATAATG	P-*erm* cassette	P-*erm*
P2	GGGCCCCTTTCCTTATGCTTTTG		
P1122	CAAAAGCATAAGGAAAGGGGCCCAACTCA TTAGAAGATGTCAAGGAAAAAGTCTATAC AC	D39	*cps2K* 5’
P1123	CAACTTGAACATTTTGCATGCGTTGAACC		
For construction of Δ*cpsE*::P-*spec-rpsL*$^+$			
P64	ACCAGCTACGACTCCTTCTTCT	NUS0029	Δ*cpsE*::P-*spec-rpsL*$^+$
P65	TCGTCCCACCACTAGATAATAGCC		
For construction of Δ*cpsE*			
P64	ACCAGCTACGACTCCTTCTTCT	HMS0002	Δ*cpsE*
P65	TCGTCCCACCACTAGATAATAGCC		
For construction of Δ*ugd*::P-*spec-rpsL*$^+$			
P288	CCTTTGAGCCAACAGCTACA	D39	*ugd 5’*
P289	CCATTAAAAATCAAACGGATCCTATGCAA ATAGTAACGCGTAGGCCAGCCCAACATGC		
P1	TAGGATCCGTTTGATTTTTAATGGATAATG	P-*spec-rpsL*+ cassette	P-*spec-rpsL*$^+$
P2	GGGCCCCTTTCCTTATGCTTTTG		
P290	GCATAAGGAAAGGGGCCCAGGGGCCTTGA AGCTTATAAAGATAAAGTTTACACAAG	D39	*ugd 5’*
P291	GCTCAAGAAGGGCATGGTATT		
For construction of Δ*ugd*			
P288	CCTTTGAGCCAACAGCTACA	D39	*ugd 5’*
O2801	CTCTTATCCAGATCCCCCTTGTGTAATGCC CCAAACCAATTACTGCTATATTC		
O2800	GAATATAGCAGTAATTGGTTTGGGGCATT ACACAAGGGGGATCTGGATAAGAG	D39	*ugd 5’*
P291	GCTCAAGAAGGGCATGGTATT		
For construction of ΔCEP::P-*spec-rpsL*$^+$			
P3	ATTTGCCCATCTGGCTGACTAGGAGGAA	D39	CEP 5’
P2693	CAAACGGATCCTACTCGAGCTTAGCTGAC TTCAACCCACTACAG		
P1	TAGGATCCGTTTGATTTTTAATGGATAATG	P-*spec-rpsL*$^+$ cassette	P-*spec-rpsL*$^+$
P2	GGGCCCCTTTCCTTATGCTTTTG		
P2694	TAAACGTCCAAAAGCATAAGGAAAGGGGC CCGTCGCTTTTCATTA	D39	CEP 3’
P4	TGGGCACGTCATTTCCAATGATTACCTG		
For construction of ΔCEP::P_{Zn}-*ytgP*			
P3	ATTTGCCCATCTGGCTGACTAGGAGGAA	D39	CEP 5’
O1150	GGGTTTTTTTTCCATGGATGCATGCTACTC GAGCTTAGCTGACTTCAACC		
O97	GCATGCATCCATGGAAAAAAAACCC	NUS2224	P_{Zn}-*ytgP*
P1099	TTACGAAAGCTTAAATTTTGCTCGC		
O1151	GCGAGCAAAATTTAAGCTTTCGTAAGGAA AGGGGCCCGTCGCTTTTCATT	D39	CEP 3’

P4	TGGGCACGTCATTTCCAATGATTACCTG		
For construction of Δ*ytgP*::P-*erm*			
P1289	ATCCCAGAACCAGAGCAATC	D39	*ytgP* 5'
P1290	CATTATCCATTAAAAATCAAACGGATCCT ACGTTAGCCAAGCAGTCCCCCGTAACATC		
P1	TAGGATCCGTTTGATTTTTAATGGATAATG	P-*erm* cassette	P-*erm*
P2	GGGCCCCTTTCCTTATGCTTTTG		
P1291	CAAAAGCATAAGGAAAGGGGCCCGATAA GGTAATAGGAAAAGCCCAAGCAGATC	D39	*ytgP* 3'
P1292	TTGAGAACTGCTTCCTGAGTC		
For construction of Δ*bgaA*::P-*kan-cps23BJ* variants			
P23	CCGTAGAACCACTATCACAAG	NUS1936 (*2*)	*bgaA*::P-*kan-cps23BJ*
P6	TGCATGGTTACGATAGTCTTGG		
For construction of ΔCEP::P-*kan-wchU*^{19C}			
P3	ATTTGCCCATCTGGCTGACTAGGAGGAA	HMS0019	CEP::P-*kan*
O728	TTCTTGGAATTACAATCTTCAACTACATTC TCCTGTGTTTTTTA		
O729	TTGAAGATTGTAATTCCAAGAATTA	PATH2463	*wchU*^{19C}
O730	TTATAGATTGTTGTTCATATCTTGC		
O731	AAGATATGAACAACAATCTATAAGGAAAG GGGCCCGTCGCTTTTC	HMS0019	CEP′
P4	TGGGCACGTCATTTCCAATGATTACCTG		
For construction of Δ *wchU*^{19C}::P-*erm*			
P3255	CTCGGAGAACCTATGAAACACA	PATH2463	*wchU*^{19C} *5′*
P3256	CCATTAAAAATCAAACGGATCCTAGGACC AATCCCAGGTCAGTTG		
P1	TAGGATCCGTTTGATTTTTAATGGATAATG	P-*erm* cassette	P-*erm*
P2	GGGCCCCTTTCCTTATGCTTTTG		
P3257	CAAAAGCATAAGGAAAGGGGCCCGACAC AGTTGCCAAAAGAATG	PATH2463	*wchU*^{19C} *3′*
P3258	ACGAGGCAAATCTTGAGGAG		
For construction of Δ*cps14J*::P-*erm*			
O1747	CGTGTCAACAGTATTGTAACTGGA	NUH0007	*cps14J* 5'
O1792	CATTATCCATTAAAAATCAAACGGATCCT AGGCTAAATTTTTACTGATTTTATTACTC		
P1	TAGGATCCGTTTGATTTTTAATGGATAATG	P-*erm* cassette	P-*erm*
P2	GGGCCCCTTTCCTTATGCTTTTG		
O1793	CCAAAAGCATAAGGAAAGGGGCCCCGAA AGATTGGTTATGAAAATATATTATAA	NUH0007	*cps14J* 3'
O1750	GGTGCTGACGAATAGTCTGAAAT		
For construction of Δ*cps2J*::P-*erm*			
O93	GAACATGGAAATGTGGAAGATGAG	D39	*cps2J* 5'
O2064	CCATTAAAAATCAAACGGATCCTATTTTAG TAAGTAATTATATCTTCTAC		
P1	TAGGATCCGTTTGATTTTTAATGGATAATG	P-*erm* cassette	P-*erm*
P2	GGGCCCCTTTCCTTATGCTTTTG		
O2065	CAAAAGCATAAGGAAAGGGGCCCATTGGT GAATTAAAAAAGTTTCTAAC	D39	*cps2J* 3'

O94	CATAGCCGAAGGAAGGATTGT		
For construction of Δ*cps23BJ*::P-*erm*			
O646	TGAGAAATCGAGTCTATGGATCTTAC	PATH212(*5*)	*cps23BJ* 5’
O2192	CCATTAAAAATCAAACGGATCCTAATTTTT TGCTAATTCCTTATATTTACTC		
P1	TAGGATCCGTTTGATTTTTAATGGATAATG	P-*erm* cassette	P-*erm*
P2	GGGCCCCTTTCCTTATGCTTTTG		
O2193	CAAAAGCATAAGGAAAGGGGCCCTGTTTA AAATTATTAAAAGGATTTAAATG	PATH212	*cps23BJ* 3’
O649	CATTCACATATCTGACATAAGGCTTG		
For construction of Δ*cps2I*::P-*erm*			
P47	GCAGTTAAATGGATACAAACGATGA	D39	*cps2I* 5’
O1904	CATTATCCATTAAAAATCAAACGGATCCT AAGATAAAAAATATAAGATACTTTTTGTC		
P1	TAGGATCCGTTTGATTTTTAATGGATAATG	P-*erm* cassette	P-*erm*
P2	GGGCCCCTTTCCTTATGCTTTTG		
O1905	CGTCCAAAAGCATAAGGAAAGGGGCCCAT GCAACTATATAAGGAACTAGAAAATTG	D39	*cps2I* 3’
P48	TCCACCAGCAAATCCTGAAATA		
For construction of Δ*cps14E*::P-*sacB-kan-*$rpsL^+$			
O2312	CAGTATTACTCGTGTTTCTGATGTG	NUH0007	*cps14E 5'*
O2313	CATTATCCATTAAAAATCAAACGGATCCT ACAGAAAAATTTCCAATCCTTTTTTATCC		
P1	TAGGATCCGTTTGATTTTTAATGGATAATG	P-*sacB-kan-*$rpsL^+$ cassette	P-*sacB-kan-*$rpsL^+$
P2	GGGCCCCTTTCCTTATGCTTTTG		
O2314	CGTCCAAAAGCATAAGGAAAGGGGCCCGT TGTATTTATGAGAAATGGAGCGAAG	NUH0007	*cps14E 3'*
O2315	CAAATTGCATCTGGTGATCATTTAC		
For construction of Δ*cps2J<>*$wzxC^{A33V}$*-FLAG*			
P51	GAACATGGAAATGTGGAAGATGAG	D39	*cps2J 5’*
P272	CGCCGCTGATGGTTTTTCACGTAAGCTCA TTTTTCTAGTTCCTTATATAGTTGC		
P273	ATGAGCTTACGTGAAAAAACCATCA	CS3/pDF3(*4*)	$wzxC^{A33V}$
P274	TCATTTATCATCATCATCTTTATAA		
P275	CGAGGATTATAAAGATGATGATGATAAAT GAGAACCAATAAGTACGAGTATTGAAAGG	D39	*cps2J 3’*
P54	CATAGCCGAAGGAAGGATTGT		
For construction of Δ*cps2J<>wzk*			
P36	ATGACAAAAAGTATCTTATATTTTT	D39	*cps2J* 5’
O2622	GAATTTTATGTTTTTTTTTCGCCATTTTTCT AGTTCCTTATATAGTTGCATG		
O2623	ATGGCGAAAAAAAAACATAAAATTC	gBlock(*40*)	*wzk*
P1464	TTAGCCGAGATTGTCTTTGTGTTGG		
P1465	CCAACACAAAGACAATCTCGGCTAAGAAC CAATAAGTACGAGTATTGAAAGG	D39	*cps2J* 3’
P300	ATCATAGCGATTAGCAACAATGGC		
For construction of Δ*bgaA::PspxB-*$cps23BJ^{P254S}$			
O95	CCGTAGAACCACTATCACAAG		

P1787	AATGATAACTCTCCTTCAATTTTTTAAAC	NUS1257(*28*)	*bgaA'-PspxB*
O1148	AAAAAATTGAAGGAGAGTTATCATTATGAGTAAATATAAGGAATTAGCAA	NUS1963(*5*)	*cps23BJ*^{P254S}
O119	TCATTTAAATCCTTTTAATAATTTT		
O1149	AAAATTATTAAAAGGATTTAAATGATTAGCTCTTCTAGGTTTGAGTGCAG	D39	*bgaA'*
O6	TGCATGGTTACGATAGTCTTGG		
For construction of Δ*bgaA::PspxB-cps23BJ*^{D231G}			
O95	CCGTAGAACCACTATCACAAG	NUS1257(*28*)	*bgaA'-PspxB*
P1787	AATGATAACTCTCCTTCAATTTTTTAAAC		
O1148	AAAAAATTGAAGGAGAGTTATCATTATGAGTAAATATAAGGAATTAGCAA	NUS2002(*5*)	*cps23BJ*^{D231G}
O119	TCATTTAAATCCTTTTAATAATTTT		
O1149	AAAATTATTAAAAGGATTTAAATGATTAGCTCTTCTAGGTTTGAGTGCAG	D39	*bgaA'*
O6	TGCATGGTTACGATAGTCTTGG		
For construction of Δ*cps14E*			
O2312	CAGTATTACTCGTGTTTCTGATGTG	NUH0007	*cps14E 5'*
O2669	CTTCGCTCCATTTCTCATAAATACAACCAGAAAAATTTCCAATCCTTTTTATCC		
O2670	GGATAAAAAAGGATTGGAAATTTTTCTGGTTGTATTTATGAGAAATGGAGCGAAG	NUH0007	*cps14E 3'*
O2315	CAAATTGCATCTGGTGATCATTTAC		
For construction of pCS190 [P_{T7}-*ggta1*Δ74]			
O866	ACCACCATATGCAACGTAACGAGGACGAGTCTAAAC	gBlock	*ggta1*
O867	CTCGACAAGCTTTCACACATTATTTCGCACTACATTATATTCTTTGG		
O925	GTTTAGACTCGTCCTCGTTACGTTGCATATGGTGGTGGTGGTGGTGAGAACCACGCATGG	pTD68	pTD68
O926	ATAATGTAGTGCGAAATAATGTGTGAAAGCTTGTCGAGCACCACCACCACCACCACTGAG		
For construction of Δ*whaD*^{5}::P-*erm*			
O2704	GTCAATGATGGATCTACAG	NUS0327	*whaD*^{5} 5’
O2705	CATTATCCATTAAAAATCAAACGGATCCTATAACATAACCCTAAAAGGAGC		
P1	TAGGATCCGTTTGATTTTTAATGGATAATG	P-*erm* cassette	P-*erm*
P2	GGGCCCCTTTCCTTATGCTTTTG		
O2706	CAAAAGCATAAGGAAAGGGGCCCTATAATCTGGAAGGTTCAGC	NUS0327	*whaD*^{5} 3’
O2707	CGCACTACAATATTTTTCTGAC		
For construction of Δ*wcwH*^{7A}::P-*erm*			
O2865	CTGGATGCAAACATAGTTC	NUS0667	*wcwH*^{7A} 5’
O2866	TATCCATTAAAAATCAAACGGATCCTAAATTTTATATCCTCCCGTAG		
P1	TAGGATCCGTTTGATTTTTAATGGATAATG	P-*erm*	P-*erm*

P2	GGGCCCCTTTCCTTATGCTTTTG	cassette	
O2867	AAGCATAAGGAAAGGGGCCCGGATGGGA GAAAACTAC	NUS0667	*wcwH*^{7A} 3’
O2868	CCTCTCCTAAACCAATACC		
For construction of Δ*wcwD*^{7F}::P-*erm*			
O2708	CAAGCACCAATCCCGATC	NUS0310	*wcwD*^{7F} 5’
O2709	CACATTATCCATTAAAAATCAAACGGATC CTAATAAGGAAAAAAGGTTGGTAG		
P1	TAGGATCCGTTTGATTTTTAATGGATAATG	P-*erm* cassette	P-*erm*
P2	GGGCCCCTTTCCTTATGCTTTTG		
O2710	CGTCCAAAAGCATAAGGAAAGGGGCCCAA ATTAAAAAAAGAAAAATGGAC	NUS0310	*wcwD*^{7F} 3’
O2711	CCTGAGAAATGACGCTATC		
For construction of Δ*wcwH*^{7F}::P-*erm*			
O2865	CTGGATGCAAACATAGTTC	NUS0310	*wcwH*^{7F} 5’
O2866	TATCCATTAAAAATCAAACGGATCCTAAA TTTTATATCCTCCCGTAG		
P1	TAGGATCCGTTTGATTTTTAATGGATAATG	P-*erm* cassette	P-*erm*
P2	GGGCCCCTTTCCTTATGCTTTTG		
O2867	AAGCATAAGGAAAGGGGCCCGGATGGGA GAAAACTAC	NUS0310	*wcwH*^{7F} 3’
O2868	CCTCTCCTAAACCAATACC		
For construction of Δ*wcrH*^{10C}::P-*erm*			
O1111	CCCAAGAAGTCAAGGACAAGAT	NUS0315	*wcrH*^{10C} 5’
O1112	CATTAAAAATCAAACGGATCCTAATTTCCT GCATTCTTTTCGTA		
P1	TAGGATCCGTTTGATTTTTAATGGATAATG	P-*erm* cassette	P-*erm*
P2	GGGCCCCTTTCCTTATGCTTTTG		
O1113	CAAAAGCATAAGGAAAGGGGCCCGTAGG AGTAGCGATTCGAAGTG	NUS0315	*wcrH*^{10C} 3’
O1114	CCTGAGATTATGTCTTGGGATGAG		
For construction of Δ*wcrH*^{10F}::P-*erm*			
O1111	CCCAAGAAGTCAAGGACAAGAT	NUS0280	*wcrH*^{10F} 5’
O1112	CATTAAAAATCAAACGGATCCTAATTTCCT GCATTCTTTTCGTA		
P1	TAGGATCCGTTTGATTTTTAATGGATAATG	P-*erm* cassette	P-*erm*
P2	GGGCCCCTTTCCTTATGCTTTTG		
O1113	CAAAAGCATAAGGAAAGGGGCCCGTAGG AGTAGCGATTCGAAGTG	NUS0280	*wcrH*^{10F} 3’
O1114	CCTGAGATTATGTCTTGGGATGAG		
For construction of Δ*wchX*^{15A}::P-*erm*			
O2524	CTCATTTCTTAGATGGAGCTAGGG	NUS0999	*wchX*^{15A} 5’
O677	CATTAAAAATCAAACGGATCCTAACTGAT AATAACCATAAAAAC		
P1	TAGGATCCGTTTGATTTTTAATGGATAATG	P-*erm* cassette	P-*erm*
P2	GGGCCCCTTTCCTTATGCTTTTG		
O678	CAAAAGCATAAGGAAAGGGGCCCCCAATC TTTAATGGAACCGC	NUS0999	*wchX*^{15A} 3’
O676	GTTCCTAACCCTGCTGCTAAA		

For construction of $\Delta wchN^{15B}$::P-*erm*			
O2619	TGGTGGTTTATCAGATGCTCG	NUS0340	$wchN^{15B}$ 5’
O2520	CCATTAAAAATCAAACGGATCCTATTTAAT AGAATCTAAAATTTTTCTATCAT		
P1	TAGGATCCGTTTGATTTTTAATGGATAATG	P-*erm* cassette	P-*erm*
P2	GGGCCCCTTTCCTTATGCTTTTG		
O2521	GTCCAAAAGCATAAGGAAAGGGGCCCTAT AAGTTCATAGTATCTTCGAAAGTTTAG	NUS0340	$wchN^{15B}$ 3’
O2620	CCATAAATCTGTGCCTTGACG		
For construction of $\Delta wchX^{15B}$::P-*erm*			
O3654	GGCTACCAGGAAAAATTG	NUS0340	$wchX^{15B}$ 5’
O2526	CCATTAAAAATCAAACGGATCCTATAAAA ATTTAACATTTTGTAAAACAACCAT		
P1	TAGGATCCGTTTGATTTTTAATGGATAATG	P-*erm* cassette	P-*erm*
P2	GGGCCCCTTTCCTTATGCTTTTG		
O2527	CAAAAGCATAAGGAAAGGGGCCCGTAGG AAGGAGAATAATTCAAGAGTTGTGA	NUS0340	$wchX^{15B}$ 3’
O2525	TTCATGCAGATAGGTATAGCGATTA		
For construction of $\Delta wchN^{15C}$::P-*erm*			
O2619	TGGTGGTTTATCAGATGCTCG	NUS0289	$wchN^{15C}$ 5’
O2520	CCATTAAAAATCAAACGGATCCTATTTAAT AGAATCTAAAATTTTTCTATCAT		
P1	TAGGATCCGTTTGATTTTTAATGGATAATG	P-*erm* Cassette	P-*erm*
P2	GGGCCCCTTTCCTTATGCTTTTG		
O2521	GTCCAAAAGCATAAGGAAAGGGGCCCTAT AAGTTCATAGTATCTTCGAAAGTTTAG	NUS0289	$wchN^{15C}$ 3’
O2620	CCATAAATCTGTGCCTTGACG		
For construction of $\Delta wchX^{15C}$::P-*erm*			
O3654	GGCTACCAGGAAAAATTG	NUS0289	$wchX^{15C}$ 5’
O2526	CCATTAAAAATCAAACGGATCCTATAAAA ATTTAACATTTTGTAAAACAACCAT		
P1	TAGGATCCGTTTGATTTTTAATGGATAATG	P-*erm* cassette	P-*erm*
P2	GGGCCCCTTTCCTTATGCTTTTG		
O2527	CAAAAGCATAAGGAAAGGGGCCCGTAGG AAGGAGAATAATTCAAGAGTTGTGA	NUS0289	$wchX^{15C}$ 3’
O2525	TTCATGCAGATAGGTATAGCGATTA		
For construction of $\Delta wcxP^{16F}$::P-*erm*			
O3300	CCAATAATTGTTATGGGAGG	NUS0292	$wcxP^{16F}$ 5’
O3301	CCATTAAAAATCAAACGGATCCTACGAAC TACTAATGTATAAAT		
P1	TAGGATCCGTTTGATTTTTAATGGATAATG	P-*erm* cassette	P-*erm*
P2	GGGCCCCTTTCCTTATGCTTTTG		
O3302	AAAAGCATAAGGAAAGGGGCCCTCTAGTG AAACTCAGAAG	NUS0292	$wcxP^{16F}$ 3’
O3303	TTCTGGTCCAAGAGAAATAG		
For construction of $\Delta wcxQ^{16F}$::P-*erm*			
O3304	TTGCTCGTCAGAGATTTG	NUS0292	$wcxQ^{16F}$ 5’
O3305	CATTAAAAATCAAACGGATCCTATTTTACA AAGTCTAAAATTT		

P1	TAGGATCCGTTTGATTTTTAATGGATAATG	P-*erm* cassette	P-*erm*
P2	GGGCCCCTTTCCTTATGCTTTTG		
O3306	AAGCATAAGGAAAGGGGCCCTTACCACCA ATAGAAAAAAG	NUS0292	*wcxQ*^{16F} 3'
O3307	GCCTTTTGTAGCATATTGG		
For construction of Δ*wciW*^{18B}::P-*erm*			
O1605	GGAGAATGATTAAAGATATGTC	NUS0330	*wciW*^{18B} 5'
O3308	CATTATCCATTAAAAATCAAACGGATCCT AAACAGGAAAAAACGTTCC		
P1	TAGGATCCGTTTGATTTTTAATGGATAATG	P-*erm* cassette	P-*erm*
P2	GGGCCCCTTTCCTTATGCTTTTG		
O3309	CCAAAAGCATAAGGAAAGGGGCCCTATAA AAAAAATTACAATG	NUS0330	*wciW*^{18B} 3'
O3310	CTACTGGCCCTAAATTCTTC		
For construction of Δ*wciY*^{18B}::P-*erm*			
O3099	GGTGTATTTATGAAGACAGA	NUS0330	*wciY*^{18B} 5'
O3100	CCATTAAAAATCAAACGGATCCTACAATA AATGCAACGGTAATG		
P1	TAGGATCCGTTTGATTTTTAATGGATAATG	P-*erm* cassette	P-*erm*
P2	GGGCCCCTTTCCTTATGCTTTTG		
O3101	CAAAAGCATAAGGAAAGGGGCCCTCAGTA CAGTGTGCTAATTG	NUS0330	*wciY*^{18B} 3'
O3102	GGTCAATAAAACCCAGTTTG		
For construction of Δ*wchR*^{19B}::P-*erm*			
O3311	CGGAGAAAACTATCTTCACG	NUS0317	*wchR*^{19B} 5'
O3312	CATTATCCATTAAAAATCAAACGGATCCT ATGGACGTTGTTTTATCCAA		
P1	TAGGATCCGTTTGATTTTTAATGGATAATG	P-*erm* cassette	P-*erm*
P2	GGGCCCCTTTCCTTATGCTTTTG		
O3313	CGTCCAAAAGCATAAGGAAAGGGGCCCAA AAGTAATACTTGGGAAAAAAG	NUS0317	*wchR*^{19B} 3'
O3314	CCAAATTGAACGAATAACTC		
For construction of Δ*wchR*^{19C}::P-*erm*			
O3311	CGGAGAAAACTATCTTCACG	NUS0316	*wchR*^{19C} 5'
O3312	CATTATCCATTAAAAATCAAACGGATCCT ATGGACGTTGTTTTATCCAA		
P1	TAGGATCCGTTTGATTTTTAATGGATAATG	P-*erm* cassette	P-*erm*
P2	GGGCCCCTTTCCTTATGCTTTTG		
O3313	CGTCCAAAAGCATAAGGAAAGGGGCCCAA AAGTAATACTTGGGAAAAAAG	NUS0316	*wchR*^{19C} 3'
O3314	CCAAATTGAACGAATAACTC		
For construction of Δ*wchW*^{23A}::P-*erm*			
O3319	GTTAGTATTATTCTGCCCGT	NUS0399	*wchW*^{23A} 5'
O3320	CATTATCCATTAAAAATCAAACGGATCCT ATAATTTATAAAAATCATTC		
P1	TAGGATCCGTTTGATTTTTAATGGATAATG	P-*erm* cassette	P-*erm*
P2	GGGCCCCTTTCCTTATGCTTTTG		
O3321	CCAAAAGCATAAGGAAAGGGGCCCAATA GTGAATTCGAGAAGAG	NUS0399	*wchW*^{23A} 3'

O3322	CCTATACAACCTGAAATTGC		
For construction of Δ*wchX*^{23A}::P-*erm*			
O3103	GAGGTTCCTAATGAAAGATG	NUS0399	*wchX*^{23A} 5’
O3104	CCATTAAAAATCAAACGGATCCTAACTTA TCAAAAACATAAAAACG		
P1	TAGGATCCGTTTGATTTTTAATGGATAATG	P-*erm* cassette	P-*erm*
P2	GGGCCCCTTTCCTTATGCTTTTG		
O3105	GTCCAAAAGCATAAGGAAAGGGGCCCCCA ATCTTTAATGGAACCG	NUS0399	*wchX*^{23A} 3’
O2525	TTCATGCAGATAGGTATAGCGATTA		
For construction of Δ*wchX*^{23B}::P-*erm*			
O3193	GATAAAATAGGTGTAACGGC	NUS0329	*wchX*^{23B} 5’
O3194	CATTATCCATTAAAAATCAAACGGATCCT ACAATAGCATAAAAATAGTTCTAG		
P1	TAGGATCCGTTTGATTTTTAATGGATAATG	P-*erm* cassette	P-*erm*
P2	GGGCCCCTTTCCTTATGCTTTTG		
O3105	GTCCAAAAGCATAAGGAAAGGGGCCCCCA ATCTTTAATGGAACCG	NUS0329	*wchX*^{23B} 3’
O2525	TTCATGCAGATAGGTATAGCGATTA		
For construction of Δ*wchW*^{23F}::P-*erm*			
O3319	GTTAGTATTATTCTGCCCGT	NUS0389	*wchW*^{23F} 5’
O3320	CATTATCCATTAAAAATCAAACGGATCCT ATAATTTATAAAAATCATTC		
P1	TAGGATCCGTTTGATTTTTAATGGATAATG	P-*erm* cassette	P-*erm*
P2	GGGCCCCTTTCCTTATGCTTTTG		
O3321	CCAAAAGCATAAGGAAAGGGGCCCAATA GTGAATTCGAGAAGAG	NUS0389	*wchW*^{23F} 3’
O3322	CCTATACAACCTGAAATTGC		
For construction of Δ*wchX*^{23F}::P-*erm*			
O3103	GAGGTTCCTAATGAAAGATG	NUS0389	*wchX*^{23F} 5’
O3104	CCATTAAAAATCAAACGGATCCTAACTTA TCAAAAACATAAAAACG		
P1	TAGGATCCGTTTGATTTTTAATGGATAATG	P-*erm* cassette	P-*erm*
P2	GGGCCCCTTTCCTTATGCTTTTG		
O3105	GTCCAAAAGCATAAGGAAAGGGGCCCCCA ATCTTTAATGGAACCG	NUS0389	*wchX*^{23F} 3’
O2525	TTCATGCAGATAGGTATAGCGATTA		
For construction of Δ*wcxG*^{24B}::P-*erm*			
O310	TCAGGGACTACTGAGGATGTT	NUS0247	*wcxG*^{24B} 5’
O3323	CATTATCCATTAAAAATCAAACGGATCCT ACAATTCGTCATCATCTAAAAA		
P1	TAGGATCCGTTTGATTTTTAATGGATAATG	P-*erm* cassette	P-*erm*
P2	GGGCCCCTTTCCTTATGCTTTTG		
O3324	GTCCAAAAGCATAAGGAAAGGGGCCCCCT CCTGTTGATAAGAGAG	NUS0247	*wcxG*^{24B} 3’
O3325	TTAGTCGCATCATAGAACAC		
For construction of Δ*rbsF*^{24B}::P-*erm*			
O3326	CCTATTTCATTGCTTTTGGA	NUS0247	*rbsF*^{24B} 5’

O3327	CATTATCCATTAAAAATCAAACGGATCCT AAAAAAAATCAAAAGCAATAT		
P1	TAGGATCCGTTTGATTTTTAATGGATAATG	P-*erm* cassette	P-*erm*
P2	GGGCCCCTTTCCTTATGCTTTTG		
O3328	CCAAAAGCATAAGGAAAGGGGCCCTACGG ATATTTTGTATATCT	NUS0247	*rbsF*^{24B} 3’
O3329	CATTGCTAAAACTGCCTG		
For construction of Δ*wcxG*^{24F}::P-*erm*			
O310	TCAGGGACTACTGAGGATGTT	NUS0311	*wcxG*^{24F} 5’
O3323	CATTATCCATTAAAAATCAAACGGATCCT ACAATTCGTCATCATCTAAAAA		
P1	TAGGATCCGTTTGATTTTTAATGGATAATG	P-*erm* cassette	P-*erm*
P2	GGGCCCCTTTCCTTATGCTTTTG		
O3324	GTCCAAAAGCATAAGGAAAGGGGCCCCCT CCTGTTGATAAGAGAG	NUS0311	*wcxG*^{24F} 3’
O3325	TTAGTCGCATCATAGAACAC		
For construction of Δ*rbsF*^{24F}::P-*erm*			
O3326	CCTATTTCATTGCTTTTGGA	NUS0311	*rbsF*^{24F} 5’
O3327	CATTATCCATTAAAAATCAAACGGATCCT AAAAAAAATCAAAAGCAATAT		
P1	TAGGATCCGTTTGATTTTTAATGGATAATG	P-*erm* cassette	P-*erm*
P2	GGGCCCCTTTCCTTATGCTTTTG		
O3328	CCAAAAGCATAAGGAAAGGGGCCCTACGG ATATTTTGTATATCT	NUS0311	*rbsF*^{24F} 3’
O3329	CATTGCTAAAACTGCCTG		
For construction of Δ*whaL*^{27}::P-*erm*			
O3330	GGAGTAGGGATGTTACCT	NUS0463	*whaL*^{27} 5’
O3331	CATTATCCATTAAAAATCAAACGGATCCT AATACAAAAATAGATCGTCAC		
P1	TAGGATCCGTTTGATTTTTAATGGATAATG	P-*erm* cassette	P-*erm*
P2	GGGCCCCTTTCCTTATGCTTTTG		
O3332	CCAAAAGCATAAGGAAAGGGGCCCCTAGA TGAACGACTAATGAG	NUS0463	*whaL*^{27} 3’
O3333	GTGTAAATTTCCCACCAAC		
For construction of Δ*wcrN*^{27}::P-*erm*			
O1027	GTGAGTATTTTTGCCACATTGAT	NUS0463	*wcrN*^{27} 5’
O3064	CCATTAAAAATCAAACGGATCCTAAATCG TATGTAAAATCTCCAAC		
P1	TAGGATCCGTTTGATTTTTAATGGATAATG	P-*erm* cassette	P-*erm*
P2	GGGCCCCTTTCCTTATGCTTTTG		
O3065	AAAGCATAAGGAAAGGGGCCCATTTTACC TCCTGAAAAGGAT	NUS0463	*wcrN*^{27} 3’
O2879	CATAAACCGGCATAAGTTG		
For construction of Δ*wcxP*^{28A}::P-*erm*			
O3334	GTGGGGCTATTTATAGGTAT	NUS0671	*wcxP*^{28A} 5’
O3335	TCCATTAAAAATCAAACGGATCCTATGGA AAAGTTGAGGTATAAA		
P1	TAGGATCCGTTTGATTTTTAATGGATAATG	P-*erm* cassette	P-*erm*
P2	GGGCCCCTTTCCTTATGCTTTTG		

O3302	AAAAGCATAAGGAAAGGGGCCCTCTAGTG AAACTCAGAAG	NUS0671	*wcxP*^{28A} 3’
O3336	CTGAAAGAGCAACTGGAG		
For construction of Δ*wcxQ*^{28A}::P-*erm*			
O3337	GCTTATTCTTTTTGGGCTAC	NUS0671	*wcxQ*^{28A} 5’
O3338	TATCCATTAAAAATCAAACGGATCCTATTT TACAAAGTCTAAAATTT		
P1	TAGGATCCGTTTGATTTTTAATGGATAATG	P-*erm* cassette	P-*erm*
P2	GGGCCCCTTTCCTTATGCTTTTG		
O3339	CAAAAGCATAAGGAAAGGGGCCCTTACCA CCAATAGAAAAAAGAG	NUS0671	*wcxQ*^{28A} 3’
O676	GTTCCTAACCCTGCTGCTAAA		
For construction of Δ*wcxP*^{28F}::P-*erm*			
O3334	GTGGGGCTATTTATAGGTAT	NUS0390	*wcxP*^{28F} 5’
O3335	TCCATTAAAAATCAAACGGATCCTATGGA AAAGTTGAGGTATAAA		
P1	TAGGATCCGTTTGATTTTTAATGGATAATG	P-*erm* cassette	P-*erm*
P2	GGGCCCCTTTCCTTATGCTTTTG		
O3302	AAAAGCATAAGGAAAGGGGCCCTCTAGTG AAACTCAGAAG	NUS0390	*wcxP*^{28F} 3’
O3336	CTGAAAGAGCAACTGGAG		
For construction of Δ*wcxQ*^{28F}::P-*erm*			
O3337	GCTTATTCTTTTTGGGCTAC	NUS0390	*wcxQ*^{28F} 5’
O3338	TATCCATTAAAAATCAAACGGATCCTATTT TACAAAGTCTAAAATTT		
P1	TAGGATCCGTTTGATTTTTAATGGATAATG	P-*erm* cassette	P-*erm*
P2	GGGCCCCTTTCCTTATGCTTTTG		
O3339	CAAAAGCATAAGGAAAGGGGCCCTTACCA CCAATAGAAAAAAGAG	NUS0390	*wcxQ*^{28F} 3’
O676	GTTCCTAACCCTGCTGCTAAA		
For construction of Δ*wchQ*^{32A}::P-*erm*			
O3340	GGTTTTGCTACATATGGATC	NUS1098	*wchQ*^{32A} 5’
O3341	CCATTAAAAATCAAACGGATCCTAATTGA CGCATTGTTTAGTTT		
P1	TAGGATCCGTTTGATTTTTAATGGATAATG	P-*erm* cassette	P-*erm*
P2	GGGCCCCTTTCCTTATGCTTTTG		
O3342	CAAAAGCATAAGGAAAGGGGCCCTGTCTA CTGCAGTCAACA	NUS1098	*wchQ*^{32A} 3’
O3344	GACTTTTCAAATCCTACACC		
For construction of Δ*wcrN*^{32A}::P-*erm*			
O288	GTGAGTATTTTTTCAACATTGA	NUS1098	*wcrN*^{32A} 5’
O2877	CCATTAAAAATCAAACGGATCCTAAATCG TGTGTAAAATCTCCA		
P1	TAGGATCCGTTTGATTTTTAATGGATAATG	P-*erm* cassette	P-*erm*
P2	GGGCCCCTTTCCTTATGCTTTTG		
O2878	GTCCAAAAGCATAAGGAAAGGGGCCCATT TTGCCTCCAGAGAAG	NUS1098	*wcrN*^{32A} 3’
O2879	CATAAACCGGCATAAGTTG		
For construction of Δ*wchQ*^{32F}::P-*erm*			

O3340	GGTTTTGCTACATATGGATC	NUS0342	*wchQ*^{32F} 5’
O3341	CCATTAAAAATCAAACGGATCCTAATTGA CGCATTGTTTAGTTT		
P1	TAGGATCCGTTTGATTTTTAATGGATAATG	P-*erm* cassette	P-*erm*
P2	GGGCCCCTTTCCTTATGCTTTTG		
O3342	CAAAAGCATAAGGAAAGGGGCCCTGTCTA CTGCAGTCAACA	NUS0342	*wchQ*^{32F} 3’
O3344	GACTTTTCAAATCCTACACC		
For construction of Δ*wcrN*^{32F}::P-*erm*			
O288	GTGAGTATTTTTTCAACATTGA	NUS0342	*wcrN*^{32F} 5’
O2877	CCATTAAAAATCAAACGGATCCTAAATCG TGTGTAAAATCTCCA		
P1	TAGGATCCGTTTGATTTTTAATGGATAATG	P-*erm* cassette	P-*erm*
P2	GGGCCCCTTTCCTTATGCTTTTG		
O2878	GTCCAAAAGCATAAGGAAAGGGGCCCATT TTGCCTCCAGAGAAG	NUS0342	*wcrN*^{32F} 3’
O2879	CATAAACCGGCATAAGTTG		
For construction of Δ*wciF*^{33A}::P-*erm*			
O2788	CATTTACTCCTGCTATTGCTC	NUS0332	*wciF*^{33A} 5’
O2789	CATTAAAAATCAAACGGATCCTAATCCAA CGCAAAGCGCAGATA		
P1	TAGGATCCGTTTGATTTTTAATGGATAATG	P-*erm* cassette	P-*erm*
P2	GGGCCCCTTTCCTTATGCTTTTG		
O2790	AAAGCATAAGGAAAGGGGCCCCTTTCCCC AAATTACTAC	NUS0332	*wciF*^{33A} 3’
O2791	CTAAGACCGTCTGAAATACC		
For construction of Δ*wciF*^{33D}::P-*erm*			
O2781	GACAAGAGAATACATGAAATTG	NUS0672	*wciF*^{33D} 5’
O2782	CCATTAAAAATCAAACGGATCCTATTCCAT TGCATAATGTAGATA		
P1	TAGGATCCGTTTGATTTTTAATGGATAATG	P-*erm* cassette	P-*erm*
P2	GGGCCCCTTTCCTTATGCTTTTG		
O2783	CAAAAGCATAAGGAAAGGGGCCCTTTTTA CTTTTCCCTAAAGGAT	NUS0672	*wciF*^{33D} 3’
O2880	CATTATTCAATCCGTGTCC		
For construction of Δ*wciF*^{33F}::P-*erm*			
O2788	CATTTACTCCTGCTATTGCTC	NUS0326	*wciF*^{33F} 5’
O2789	CATTAAAAATCAAACGGATCCTAATCCAA CGCAAAGCGCAGATA		
P1	TAGGATCCGTTTGATTTTTAATGGATAATG	P-*erm* cassette	P-*erm*
P2	GGGCCCCTTTCCTTATGCTTTTG		
O2790	AAAGCATAAGGAAAGGGGCCCCTTTCCCC AAATTACTAC	NUS0326	*wciF*^{33F} 3’
O2791	CTAAGACCGTCTGAAATACC		
For construction of Δ*wcrK*^{35C}::P-*erm*			
O3195	CAGGAGATGCTTATAGTTGC	NUS0369	*wcrK*^{35C} 5’
O3196	CCATTAAAAATCAAACGGATCCTAAGCAC GTAAAACCACATT		
P1	TAGGATCCGTTTGATTTTTAATGGATAATG	P-*erm*	P-*erm*

P2	GGGCCCCTTTCCTTATGCTTTTG	cassette	
O3197	AAGCATAAGGAAAGGGGCCCTTAGATAAT GTTGCTGAAGA	NUS0369	*wcrK*^{35C} 3’
O3198	CATCACCCTTACCAACAAC		
For construction of Δ*wcrW*^{41A}::P-*erm*			
O2881	CATTAGGACAATTTAATCCG	NUS0374	*wcrW*^{41A} 5’
O2882	CATTATCCATTAAAAATCAAACGGATCCT AATGAAAAAAATTTTTTTCTA		
P1	TAGGATCCGTTTGATTTTTAATGGATAATG	P-*erm* cassette	P-*erm*
P2	GGGCCCCTTTCCTTATGCTTTTG		
O2883	GTCCAAAAGCATAAGGAAAGGGGCCCCTT GATACAATTGTAGATG	NUS0374	*wcrW*^{41A} 3’
O2884	GAAACAAAAGTACTCCCAC		
For construction of Δ*wcrQ*^{41A}::P-*erm*			
O3345	GCGAACTCAGATATATGTGG	NUS0374	*wcrQ*^{41A} 5’
O3346	CCATTAAAAATCAAACGGATCCTAAGGTA TTCGTTTTGTTACTT		
P1	TAGGATCCGTTTGATTTTTAATGGATAATG	P-*erm* cassette	P-*erm*
P2	GGGCCCCTTTCCTTATGCTTTTG		
O3347	CCAAAAGCATAAGGAAAGGGGCCCAAAA ATGGTAGGGAAACGG	NUS0374	*wcrQ*^{41A} 3’
O3348	GCTATTCTTATACCAAGCAC		

REFERENCES AND NOTES

1. L. L. Lairson, B. Henrissat, G. J. Davies, S. G. Withers, Glycosyltransferases: Structures, functions, and mechanisms. *Annu. Rev. Biochem.* **77**, 521–555 (2008).

2. B. Imperiali, Bacterial carbohydrate diversity—A brave new world. *Curr. Opin. Chem. Biol.* **53**, 1–8 (2019).

3. C. Whitfield, S. S. Wear, C. Sande, Assembly of bacterial capsular polysaccharides and exopolysaccharides. *Annu. Rev. Microbiol.* **74**, 521–543 (2020).

4. L.-T. Sham, S. Zheng, A. A. Yakhnina, A. C. Kruse, T. G. Bernhardt, Loss of specificity variants of WzxC suggest that substrate recognition is coupled with transporter opening in MOP-family flippases. *Mol. Microbiol.* **109**, 633–641 (2018).

5. W.-Z. Chua, M. Maiwald, K. L. Chew, R. T.-P. Lin, S. Zheng, L.-T. Sham, High-throughput mutagenesis and cross-complementation experiments reveal substrate preference and critical residues of the capsule transporters in *Streptococcus pneumoniae*. *mBio* **12**, e0261521 (2021).

6. S. Kumar, A. Mollo, D. Kahne, N. Ruiz, The bacterial cell wall: From lipid II flipping to polymerization. *Chem. Rev.* **11**, 8884–8910 (2022).

7. T. Su, R. Nakamoto, Y.-Y. Chun, W.-Z. Chua, J.-H. Chen, J. J. Zik, L.-T. Sham, Decoding capsule synthesis in Streptococcus pneumoniae. *FEMS Microbiol. Rev.* **44**, fuaa067 (2020).

8. K. A. Geno, G. L. Gilbert, J. Y. Song, I. C. Skovsted, K. P. Klugman, C. Jones, H. B. Konradsen, M. H. Nahm, Pneumococcal capsules and their types: Past, present, and future. *Clin. Microbiol. Rev.* **28**, 871–899 (2015).

9. O. Rendueles, J. A. M. de Sousa, A. Bernheim, M. Touchon, E. P. C. Rocha, Genetic exchanges are more frequent in bacteria encoding capsules. *PLOS Genet.* **14**, e1007862 (2018).

10. A. J. van Tonder, R. A. Gladstone, S. W. Lo, M. H. Nahm, M. du Plessis, J. Cornick, B. Kwambana-Adams, S. A. Madhi, P. A. Hawkins, R. Benisty, R. Dagan, D. Everett, M. Antonio, K. P. Klugman, A. von Gottberg, R. F. Breiman, L. McGee, S. D. Bentley; The Global Pneumococcal Sequencing

Consortium, Putative novel cps loci in a large global collection of pneumococci. *Microb. Genom.* **5**, e000274 (2019).

11. N. J. Croucher, S. R. Harris, C. Fraser, M. A. Quail, J. Burton, M. van der Linden, L. McGee, A. von Gottberg, J. H. Song, K. S. Ko, B. Pichon, S. Baker, C. M. Parry, L. M. Lambertsen, D. Shahinas, D. R. Pillai, T. J. Mitchell, G. Dougan, A. Tomasz, K. P. Klugman, J. Parkhill, W. P. Hanage, S. D. Bentley, Rapid pneumococcal evolution in response to clinical interventions. *Science* **331**, 430–434 (2011).

12. M. P. Lisboa, N. Khan, C. Martin, F.-F. Xu, K. Reppe, A. Geissner, S. Govindan, M. Witzenrath, C. L. Pereira, P. H. Seeberger, Semisynthetic glycoconjugate vaccine candidate against *Streptococcus pneumoniae* serotype 5. *Proc. Natl. Acad. Sci. U.S.A.* **114**, 11063–11068 (2017).

13. B. Lepenies, J. Yin, P. H. Seeberger, Applications of synthetic carbohydrates to chemical biology. *Curr. Opin. Chem. Biol.* **14**, 404–411 (2010).

14. Z. Wang, Z. S. Chinoy, S. G. Ambre, W. Peng, R. McBride, R. P. de Vries, J. Glushka, J. C. Paulson, G.-J. Boons, A general strategy for the chemoenzymatic synthesis of asymmetrically branched N-glycans. *Science* **341**, 379–383 (2013).

15. W. Kightlinger, K. E. Duncker, A. Ramesh, A. H. Thames, A. Natarajan, J. C. Stark, A. Yang, L. Lin, M. Mrksich, M. P. DeLisa, M. C. Jewett, A cell-free biosynthesis platform for modular construction of protein glycosylation pathways. *Nat. Commun.* **10**, 5404 (2019).

16. K. Yamatsugu, R. A. Splain, L. L. Kiessling, Fidelity and promiscuity of a mycobacterial glycosyltransferase. *J. Am. Chem. Soc.* **138**, 9205–9211 (2016).

17. W. Kightlinger, L. Lin, M. Rosztoczy, W. Li, M. P. DeLisa, M. Mrksich, M. C. Jewett, Design of glycosylation sites by rapid synthesis and analysis of glycosyltransferases. *Nat. Chem. Biol.* **14**, 627–635 (2018).

18. K. W. Moremen, R. S. Haltiwanger, Emerging structural insights into glycosyltransferase-mediated synthesis of glycans. *Nat. Chem. Biol.* **15**, 853–864 (2019).

19. R. Taujale, A. Venkat, L.-C. Huang, Z. Zhou, W. Yeung, K. M. Rasheed, S. Li, A. S. Edison, K. W. Moremen, N. Kannan, Deep evolutionary analysis reveals the design principles of fold A glycosyltransferases. *eLife* **9**, e54532 (2020).

20. R. Taujale, Z. Zhou, W. Yeung, K. W. Moremen, S. Li, N. Kannan, Mapping the glycosyltransferase fold landscape using interpretable deep learning. *Nat. Commun.* **12**, 5656 (2021).

21. J. L. Czlapinski, C. R. Bertozzi, Synthetic glycobiology: Exploits in the Golgi compartment. *Curr. Opin. Chem. Biol.* **10**, 645–651 (2006).

22. T. Jaroentomeechai, M. N. Taw, M. Li, A. Aquino, N. Agashe, S. Chung, M. C. Jewett, M. P. DeLisa, Cell-free synthetic glycobiology: Designing and engineering glycomolecules outside of living cells. *Front. Chem.* **8**, 645 (2020).

23. W. Kightlinger, K. F. Warfel, M. P. DeLisa, M. C. Jewett, Synthetic glycobiology: Parts, systems, and applications. *ACS Synth. Biol.* **9**, 1534–1562 (2020).

24. Y. Narimatsu, H. J. Joshi, R. Nason, J. Van Coillie, R. Karlsson, L. Sun, Z. Ye, Y.-H. Chen, K. T. Schjoldager, C. Steentoft, S. Furukawa, B. A. Bensing, P. M. Sullam, A. J. Thompson, J. C. Paulson, C. Büll, G. J. Adema, U. Mandel, L. Hansen, E. P. Bennett, A. Varki, S. Y. Vakhrushev, Z. Yang, H. Clausen, An atlas of human glycosylation pathways enables display of the human glycome by gene engineered cells. *Mol. Cell* **75**, 394–407.e5 (2019).

25. Y. Narimatsu, C. Büll, Y.-H. Chen, H. H. Wandall, Z. Yang, H. Clausen, Genetic glycoengineering in mammalian cells. *J. Biol. Chem.* **296**, 100448 (2021).

26. T. R. Larson, J. Yother, Membrane linkage of a *Streptococcus pneumoniae* Wzy capsular polysaccharide occurs through an acylglycerol. bioRxiv 2020.09.16.299636 [**Preprint**]. 17 September 2020. https://doi.org/10.1101/2020.09.16.299636.

27. J. Yother, Capsules of Streptococcus pneumoniae and other bacteria: Paradigms for polysaccharide biosynthesis and regulation. *Annu. Rev. Microbiol.* **65**, 563–581 (2011).

28. R. Nakamoto, J. M. C. Kwan, J. F. L. Chin, H. T. Ong, J. Flores-Kim, C. Midonet, M. S. VanNieuwenhze, X. L. Guan, L.-T. Sham, The bacterial tyrosine kinase system CpsBCD governs the length of capsule polymers. *Proc. Natl. Acad. Sci. U.S.A.* **118**, e2103377118 (2021).

29. B. Xayarath, J. Yother, Mutations blocking side chain assembly, polymerization, or transport of a Wzy-dependent Streptococcus pneumoniae capsule are lethal in the absence of suppressor mutations and can affect polymer transfer to the cell wall. *J. Bacteriol.* **189**, 3369–3381 (2007).

30. A. Mavroidi, D. Godoy, D. M. Aanensen, D. A. Robinson, S. K. Hollingshead, B. G. Spratt, Evolutionary genetics of the capsular locus of serogroup 6 pneumococci. *J. Bacteriol.* **186**, 8181–8192 (2004).

31. M. B. Oliver, C. Jones, T. R. Larson, J. J. Calix, E. R. Zartler, J. Yother, M. H. Nahm, *Streptococcus pneumoniae* serotype 11D has a bispecific glycosyltransferase and expresses two different capsular polysaccharide repeating units. *J. Biol. Chem.* **288**, 21945–21954 (2013).

32. I. H. Park, K. A. Geno, J. Yu, M. B. Oliver, K. H. Kim, M. H. Nahm, Genetic, biochemical, and serological characterization of a new pneumococcal serotype, 6H, and generation of a pneumococcal strain producing three different capsular repeat units. *Clin. Vaccine Immunol.* **22**, 313–318 (2015).

33. F. Ganaie, K. Maruhn, C. Li, R. J. Porambo, P. L. Elverdal, C. Abeygunwardana, M. van der Linden, J. Ø. Duus, C. L. Sheppard, M. H. Nahm, Structural, genetic, and serological elucidation of *Streptococcus pneumoniae* serogroup 24 serotypes: Discovery of a new serotype, 24c, with a variable capsule structure. *J. Clin. Microbiol.* **59**, e0054021 (2021).

34. H. An, C. Qian, Y. Huang, J. Li, X. Tian, J. Feng, J. Hu, Y. Fang, F. Jiao, Y. Zeng, X. Huang, X. Meng, X. Liu, X. Lin, Z. Zeng, M. Guilliams, A. Beschin, Y. Chen, Y. Wu, J. Wang, M. R. Oggioni, J. Leong, J.-W. Veening, H. Deng, R. Zhang, H. Wang, J. Wu, Y. Cui, J.-R. Zhang, Functional vulnerability of liver macrophages to capsules defines virulence of blood-borne bacteria. *J. Exp. Med.* **219**, e20212032 (2022).

35. D. M. Aanensen, A. Mavroidi, S. D. Bentley, P. R. Reeves, B. G. Spratt, Predicted functions and linkage specificities of the products of the Streptococcus pneumoniae capsular biosynthetic loci. *J. Bacteriol.* **189**, 7856–7876 (2007).

36. R. J. Mostowy, N. J. Croucher, N. De Maio, C. Chewapreecha, S. J. Salter, P. Turner, D. M. Aanensen, S. D. Bentley, X. Didelot, C. Fraser, Pneumococcal capsule synthesis locus *cps* as evolutionary hotspot with potential to generate novel serotypes by recombination. *Mol. Biol. Evol.* **34**, 2537–2554 (2017).

37. F. Ganaie, J. S. Saad, L. McGee, A. J. van Tonder, S. D. Bentley, S. W. Lo, R. A. Gladstone, P. Turner, J. D. Keenan, R. F. Breiman, M. H. Nahm, A new pneumococcal capsule type, 10D, is the 100th serotype and has a large *cps* fragment from an oral Streptococcus. *mBio* **11**, e00937-20 (2020).

38. J. Jumper, R. Evans, A. Pritzel, T. Green, M. Figurnov, O. Ronneberger, K. Tunyasuvunakool, R. Bates, A. Žídek, A. Potapenko, A. Bridgland, C. Meyer, S. A. A. Kohl, A. J. Ballard, A. Cowie, B. Romera-Paredes, S. Nikolov, R. Jain, J. Adler, T. Back, S. Petersen, D. Reiman, E. Clancy, M. Zielinski, M. Steinegger, M. Pacholska, T. Berghammer, S. Bodenstein, D. Silver, O. Vinyals, A. W. Senior, K. Kavukcuoglu, P. Kohli, D. Hassabis, Highly accurate protein structure prediction with AlphaFold. *Nature* **596**, 583–589 (2021).

39. W.-L. Ng, Y. Wei, L. J. Perez, J. Cong, T. Long, M. Koch, M. F. Semmelhack, N. S. Wingreen, B. L. Bassler, Probing bacterial transmembrane histidine kinase receptor-ligand interactions with natural and synthetic molecules. *Proc. Natl. Acad. Sci. U.S.A.* **107**, 5575–5580 (2010).

40. W. Elhenawy, R. M. Davis, J. Fero, N. R. Salama, M. F. Felman, N. Ruiz, The O-antigen flippase Wzk can substitute for MurJ in peptidoglycan synthesis in *Helicobacter pylori* and *Escherichia coli*. *PLOS ONE* **11**, e0161587 (2016).

41. Y.-G. Yang, M. Sykes, Xenotransplantation: Current status and a perspective on the future. *Nat. Rev. Immunol.* **7**, 519–531 (2007).

42. J. Fang, J. Li, X. Chen, Y. Zhang, J. Wang, Z. Guo, W. Zhang, L. Yu, K. Brew, P. G. Wang, Highly efficient chemoenzymatic synthesis of α-galactosyl epitopes with a recombinant α(1→3)-galactosyltransferase. *J. Am. Chem. Soc.* **120**, 6635–6638 (1998).

43. Y.-Y. Chun, K. S. Tan, L. Yu, M. Pang, M. H. M. Wong, R. Nakamoto, W.-Z. Chua, A. Huee-Ping Wong, Z. Z. R. Lew, H. H. Ong, V. T. Chow, T. Tran, D. Yun Wang, L.-T. Sham, Influence of glycan structure on the colonization of *Streptococcus pneumoniae* on human respiratory epithelial cells. *Proc. Natl. Acad. Sci. U.S.A.* **120**, e2213584120 (2023).

44. L. L. Kiessling, M. B. Kraft, A path to complex carbohydrates. *Science* **341**, 357–358 (2013).

45. A. Varki, Biological roles of glycans. *Glycobiology* **27**, 3–49 (2017).

46. J. D. Valderrama-Rincon, A. C. Fisher, J. H. Merritt, Y.-Y. Fan, C. A. Reading, K. Chhiba, C. Heiss, P. Azadi, M. Aebi, M. P. DeLisa, An engineered eukaryotic protein glycosylation pathway in *Escherichia coli*. *Nat. Chem. Biol.* **8**, 434–436 (2012).

47. C. M. Harding, M. A. Nasr, N. E. Scott, G. Goyette-Desjardins, H. Nothaft, A. E. Mayer, S. M. Chavez, J. P. Huynh, R. L. Kinsella, C. M. Szymanski, C. L. Stallings, M. Segura, M. F. Feldman, A platform for glycoengineering a polyvalent pneumococcal bioconjugate vaccine using E. coli as a host. *Nat. Commun.* **10**, 891 (2019).

48. B. S. Hamilton, J. D. Wilson, M. A. Shumakovich, A. C. Fisher, J. C. Brooks, A. Pontes, R. Naran, C. Heiss, C. Gao, R. Kardish, J. Heimburg-Molinaro, P. Azadi, R. D. Cummings, J. H. Merritt, M. P. DeLisa, A library of chemically defined human N-glycans synthesized from microbial oligosaccharide precursors. *Sci. Rep.* **7**, 15907 (2017).

49. J. E. Martin, J. P. Lisher, M. E. Winkler, D. P. Giedroc, Perturbation of manganese metabolism disrupts cell division in *Streptococcus pneumoniae*. *Mol. Microbiol.* **104**, 334–348 (2017).

50. R. Junges, R. Khan, Y. Tovpeko, H. A. Åmdal, F. C. Petersen, D. A. Morrison, Markerless genome editing in competent Streptococci. *Methods Mol. Biol.* **1537**, 233–247 (2017).

51. C. K. Sung, H. Li, J. P. Claverys, D. A. Morrison, An *rpsL* cassette, Janus, for gene replacement through negative selection in *Streptococcus pneumoniae*. *Appl. Environ. Microbiol.* **67**, 5190–5196 (2001).

52. Y. Li, C. M. Thompson, M. Lipsitch, A modified Janus cassette (Sweet Janus) to improve allelic replacement efficiency by high-stringency negative selection in *Streptococcus pneumoniae*. *PLOS ONE* **9**, e100510 (2014).

53. A. Ducret, E. M. Quardokus, Y. V. Brun, MicrobeJ, a tool for high throughput bacterial cell detection and quantitative analysis. *Nat. Microbiol.* **1**, 16077 (2016).

54. F. Delaglio, S. Grzesiek, G. W. Vuister, G. Zhu, J. Pfeifer, A. Bax, NMRPipe: A multidimensional spectral processing system based on UNIX pipes. *J. Biomol. NMR* **6**, 277–293 (1995).

55. W. Lee, M. Tonelli, J. L. Markley, NMRFAM-SPARKY: Enhanced software for biomolecular NMR spectroscopy. *Bioinformatics* **31**, 1325–1327 (2015).

56. J. Santander, T. Martin, A. Loh, C. Pohlenz, D. M. Gatlin, R. Curtiss, Mechanisms of intrinsic resistance to antimicrobial peptides of Edwardsiella ictaluri and its influence on fish gut inflammation and virulence. *Microbiology (Reading)* **159**, 1471–1486 (2013).

57. Y. Li, D. M. Weinberger, C. M. Thompson, K. Trzcinski, M. Lipsitch, Surface charge of Streptococcus pneumoniae predicts serotype distribution. *Infect. Immun.* **81**, 4519–4524 (2013).

58. C. Gebus, C. Cottin, M. Randriantsoa, S. Drouillard, E. Samain, Synthesis of α-galactosyl epitopes by metabolically engineered *Escherichia coli*. *Carbohydr. Res.* **361**, 83–90 (2012).

59. W. Strober, Trypan blue exclusion test of cell viability. *Curr. Protoc. Immunol.* **111**, A3.B.1–A3.B.3 (2015).

60. P. R. Reeves, M. Hobbs, M. A. Valvano, M. Skurnik, C. Whitfield, D. Coplin, N. Kido, J. Klena, D. Maskell, C. R. Raetz, P. D. Rick, Bacterial polysaccharide synthesis and gene nomenclature. *Trends Microbiol.* **4**, 495–503 (1996).

61. B. O. Petersen, S. Meier, B. S. Paulsen, A. R. Redondo, I. C. Skovsted, Determination of native capsular polysaccharide structures of *Streptococcus pneumoniae* serotypes 39, 42, and 47F and comparison to genetically or serologically related strains. *Carbohydr. Res.* **395**, 38–46 (2014).

62. C. A. Bush, J. Yang, B. Yu, J. O. Cisar, Chemical Structures of *Streptococcus pneumoniae* capsular polysaccharide type 39 (CPS39), CPS47F, and CPS34 characterized by nuclear magnetic resonance spectroscopy and their relation to CPS10A. *J. Bacteriol.* **196**, 3271–3278 (2014).

63. F. L. Lin, E. Vinogradov, C. Deng, S. Zeller, L. Phelan, B. A. Green, K. U. Jansen, V. Pavliak, Structure elucidation of capsular polysaccharides from *Streptococcus pneumoniae* serotype 33C, 33D, and revised structure of serotype 33B. *Carbohydr. Res.* **383**, 97–104 (2014).

64. J. Yang, M. H. Nahm, C. A. Bush, J. O. Cisar, Comparative structural and molecular characterization of *Streptococcus pneumoniae* capsular polysaccharide serogroup 10. *J. Biol. Chem.* **286**, 35813–35822 (2011).

65. J. A. Lanie, W.-L. Ng, K. M. Kazmierczak, T. M. Andrzejewski, T. M. Davidsen, K. J. Wayne, H. Tettelin, J. I. Glass, M. E. Winkler, Genome sequence of Avery's virulent serotype 2 strain D39 of *Streptococcus pneumoniae* and comparison with that of unencapsulated laboratory strain R6. *J. Bacteriol.* **189**, 38–51 (2007).

66. K. M. Kazmierczak, K. J. Wayne, A. Rechtsteiner, M. E. Winkler, Roles of rel_{Spn} in stringent response, global regulation and virulence of serotype 2 *Streptococcus pneumoniae* D39. *Mol. Microbiol.* **72**, 590–611 (2009).
